# Development of the phenylpyrazolo[3,4-*d*]pyrimidine-based, insulin-like growth factor receptor/Src/AXL-targeting small molecule kinase inhibitor

**DOI:** 10.7150/thno.48865

**Published:** 2021-01-01

**Authors:** Ho Jin Lee, Phuong Chi Pham, Honglan Pei, Bumhee Lim, Seung Yeob Hyun, Byungyeob Baek, Byungjin Kim, Yunha Kim, Min-Hwan Kim, Nae-Won Kang, Hye-Young Min, Dae-Duk Kim, Jeeyeon Lee, Ho-Young Lee

**Affiliations:** 1 Creative Research Initiative Center for concurrent control of emphysema and lung cancer, College of Pharmacy, Seoul National University, Seoul 08826, Republic of Korea.; 2 College of Pharmacy and Research Institute of Pharmaceutical Sciences, Seoul National University, Seoul 08826, Republic of Korea.

**Keywords:** small molecule kinase inhibitor, type I insulin-like growth factor receptor, Src, AXL

## Abstract

**Rationale:** The type I insulin-like growth factor receptor (IGF-1R) signaling pathway plays key roles in the development and progression of numerous types of human cancers, and Src and AXL have been found to confer resistance to anti-IGF-1R therapies. Hence, co-targeting Src and AXL may be an effective strategy to overcome resistance to anti-IGF-1R therapies. However, pharmacologic targeting of these three kinases may result in enhanced toxicity. Therefore, the development of novel multitarget anticancer drugs that block IGF-1R, Src, and AXL is urgently needed.

**Methods:** We synthesized a series of phenylpyrazolo[3,4-*d*]pyrimidine (PP)-based compounds, wherein the PP module was conjugated with 2,4-bis-arylamino-1,3-pyrimidines (I2) via a copper(I)-catalyzed alkyne-azide cycloaddition reaction. To develop IGF-1R/Src/AXL-targeting small molecule kinase inhibitors, we selected LL6 as an active compound and evaluated its antitumor and antimetastatic effects *in vitro* and *in vivo* using the MTT assay, colony formation assays, migration assay, flow cytometric analysis, a tumor xenograft model, the *Kras^G12D/+^*-driven spontaneous lung tumorigenesis model, and a spontaneous metastasis model using Lewis lung carcinoma (LLC) allografts. We also determined the toxicity of LL6 *in vitro* and *in vivo*.

**Results:** LL6 induced apoptosis and suppressed viability and colony-forming capacities of various non-small cell lung cancer (NSCLC) cell lines and their sublines with drug resistance. LL6 also suppressed the migration of NSCLC cells at nontoxic doses. Administration of LL6 in mice significantly suppressed the growth of NSCLC xenograft tumors and metastasis of LLC allograft tumors with outstanding toxicity profiles. Furthermore, the multiplicity, volume, and load of lung tumors in *Kras^G12D/+^* transgenic mice were substantially reduced by the LL6 treatment.

**Conclusions:** Our results show the potential of LL6 as a novel IGF-1R/Src/AXL-targeting small molecule kinase inhibitor, providing a new avenue for anticancer therapies.

## Introduction

Cancer is the leading cause of human deaths worldwide 1. Despite extensive efforts to develop therapeutic interventions, the 5-year survival rate for certain types of cancers did not show much improvement 1. Conventional chemotherapy is a standard therapeutic option 2. However, resistance and toxicity of chemotherapy have led the focus of anticancer drug development to molecularly targeted therapy, in which anticancer drugs act on specific proteins, thereby reducing undesired side effects on normal cells 3. However, drug resistance due to the cross-talk between signaling pathways and high mutation rates of oncogenes are the main drawbacks of single targeted therapy. Inhibitors targeting epidermal growth factor receptor (EGFR) are one of the most widely developed molecularly targeted therapeutic options and have shown efficacy in a subset of patients with specific genetic abnormalities 4. However, most patients do not show a response to therapy because of primary resistance 4. Furthermore, acquired resistance to therapy frequently develops in primarily susceptible patients 4-6.

Accumulating evidence supports the role of the type I insulin-like growth factor receptor (IGF-1R) signaling pathway in resistance to chemotherapy, molecularly targeted therapy, and recently developed immunotherapy 7-11. Consequently, IGF-1R has been considered as an attractive therapeutic target, especially when combined with other anticancer treatments 10. However, anti-IGF-1R therapies have shown marginal efficacy because of inherent or acquired resistance 10, 12-16. Blockade of bypass mechanisms by combination therapies may circumvent drug resistance. However, potential severe side effects through drug-drug interactions, poor patient compliance, and complex pharmacokinetic and pharmacodynamic profiles are the main hurdles of combination therapies 17, 18. Thus, there is an urgent need to develop novel IGF-1R-targeting drugs that overcome resistance.

Canonical IGF-1R signaling activity is controlled by ligands (IGF1, IGF2, and insulin) binding to IGF-1R, insulin receptor (IR), or hybrid receptors of IGF-1R/IR 19. Additionally, Src, a non-receptor tyrosine kinase (non-RTK), has the ability to phosphorylate IGF-1R and IR at the same amino acid residues as the ligand-induced autophosphorylation sites 20. Increased levels of Src expression and kinase activity have been reported in various human cancers 21, 22 and often induce resistance to various molecularly targeted anticancer drugs, including IGF-1R inhibitor-based therapies 12. AXL, a RTK involved in various tumor activities 23-25, has also been implicated in the resistance to chemotherapy and anti-IGF-1R therapies 26, 27 and associated with poor prognosis in human cancers 28, 29.

A previous study revealed potent inhibition of AXL by bosutinib (SKI-606), which was originally developed as an Src and Abl kinase inhibitor 30. Pyrazolo[3,4-d]pyrimidines (PP), a well-known scaffold for Src, functions as an AXL inhibitor 31. Owing to its antitumor activity, bosutinib is currently under clinical evaluation as an AXL inhibitor 23. In addition, several inhibitors targeting AXL, including UNC569 32, UNC1062 33, and eSM134 31, or Src, including AD80 34, KB SRC4 35, PP2 36, PP121 37, and eCF506 38, possess the PP fragment. These findings provide the rationale for the potential use of Src tyrosine kinase inhibitors (TKIs) to discover novel AXL inhibitors. Indeed, in the current study, our docking analysis revealed structural similarity between the ATP-binding pockets of AXL and Src. Hence, we have attempted to develop small molecule kinase inhibitors (SMKIs) that target IGF-1R, Src, and AXL by utilizing PP and 2,4-bis-arylamino-1,3-pyrimidines modules (I2) as scaffolds directed for Src and IGF-1R, respectively. Here, we report a novel multikinase inhibitor, designated as LL6, which has broad antitumor effects on non-small cell lung cancer (NSCLC) cells, including those carrying de novo or acquired resistance to chemotherapy or EGFR TKIs, both *in vitro* and *in vivo,* by concurrently targeting IGF-1R, Src, and AXL. These results suggest that LL6 is a useful multitarget SMKI in the treatment of cancer.

## Materials and Methods

### Cell culture

Human lung cancer cell lines (A549, H1299, H1993, H1944, H226B, H226Br, H460, H522, HCC15, and PC9), a diploid human lung fibroblast cell line (Wi38), and the Lewis lung carcinoma (LLC) cell line were purchased from the American Type Culture Collection (ATCC, Manassas, VA, USA) or kindly provided by Dr. John V. Heymach (MD Anderson Cancer Center, Houston, TX, USA). Human retinal pigment epithelial (RPE) cells were kindly provided by Dr. Jeong Hun Kim (College of Medicine, Seoul National University, Seoul, Republic of Korea). HT-22 cells were provided by Dr. Dong Gyu Jo (College of Pharmacy, Sungkyunkwan University, Suwon, Republic of Korea). Lung cancer cells were cultured in RPMI 1640 medium supplemented with 10% fetal bovine serum (FBS) and antibiotics (Welgene, Gyeongsan-si, Republic of Korea). LLC, HT-22, and Wi38 cells were maintained in DMEM supplemented with 10% FBS and antibiotics. RPE cells were maintained in DMEM/F12 supplemented with 10% FBS and antibiotics. NSCLC cell lines with acquired resistance to chemotherapy (cisplatin-resistant H1299/CsR and pemetrexed-resistant H1299/PmR and H460/PmR) and molecular targeted therapy (erlotinib-resistant PC9/ER) were generated by continuous exposure to corresponding anticancer drugs for more than six months. Cells were maintained at 37 °C in a humidified atmosphere with 5% CO_2_. Cell lines were authenticated and validated using the AmpFLSTR identifier PCR Amplification Kit (Applied Biosystems, Foster City, CA, USA; cat. No. 4322288) in 2013 and 2016. Cells that had been passaged for < 6 months after receipt or resuscitation of validated cells were used in this study.

### Reagents

Antibodies against AXL, pIGF-1R (Y1135/6), IGF-1R, pSrc (Y416), Src, pMet (Y1234/5), Met, tubulin, and cleaved caspase-3 were purchased from Cell Signaling Technology (Danvers, MA, USA). Antibodies against cleaved PARP were purchased from BD Biosciences (San Jose, CA, USA). Antibodies against pAXL (Y702) were purchased from R&D systems (Minneapolis, MN, USA). Primary antibodies against IGF-1R and actin were purchased from Santa Cruz Biotechnology (Santa Cruz, CA, USA). Primary antibodies against pIR/IGF-1R (Y1162/3) were purchased from Thermo Fisher Scientific (Waltham, MA, USA). Horseradish peroxidase-conjugated secondary antibodies were purchased from GeneTex (Irvine, CA, USA). Linsitinib, dasatinib, and bemcentinib (R428) were purchased from Selleckchem (Houston, TX, USA). 3-(4,5-Dimethylthiazol-2-yl)-2,5-diphenyl tetrazolium bromide (MTT) and other chemicals were purchased from Sigma-Aldrich (St. Louis, MO, USA) unless otherwise specified. The detailed information on used primary and secondary antibodies, including vendor, catalogue number, application, and dilution ratio (or concentration) is listed in **Table S1.**

### Molecular docking simulations

Molecular docking analysis was implemented using the Surflex-Dock module in Sybyl-X2.2.1 (Tripos Inc, St Louis, MO, USA) with the known crystal structure of AXL complexed with ligands (PDB ID: 5U6B). To prepare the protein, hydrogen was added and energy was minimized using Powell's method with the Tripos force field until the root-mean-square derivation (RMSD) values were < 0.05 Kcal/mol·Å. Initial optimization and termination of minimization were set as simplex and gradient, respectively. The new ligands were prepared using Chem3D (PerkinElmer, Waltham, MA, USA). Molecular docking simulations were conducted using the Surflex-Dock mode with the extraction of the original ligand. To generate the active site, a threshold of 0.5 Å and bloat of 0 Å were applied based on the original ligand in the crystal structure. Other parameters were used as default. The results of the docking simulation were validated by comparing the redocked structure to the original pose of the ligand. Molecular interactions between proteins and ligands were further analyzed using Discovery Studio 4.0 Visualizer (BIOVIA, San Diego, CA, USA).

### MTT assay

Cells were seeded into 96-well plates at a density of 1-2 × 10^3^ cells/well and incubated for 24 h. Cells were treated with vehicle or various concentrations of test compounds for two (PC9 and PC9/ER cells) or three (other cells) days. Cells were further incubated with MTT solution (final concentration of 500 μg/mL) for 4 h at 37 °C. The formazan products were dissolved in dimethyl sulfoxide (DMSO), and the absorbance was measured at 570 nm. The data are presented as a percentage of the control group.

### Competition binding assay

*K*_d_ values were obtained by KINOMEscan from DiscoverX (San Diego, CA, USA). To briefly summarize, *K*_d_ was measured by a competition binding assay that quantitatively measures the ability of a compound to compete with an immobilized ligand. The ligand-displayed affinity bead and DNA (T7 bacteriophage)-tagged kinases are combined with a test compound, and the amount of fusion protein bound to the solid support is quantified by qPCR. Dose response curves of 11 points are obtained for *K*_d_ values, which are measured in duplicate. See more details in the cited references 39-42 and see also at www.discoverx.com.

### Anchorage-dependent colony formation assay

Cells were seeded into 6-well plates at a density of 300 cells/well and treated with LL6. The drug-containing medium was changed once or twice a week. After incubation for 10-14 days, colonies were fixed with 100% methanol, stained with 0.002% crystal violet solution, and washed with deionized water several times. The colonies were imaged and manually counted.

### Anchorage-independent colony formation assays

Cells (2-3 × 10^3^ cells/well) were mixed with sterile 1% agar solution (final concentration of 0.4%) and poured onto 1% base agar in 24-well plates. After solidification of the top agar, LL6 was diluted in complete medium and added to the agar. Cells embedded in the top agar were incubated for 10-14 days at 37 °C with 5% CO_2_. The LL6-containing medium was changed twice a week. After incubation, the colonies were stained with MTT solution, imaged, and counted using ImageJ software (National Institutes of Health, Bethesda, MA, USA).

### Immunofluorescence staining

H1944 cells, seeded onto coverslip, were treated with increasing concentrations of LL6 (0, 1, 2.5 μM) for 24 h. Cells were fixed with 4% paraformaldehyde for 10 min at room temperature, washed with PBS, and then permeabilized with 0.3% Triton X-100 for 15 min at room temperature. After washing cells with PBS, the cells were incubated with blocking solution [3% bovine serum albumin (BSA) in Tris-buffered saline containing 0.1% Tween-20] for 1 h at room temperature. Cells were incubated with primary antibodies (1:200 dilution) at 4 ºC overnight. Cells were washed several times with PBS and incubated with fluorochrome-conjugated secondary antibodies (Thermo Fisher Scientific) for 1 h at room temperature. Cells were washed multiple times with PBS and counterstained with 4′,6-diamidino-2-phenylindole (DAPI). The coverslips were mounted with mounting solution (Dako, Glostrup, Denmark) and then observed under a fluorescence microscope (Zeiss Axio Observer Z1, Carl Zeiss AG, Oberkochen, Germany).

### Western blot analysis

Cells were treated with LL6, linsitinib, or dasatinib for the indicated time intervals. Before harvesting, the cells were stimulated with 10% FBS for 20 min. Total cell lysates were prepared with modified radioimmunoprecipitation assay lysis buffer (50 mM Tris-HCl [pH 7.4], 150 mM NaCl, 1 mM EDTA, 0.25% sodium deoxycholate, 1% Triton X-100, 100 mM NaF, 5 mM Na_3_VO_4_, 1 mM PMSF, 1 μg/mL aprotinin, 1 μg/mL leupeptin, and 1 μg/mL pepstatin). Equal amounts of protein (20 μg) were subjected to 8% SDS-PAGE and electrically transferred onto polyvinylidene difluoride membranes (Bio-Rad Laboratories, Hercules, CA, USA). Membranes were blocked with blocking buffer (3% BSA in Tris-buffered saline containing 0.1% Tween-20 [TBST]) for 1 h at room temperature. The membranes were incubated with primary antibodies diluted in 3% BSA in TBST (1:1,000) overnight at 4 °C, washed multiple times with TBST, and incubated with secondary antibodies diluted in 3% nonfat dry milk in TBST (1:5,000) for 1 h at room temperature. The membranes were washed multiple times with TBST and visualized using an enhanced chemiluminescence detection kit (Thermo Fisher Scientific). Densitometric analysis was performed using ImageJ software.

### Targeted sequencing for analysis of EGFR mutation status

To extract genomic DNA from PC9 and PC9/ER cells, cells were treated with a lysis buffer (150 mM Tris-HCl [pH 8.5], 200 mM NaCl, 5 mM EDTA [pH 8.0], 0.2% SDS, and 300 μg/mL proteinase K) and subsequently incubated at 55 °C overnight. DNA was extracted using isopropanol as reported previously 43, further purified with PCI buffer (phenol : chloroform : isoamyl alcohol, 25:24:1), washed with 70% ethanol, and dissolved in TE buffer. For target capture, DNA was sheared into approximately 180 bp fragments, end-repaired, dA-tailed, and adapter-ligated using Illumina adapter pairs. Hybridization probes (Celemics, Seoul, Republic of Korea) were mixed with the target DNA and then separated by streptavidin beads. Target capture libraries were sequenced with the Nextseq500 platform (Illumina, San Diego, USA) using 2 × 150 bp paired-end run. The BWA aligner was used to map the sequence reads genome. Local alignment and duplication removal were performed with the Genome Analysis Tool Kit (GATK, Broad Institute, Cambridge, MA, USA) and the Picard software (Broad Institute). VarScan were used to call SNVs, and BWA aligner was performed with the Indel Detector in GATK. Mutation candidates at various loci were annotated with the ANNOVAR tool 44.

### Animal experiments

All animal experiments were performed according to protocols approved by the Seoul National University Institutional Animal Care and Use Committee. Mice were fed standard mouse chow and water ad libitum and housed in temperature- and humidity-controlled facilities with a 12-h light/12-h dark cycle. For xenograft experiments, H1944 and A549 cells (1×10^6^ cells/spot, diluted in equal amounts of PBS and Matrigel) were subcutaneously injected into the right flank of 6-week-old non-obese diabetic-severe combined immunodeficiency (NOD/SCID) mice. After the tumor volume reached 50-150 mm^3^, the mice were randomly grouped and treated with vehicle [10% DMSO in 60% polyethylene glycol (PEG) 400 solution] or LL6 (80 mg/kg) 6 days per week for 2 weeks. Tumor growth was determined by measuring the short and long diameters of the tumor with a caliper, and body weight was measured once or twice per week to monitor toxicity. Additionally, to evaluate the effect of LL6 on mutant *Kras*-driven lung tumorigenesis, 2-month-old Kras^G12D/+^ transgenic mice 48 were randomized and treated with vehicle or LL6 (80 mg/kg) for 8 weeks. The mice were euthanized, and tumor formation was evaluated and compared with that of the vehicle-treated control group. Microscopic evaluations of the H&E-stained lung tissue were also performed to measure mean tumor number (N) and volume (V) in a blinded fashion. The number and size of tumors were calculated in five sections uniformly distributed throughout each lung. In both animal experiments, the tumor volume and burden of each sample were calculated using the following formulas: Tumor volume (mm^3^) = (short diameter)^2^ × (long diameter) × 0.5; Tumor burden (mm^3^) = number of tumors × the average of tumor volume.

We used the IVIS-Spectrum microCT and Living Image (ver. 4.2) software (PerkinElmer, Alameda, CA, USA) for monitoring metastatic tumor formation in the lungs. The instrument was operated according to the manufacturer's instruction. To facilitate the detection of photons emitted from metastatic lung tumors, we performed *ex vivo* imaging analysis. In brief, mice were injected at 60 mg/kg with the 15 mg/mL stock of luciferin prior to anesthesia. After 10-15 min, mice were euthanized, and lung tissues were excised and placed into a 60 mm dish. Tissues were immediately imaged after exposure for 1 min. Regions of interest (ROIs) from displayed images were quantified as photons/second (ph/s) using the Living Image software (PerkinElmer).

### Toxicity test

FVB mice were treated with vehicle, LL6 (20, 40, and 80 mg/kg, dissolved in 10% DMSO in 60% PEG 400 solution) or combination of linsitinib (25 mg/kg, dissolved in a 25 mM tartaric acid solution) and dasatinib (20 mg/kg, dissolved in an 80 mM citric acid solution) daily for 12 days. Blood was collected from euthanized mice under isoflurane-induced deep anesthesia by cardiac puncture. After allowing blood coagulation at 4 °C, serum was collected by centrifugation at 3,000 rpm for 10 min at 4 °C. Analysis of the serum levels of alanine aminotransferase (ALT) and aspartate aminotransferase (AST) was performed using a veterinary hematology analyzer (Fuji DRI-Chem 3500s, Fujifilm, Tokyo, Japan) according to the manufacturer' s protocols.

### Glucose tolerance test

Glucose tolerance tests were performed as described in the previously published literature 49.

### Application to a Pharmacokinetic study in rats

The pharmacokinetic properties of LL6 were investigated in SD rats (*n* = 3). The left femoral artery and vein were catheterized with polyethylene tube (Intramedic^TM^ PE-50; Becton-Dickinson Diagnostics, MD, USA) under Zoletil (Virbac, Carros, France) anesthesia (50 mg/kg, intramuscular injection). LL6 was dissolved in DMSO/PEG 400/normal saline mixture (35:35:30, v/v/v) and single dose solution (1 mg/kg) was administered to rat intravenously. Approximately 150 µL of blood samples were collected via femoral artery at predetermined time (1, 3, 5, 10, 15, 30, 60, 120 and 180 min) and equivalent volume of normal saline solution with 20 U/mL of heparin was replenished to prevent blood coagulation. After centrifugation of the sample at 16,000 × *g* for 5 min, 50 µL aliquot of plasma samples were stored at -20 °C until HPLC-MS/MS analysis. The Pharmacokinetic parameters, including terminal half-life (T_½_), area under the plasma concentration-time curve from time zero to time last (AUC_last_), area under the plasma concentration-time curve from time zero to time infinity (AUC_inf_), volume of distribution at steady-state (V_ss_) and mean residence time (MRT) were calculated using non-compartmental analysis (WinNonlin, version 3.1, NCA 201; Pharsight, Mountain View, CA, USA).

### Immunohistochemistry

Sections derived from formalin-fixed and paraffin-embedded murine lung tissues were deparaffinized by incubation overnight at 65 °C, followed by rehydration in sequential xylene and ethanol rinses. After incubation with hydrogen peroxide, the slides were washed with PBS and then incubated with 0.3% Triton X-100. The sections were incubated with blocking solution (Dako Protein Block, Dako, Glostrup, Denmark) for 30 min at room temperature after washing with PBS. The sections were further incubated with primary antibodies (pAXL, pIGF-1R, and pSrc, diluted at 1:200) overnight at 4 °C, washed with PBS several times, incubated with the corresponding biotinylated secondary antibodies (diluted at 1:500), and then washed with PBS multiple times. After adding avidin-biotin complexes (Vector Laboratories, Burlingame, CA, USA), the sections were visualized using diaminobenzidine detection reagent (Enzo Life Sciences, Farmingdale, NY, USA) and mounted with a mounting solution (Vector Laboratories).

### Statistical analysis

Data are presented as mean ± SD. All *in vitro* experiments were independently performed at least twice, and a representative result is presented. The data were calculated or analyzed using Microsoft Excel software (Microsoft Corp., Redmond, MA, USA). The IC_50_ values were determined by nonlinear regression analysis using GraphPad Prism 8 (GraphPad Software, Inc., La Jolla, CA, USA). Statistical significance was determined using a two-tailed Student's t-test or one-way analysis of variance (ANOVA) using GraphPad Prism 8. An F-test for equality of variances was performed to ensure the same variance of two test groups. The Shapiro-Wilk test was performed to determine whether the *in vitro* or *in vivo* data follows a normal distribution. A *P*-value < 0.05 was considered statistically significant.

## Results

### Synthesis of LL6 as a novel SMKI targeting IGF-1R, Src, and AXL

**Figure 1:**
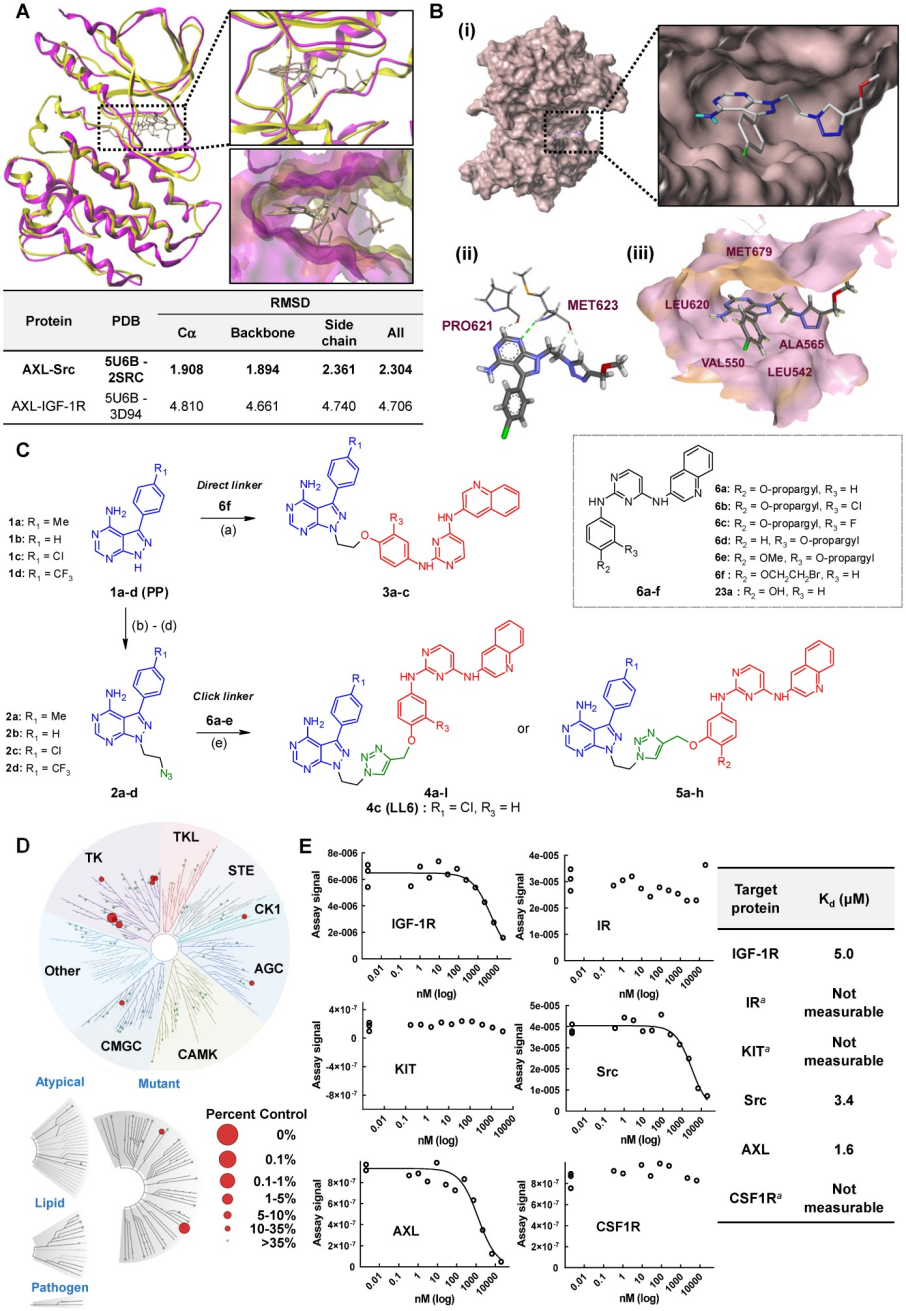
** Synthesis of LL6 and determination of its cellular targets.** (**A**) Superposed X-ray structures of AXL (magenta, PDB: 5U6B) and Src (yellow, PDB: 2SRC). Close-up views of overlaid binding pockets in cartoon (top) and surface (down) models are presented with the ligands shown in stick. (**B**) (i) Docking pose of **PP-5** in the binding pocket of AXL. A close-up view of the binding orientation of** PP-5** is shown in the box. See **Figure S1** for further details. (ii) Suggested hydrogen bonding interactions of the critical binding site residues of AXL with **PP-5**; dashed green line and light green line represent conventional hydrogen bonds and carbon hydrogen bonds, respectively. (iii) Residues with hydrophobic interactions with **PP-5** in the binding site of AXL. (**C**) Synthesis of compounds **3a-c, 4a-l,** and **5a-h**. Reagents and conditions: a) **6f**, Cs_2_CO_3_, DMF, 80ºC, 3 h, 36-48%; b) 2-bromoethanol, Cs_2_CO_3_, DMF, 80ºC, 3 h, 50-63%; c) MsCl, pyridine, rt, 5 h, 45-76%; d) NaN_3_, DMF, 60°C, 8 h, 80-88%; e) **6a-e**, CuSO_4_·5H_2_O, NaAsc, tBuOH/H_2_O/DMF, 80ºC, 3 h, 39-80%. See Table 1 for structural details. (**D**) Human kinome profile screened for LL6 at 10 µM. Red circles denote > 65% inhibition with a S-score of 0.12. The image was generated using TREE*spot*™ analysis from KINOME*scan* (DiscoveRx). (**E**) Binding titrations for IGF-1R, Src, KIT, IR, AXL, and CSF1R were performed by competition binding assays from DiscoveRx. ^a^Dose dependent inhibition curve was not obtained with these three proteins (IR, KIT, and CSF1R).

We assessed the structural similarity between AXL and Src or IGF-1R by comparing the X-ray structures of Src, IGF-1R, and AXL (pdb: 2SRC, 3D94, and 5U6B, respectively). Intriguingly, AXL displayed a significant structural similarity with Src; that is, the microenvironments of ATP-binding pockets in AXL and Src were extremely similar and the RMSD value from AXL and Src alignment was markedly lower compared with that from AXL and IGF-1R alignment (2.304 vs 4.706, **Figure 1A**). In addition, ATP-binding pockets of AXL and Src are well overlaid, as depicted in the enlarged Figures. To assess the potential of PP to bind to AXL, we performed molecular docking simulations and examined the docking poses of the linker-attached PPs bound to AXL (**Figure 1B**). Results from in silico docking studies using various PP derivatives are summarized in **Figure S1**. Compound PP-5 with the extended linker possessing an ether group in addition to a triazole ring showed a significant increase in docking scores with proper orientation of the linker toward the surface side of AXL (pdb: 5U6B) [**Figure 1B-(i)**]. Docking with the Src protein (pdb: 2SRC) showed a similar trend (**Figure S1C-D**), suggesting that the PP module conjugated with the linker in PP-5 binds to AXL. In addition, our results suggested that the reported key interactions of the original ligand in the hinge region of the AXL shown in the X-ray crystal structure (pdb: 5U6B) were retained with PP-5. Particularly, hydrogen bonding interactions of PP-5 with Pro621 and Met623 in the hinge region of AXL [**Figure 1B**-**(ii)**] along with several hydrophobic interactions [**Figure 1B**-**(iii)**] in the binding pocket indicated that PP-5 may also bind to AXL. To discover new compounds that potently inhibit IGF-1R, Src, and AXL, we employed PP as a privileged scaffold directed to Src and AXL and I2 to IGF-1R. These two modules were conjugated via copper(I)-catalyzed alkyne-azide cycloaddition (CuAAC) (**Figure 1C**). Details of the synthesis and characterization of the synthesized compounds are given in Appendix of Supplementary Materials. Various functional groups (R_1_, R_2_, and R_3_) were introduced to **PP** and the phenyl ring of **I2** to assess their anticancer activities. The key intermediates **1a-d** were *N*-alkylated to afford compounds **3a-c**, which possess a direct ether linkage with **6f**. *N*-alkylation of **1a-d** with 2-bromoethanol and mesylation, followed by the substitution reaction with sodium azide, provided** 2a-d**. CuAAC between alkyne-containing **6a-e** and various azide-containing pyrazolopyrimidines **2a-d** afforded **4a-l** and** 5a-h** with a triazole linker at different positions on the phenyl group (R_2_ and R_3_) of the IGF-1R module (**6a-e**) (**Figure 1C** and** Table 1**).

To assess the preliminary anticancer activity, the synthesized compounds were tested using the MTT assay for growth inhibition against human NSCLC (A549) cells (**Table 1** and**Figure S2**). Bosutinib was also included in the assay as a control. To identify whether dual-targeting compounds would be better than a single targeting molecule, two key intermediates **1a** and **23a** for Src and IGF-1R, respectively, were also examined using the MTT assay (**Table 1** and**Figure S2**) as control compounds. Several active compounds were selected to determine IC_50_ values against the viability of A549 cells (**Table S2**). Among the compounds, we selected **4c** (LL6 hereafter) based on its highest potency in A549 cells (**Table 1**).

The structure-activity relationship (SAR) analyses based on our depicted data are as follows. Three compounds with a direct ether linker **3a-c** did not show any inhibition on the viability of A549 cells at 10 μM (**Table 1**), indicating that the linker length between the two modules was extremely short. In contrast, several compounds with the triazole linker (**4b-d**, **4l**, and **5b-c**) displayed significant anticancer activity, with IC_50_ values shown in **Table S2**. Moreover, **4b-d** with the linker attached at the para position showed greater potency in A549 cells than the derivatives linked at the meta position (**5b-d**), as indicated by the structural analysis on the previously reported X-ray structure of IGF-1R (pdb: 3QQU) shown in **Figure S3A**-**B**. Changes made at the aminophenol ring to include chlorine, fluorine, and methoxy groups (R_2_ or R_3_) in triazole-linked compounds (**4e-l** and** 5e-h**) resulted in significant loss of potency, implying that the large substituents adjacent to the linking position caused significant steric clashes upon binding to target proteins.

Next, we assessed the kinase selectivity of LL6, the most potent among the synthesized compounds, by kinome-wide inhibition profiling. Among the 97 tested kinases (DiscoveRx), several kinases showed > 75% inhibition by LL6 treatment (**Table S3**). In the TREE*spot* visualization map of the kinome selectivity profile, the target proteins of LL6 are shown in red circles and mainly distributed in the TK family of the phylogenetic tree (**Figure 1D**). To date, most kinases targeted by clinically approved drugs are located in the TK family 45, 46. KIT, Src, IGF-1R, AXL, IR, CSF1R, and PDGFRB belong to the TK family and show > 65% inhibition with LL6 (**Figure 1D** and **Table S3**). Based on the hierarchical clustering analysis of the human kinome dataset, PDGFR, KIT, and CSF1R are found to be promiscuous kinases 47. Indeed, further validation of *K*_d_ values using an equilibrium binding titration method ruled out the possibility of LL6 binding to KIT, IR, and CSF1R, as they did not show dose-dependent inhibition curves and were found to be false positives in the assay (**Figure 1E**). These results are in accordance with the notion that some kinases are promiscuous. LL6 exhibited binding affinity to IGF-1R (*K*_d_ = 5 μM), Src (*K*_d_ = 3.4 μM), and AXL (*K*_d_ = 1.6 μM). It is worth noting that LL6 possessed weak binding affinity to IR (*K*_d_ : not accurately measurable as indicated in **Figure 1E**), conferring selectivity toward IGF-1R, although both proteins are structurally related. An opposite orientation of the **I2** ligand was suggested in the docking study with IR (**Figure S3C-D**). Note that the measured *K*_d_ values are comparable to IC_50_ values obtained from cellular assays shown in **Table S2**.

### LL6 inhibits the viability and colony-forming ability of NSCLC cells by inducing apoptosis

**Figure 2:**
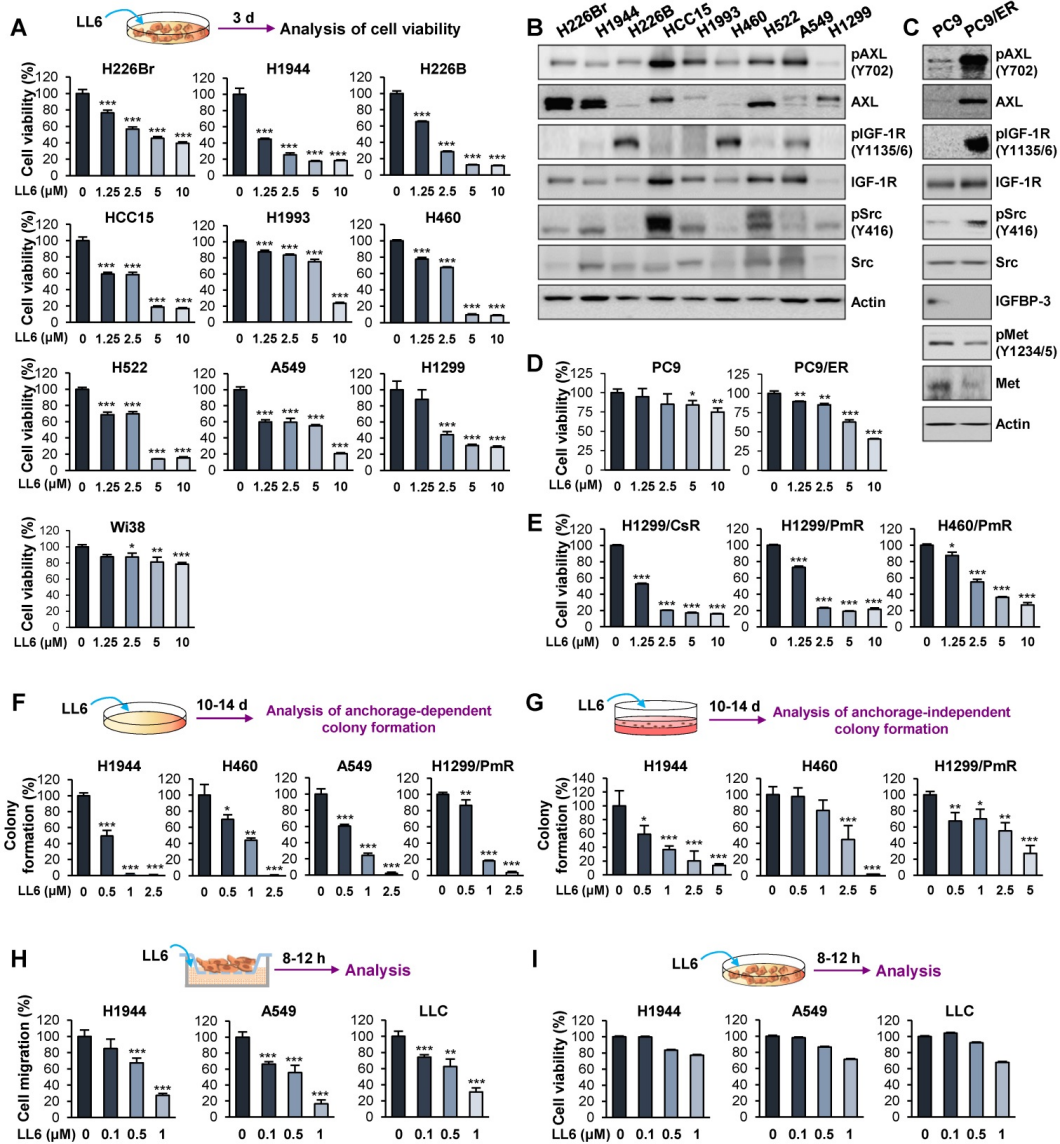
** Effects of LL6 on the viability, colony formation, and migration of several NSCLC cells.** (**A, D, E-G**) Dose-dependent inhibitory effects of LL6 on the viability of naïve (**A**), erlotinib-resistant (**D**), and chemoresistant (**E**) NSCLC cells and NSCLC colony formation grown in anchorage-dependent (**F**) and anchorage-independent (**G**) culture conditions evaluated using the MTT assay (**A, D, E**), anchorage-dependent colony formation assay (**F**), and soft agar colony formation assay (**G**), respectively. For cell viability assay, cells were treated with various concentrations of LL6 diluted in 10% FBS- (**A, E**) or 5% FBS (**D**)-containing media for three (**A, E**) or two (**D**) days. (**B, C**) Western blot analysis showing basal expression of total and phosphorylated forms of AXL, IGF-1R, and Src in various NSCLC cell lines (**B**), PC9 (**C**), and erlotinib-resistant PC9 (PC9/ER) cells (**C**). (**H**) The effect of LL6 on the migration of NSCLC cells evaluated using Transwell migration assay. (**I**) The effect of LL6 on the viability of NSCLC cells under the same experimental conditions used for cell migration determined using the MTT assay. Bars represent mean ± SD. *P* < 0.05, **P < 0.01 and ***P < 0.001, as determined using the two-tailed Student's *t*-test compared with the vehicle-treated control.

We evaluated the *in vitro* antitumor effects of LL6 against various NSCLC cell lines. LL6 displayed dose-dependent inhibitory effects on the viability of NSCLC cells with various genetic backgrounds and histologies with IC_50_ values of approximately 2.5 μM (**Figure 2A** and**Table S4**), whereas LL6 exhibited significantly less cytotoxicity in Wi38 cells, human normal diploid lung fibroblasts, than in the NSCLC cell lines used; the percentage of cell viability at the concentration of 10 μM was above 60%. The concentration of 10 μM was less than the theoretical initial plasma concentration of LL6 [22.7 μg/mL (33.3 μM)] determined by a pharmacokinetic study in rats after intravenous administration of 1 mg/mL LL6 (**Table S5** and**Figure S4**), indicating the effectiveness of LL6 even at the concentration lower than predicted plasma concentration.

We then examined the correlation between the IC_50_ values of LL6 and the expression/activity of cellular targets of LL6, IGF-1R, Src, and AXL. Western blot analysis showed the basal levels of total and phosphorylated forms of IGF-1R, Src, and AXL expression in the indicated NSCLC cells (**Figure 2B**). We observed no obvious correlation between the IC_50_ value of LL6 and the basal expression/activation levels of IGF-1R, Src, and AXL in each NSCLC cell line. These results might be due to the heterogenous activation status of IGF-1R, Src, and AXL at basal levels; only one to two kinases among IGF-1R, Src, and AXL were activated in the NSCLC cell lines used in this study. Of note, the cellular target of LL6, IGF-1R, Src, and AXL can be activated through acquired mechanism of drug resistance. Studies have shown that various cancer cells acquire resistance to IGF-1R inhibitor-based therapies, through activation of Src and AXL 12, 26. These points may explain the lack of correlation between the IC_50_ value of LL6 and the basal expression/activation levels of targets in NSCLC cells.

We next assessed the effects of LL6 on EGFR TKI (erlotinib)-resistant subline (PC9/ER) 48. Targeted next-generation sequencing analysis to determine the mutation status of EGFR in PC9/ER showed that the PC9/ER subline has various mutations in the tyrosine kinase domain (exon 18-21) of EGFR, including T790M (**Table S6**). In line with the findings, PC9/ER cells showed resistance to the corresponding EGFR TKI after a prolonged culture in the absence of drug for more than a month (**Figure S5**). PC9/ER and PC9 cells were further analyzed for activation status of several RTKs [MET, IGF-1R, and AXL], a nonreceptor tyrosine kinase Src, and IGFBP-3, all of which have been implicated in resistance to EGFR TKIs 49, 50. We observed greater activation of IGF-1R, Src, and AXL and weaker expression of IGFBP-3 in PC9/ER than in PC9 cells (**Figure 2C**). In contrast, Met was not activated in PC9/ER cells (**Figure 2C**). More importantly, LL6 exhibited greater inhibitory effects on viability of PC9/ER than that of PC9 cells (**Figure 2D**). These results suggested that IGF-1R, Src, and AXL might act as drivers of resistance to EGFR TKIs and inhibition of these kinases by using a multikinases inhibitor such as LL6 efficiently suppresses the viability of EGFR TKI-resistant cells.

We also found that the inhibitory effects of LL6 on the chemoresistant sublines (H1299/CsR, H1299/PmR and H460/PmR) 51 were comparable with those on the corresponding parental cells (H1299 and H460) (**Figure 2E**). These findings collectively suggest that LL6 is applicable to both naïve and anticancer drug-resistant lung cancer cells. We further observed that LL6 significantly suppressed colony formation in a subset of NSCLC cells in anchorage-dependent (**Figure 2F**) and anchorage-independent (**Figure 2G**) culture conditions, which indicates cell survival and tumorigenicity, respectively 52, 53. LL6 effectively suppressed the migration of NSCLC cells in a concentration-dependent manner 8-12 h after treatment (**Figure 2H**) when the cytotoxic activity of the drug was minimal (**Figure 2I**).

### Superior *in vitro* anticancer effects of LL6 compared with the pharmacological blockade of IGF-1R, Src, and AXL in combination

**Figure 3:**
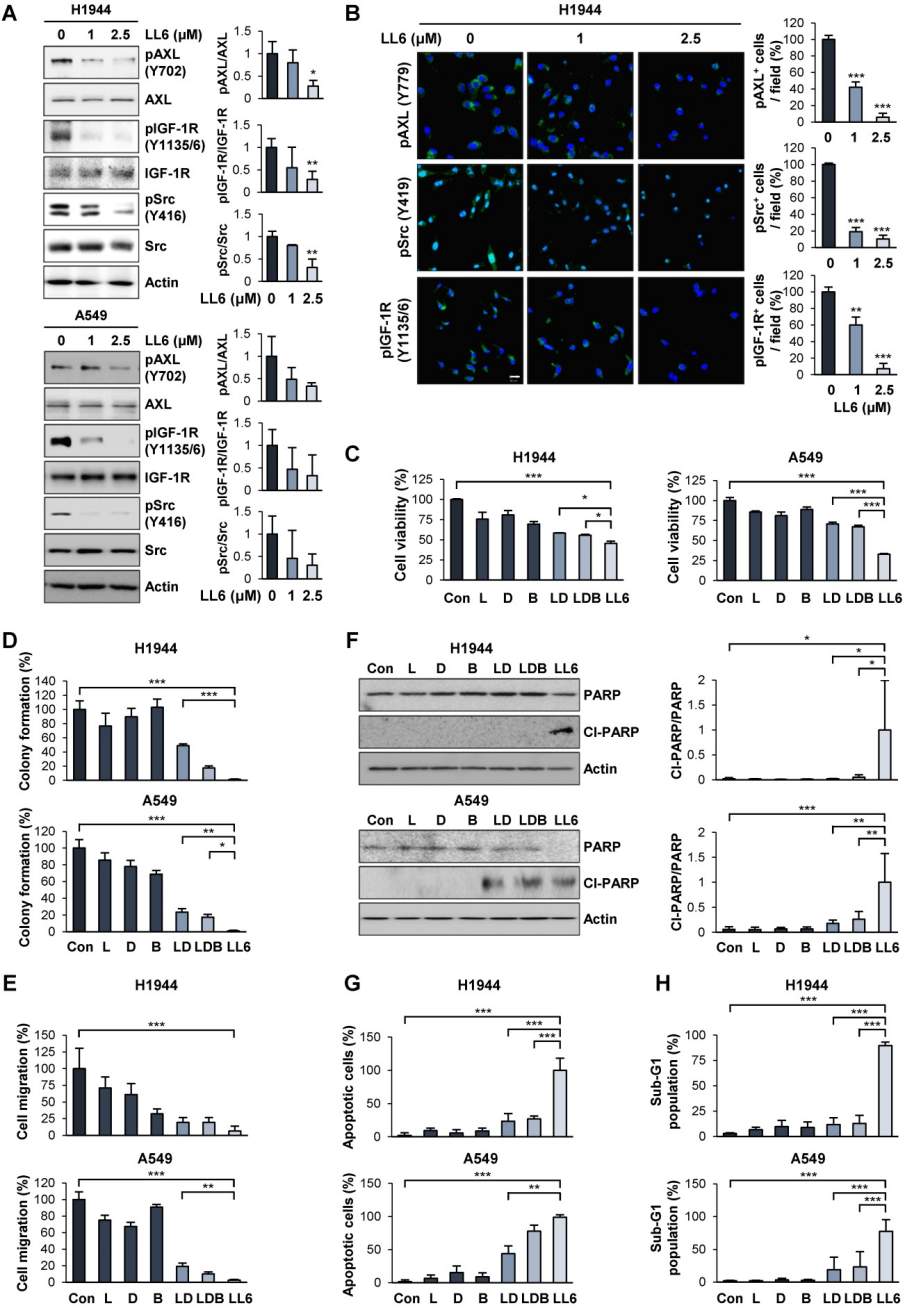
** Improved efficacy of LL6 compared with the concurrent blockade of IGF-1R, Src, and AXL by treatment with a combination of linsitinib, dasatinib, and bemcentinib.** (**A**) Inhibition of the phosphorylation of AXL, IGF-1R, and Src by treatment with LL6 was determined using Western blot analysis. (**B**) Inhibitory effects of LL6 on the phosphorylation of AXL, Src, and IGF-1R were determined using immunofluorescence analysis. (**C-E**) Inhibitory effects of LL6 (2.5 μM) on the viability (**C**), anchorage-dependent colony formation (**D**), and migration (**E**) evaluated in comparison with the treatment with a combination of linsitinib (L, 2 µM), dasatinib (D, 50 nM), or bemcentinib (B, 100 nM) (LD or LDB). (**F-H**) Proapoptotic effects of LL6 (2.5 µM), as determined by induction of PARP cleavage (**F**), chromatin condensation (**G**), and accumulation of the cell population in the sub-G1 phase (**H**), evaluated in comparison with the treatment with a combination of linsitinib (L, 2 µM), dasatinib (D, 50 nM), or bemcentinib (B, 100 nM) (LD or LDB). Bars represent mean ± SD. *P < 0.05, **P < 0.01, and ***P < 0.001, as determined using the two-tailed Student's *t*-test compared with the vehicle-treated control (A, B) and one-way ANOVA with Dunette's post-hot test (C-H). Scale bar: 20 µm.

We validated the inhibitory effect of LL6 on the activation of IGF-1R, Src, and AXL by Western blot analysis (**Figure 3A**) and immunofluorescence staining (**Figure 3B**). Because IGF-1R, Src, and AXL are potential cellular targets of LL6 in NSCLC cells, we assessed whether LL6 has comparable or superior antitumor activities compared with the concurrent blockade of IGF-1R, Src, and AXL by combined treatment with small molecular TKIs against IGF-1R (linsitinib), Src-family kinase (SFK, dasatinib), and AXL (bemcentinib). We first determined the appropriate doses of each drug that suppress their corresponding targets. Linsitinib (2 μM), dasatinib (50 nM), and bemcentinib (100 nM) as monotherapy suppressed phosphorylation of IGF-1R, Src, and AXL, respectively, in H1944 and A549 NSCLC cell lines (**Figure S6**). LL6 efficiently suppressed activation (phosphorylation) of IGF-1R, Src, and AXL at the concentration of 2.5 μM in the two cell lines (**Figure 3A**). Because the IGF-1R, Src, and AXL signaling pathways have been implicated in tumor growth and metastasis 22, 23, 54, we compared the effects of single treatment of LL6 and linsitinib (L), dasatinib (D), and bemcentinib (B) combination (LDB) on proliferation and metastatic activities of NSCLC cells *in vitro*. Compared to the LDB combination, LL6 treatment resulted in significantly greater inhibitory effects on viability (**Figure 3C**), colony-forming capacity (**Figure 3D**), and migration (**Figure 3E**) of two representative H1944 and A549 NSCLC cells.

Characteristics of apoptotic cells include membrane blebbing, chromatin condensation, and DNA fragmentation 55, 56. During apoptosis, activated caspase-3 cleaves the inhibitory proteins of caspase-activated DNases, leading to upregulation of DNase enzymatic activity and subsequent induction of internucleosomal DNA fragmentation 55. Flow cytometry analysis recognized apoptotic cells with fractional DNA contents as the cells with deficit in DNA contents (sub-G1 phase, sub-G1 peak, or hypodiploid peak) 57. Poly (ADP-ribose) polymerase (PARP) is another substrate for caspases, and the ability of PARP to repair damaged DNA is inactivated by caspase-mediated cleavage 58. Previous studies have also shown that DNA fragmentation by caspase-activated DNase and apoptosis-inducing factor induce chromatin condensation 59-61. Therefore, sub-G1 phase, PARP cleavage, and chromatin condensation can be regarded as caspase-mediated events during apoptosis execution. As sub-G1 phase is a flow cytometric indicator of apoptotic cells and PARP cleavage is an indicator of caspase activation, the level of the cells in the sub-G1 phase is positively correlated with the level of PARP cleavage. The level of the cells in the sub-G1 phase is also believed to be positively correlated with the level of chromatin condensation, a morphological phenotype of apoptotic cells. Therefore, we also determined whether the proapoptotic effects of LL6 might be superior to those of the LDB combination in terms of increases in PARP cleavage, chromatin condensation, and accumulation of cell population in the sub-G1 phase. Compared with the minimal proapoptotic effects of LDB, LL6 notably induced apoptosis, as measured by PARP cleavage (**Figure 3F**), a number of cells carried condensed chromatin in the nucleus (**Figure 3G**), and cell population accumulated in the sub-G1 phase of the cell cycle (**Figure 3H**). These results collectively suggest that the LL6-mediated combined blockade of IGF-1R, Src, and AXL may have greater improved cytotoxic, antimigratory, and proapoptotic efficacies compared with IGF-1R, Src, and AXL blockade by treatment with a combination of linsitinib, dasatinib, and bemcentinib.

### Minimal toxicity of LL6 *in vitro* and *in vivo*

**Figure 4:**
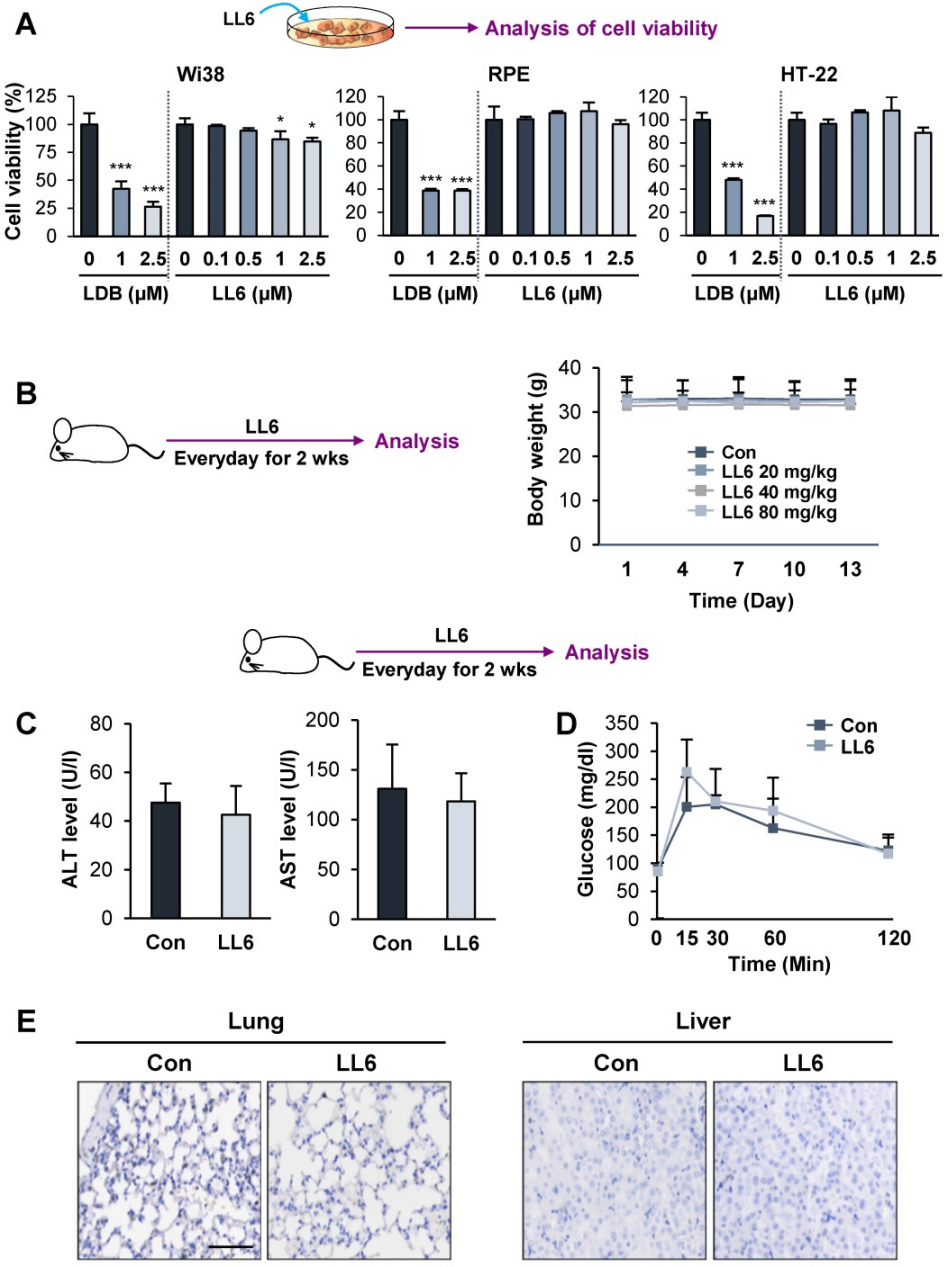
** Minimal toxicity of LL6 *in vitro* and *in vivo***. (**A**) The effects of combined treatment with linsitinib, dasatinib, and bemcentinib (LDB) or treatment with LL6 on the viability of Wi38, RPE, and HT-22 cells were determined using the MTT assay. (**B**) Body weight changes in mice treated with vehicle or LL6 (20, 40, and 80 mg/kg). (**C, D**) Changes in ALT and AST levels in the serum (**C**) and blood glucose (**D**) of the vehicle- (Con) or LL6-treated mice. (**E**) Changes in the histological features of lung and liver in the vehicle- or LL6-treated mice were determined using analysis of H&E-stained tissues. Bars represent mean ± SD. *P < 0.05, **P < 0.01, and ***P < 0.001, as determined using the two-tailed Student's *t*-test compared with the vehicle-treated control. Scale bar: 50 µm.

We then examined the toxicity profile of LL6 by comparing the effects of LL6 and LDB combination on the viability of diploid lung fibroblasts (Wi38) and two normal epithelial cells (RPE human retinal pigment epithelial cells and HT-22 mouse hippocampus cells). We observed that LL6 exhibited markedly less cytotoxicity in these normal cells compared with the LDB combination (**Figure 4A**). We further evaluated the toxicity profiles of LL6 *in vivo*. To this end, mice were treated with LL6 (20, 40, and 80 mg/kg) daily for 2 weeks, and changes in body weight were monitored. Our pharmacokinetic study suggested poor oral bioavailability of LL6 due to its poor water solubility and rapid elimination in plasma (**Table S5** and **Figure S4**). Previous reports showed that drug administration by oral gavage required more than 10-fold higher dose compared to that by intravenous administration to achieve similar plasma concentration 62-64. Given the differences in the route of administration (intravenous injection vs oral gavage), the species (rat vs mouse), and poor oral bioavailability of LL6 as predicted by the pharmacokinetic study, we decided the dose of LL6 (80 mg/kg) for oral administration in mice. As shown in **Figure 4B**, the body weight of mice treated with LL6 up to 80 mg/kg remained unchanged. Compared with the vehicle-treated control mice, the levels of alanine aminotransferase (ALT) and aspartate aminotransferase (AST) (markers of liver function) in the LL6-treated mice remained unchanged (**Figure 4C**). The blockade of IR by linsitinib treatment markedly delayed glucose clearance in mice 54. In line with weak binding affinity of LL6 on IR (**Figure 1E**)**,** the LL6-administered mice displayed minimal difference in glucose clearance compared to vehicle-treated mice (**Figure 4D**). We also observed that LL6 treatment did not cause detectable histological alterations in major organs, including the lung and liver (**Figure 4E**). These results suggested that co-targeting IGF-1R, Src, and AXL by LL6 may have greater antitumor activities with less cytotoxicity compared with that LDB-mediated IGF-1R/Src/AXL blockade. These results indicated minimal toxicity of LL6 *in vitro* and *in vivo*.

### Inhibition of mutant Kras-driven lung tumorigenesis by administration of LL6

**Figure 5:**
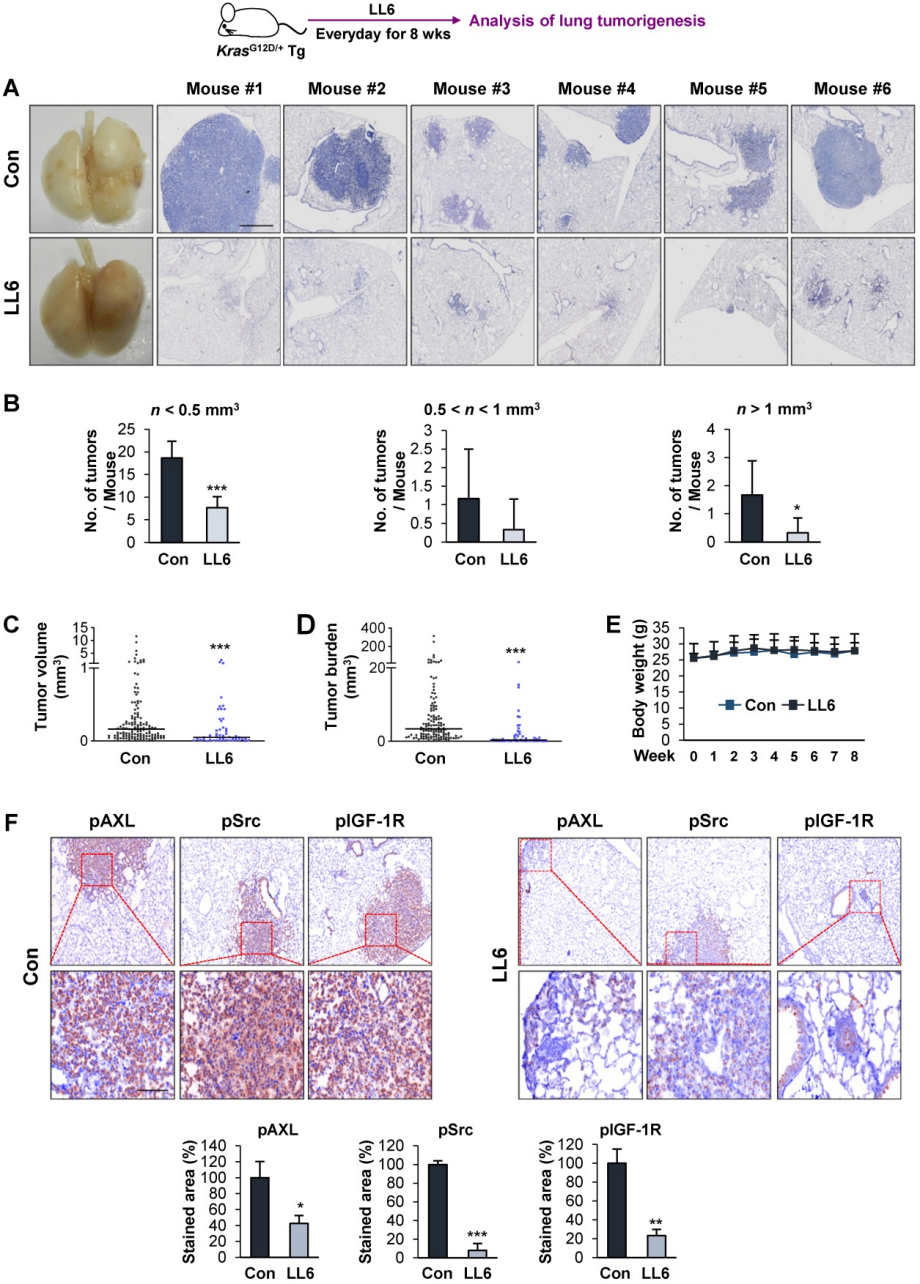
**Inhibitory effect of LL6 on *Kras*^G12D/+^-driven murine lung tumorigenesis.** (**A**) Representative images showing inhibition of lung tumor formation in the lungs of LL6 (80 mg/kg)-treated mice. *Left*. Representative photographs of the lungs from vehicle- or LL6-treated mice. *Right*. Representative photographs of H&E-stained lung sections derived from vehicle- or LL6-treated mice. (**B-D**) Microscopic analysis of the H&E-stained lung sections for lung tumor multiplicity (**B**), tumor volume (**C**), and tumor burden (**D**). (**E**) Changes in body weight of vehicle- or LL6-treated mice. (**F**) The tumoral expression of phosphorylated AXL, Src, and IGF-1R (pAXL, pSrc, and pIGF-1R) in the lungs of vehicle- or LL6-treated mice was analyzed using immunohistochemistry. *Bottom*. Quantification of the positive cells for each marker per field of view. Bars represent mean ± SD. *P < 0.05, **P < 0.01, and ***P < 0.001, as determined using the two-tailed Student's *t*-test compared with the vehicle-treated control. Scale bars: 50 µm.

We further evaluated the antitumor and antimetastatic effects of LL6 *in vivo*. To this end, we first explored the effects of LL6 on the growth of lung tumors in transgenic mice carrying mutant *Kras* (*Kras*^G12D/+^) that spontaneously develops lung tumors with a 100% incidence 65 (**Figure 5A**). Mice were randomly grouped and received LL6 (80 mg/kg) for 8 weeks. Postmortem analysis of the mice showed that LL6-treated mice had significantly decreased lung tumor growth (**Figure 5A**). Microscopic analysis of hematoxylin and eosin (H&E)-stained lung tissues further showed that the LL6-administered mice had significantly decreased lung tumor nodules, especially those > 1 mm^3^, compared with vehicle-treated mice (**Figure 5B**). Substantial decreases in tumor volume (**Figure 5C**) and burden (**Figure 5D**) were also found in LL6-treated mice. These data collectively indicate the inhibitory effect of LL6 on mutant *Kras*-mediated lung tumorigenesis in mice. Notably, body weight of vehicle- and LL6-treated mice showed no detectable difference (**Figure 5E**). We also confirmed significantly decreased levels of pIGF-1R, pSrc, and pAXL in lung tumors derived from LL6-treated mice compared with those from vehicle-treated mice (**Figure 5F**). These results suggested that targeting AXL, Src, and IGF-1R by LL6 may result in a significant suppression in mutant* Kras*-driven lung tumor growth.

### Effects of LL6 on the growth and metastatic activities of NSCLC cells with limited toxicity *in vivo*

**Figure 6:**
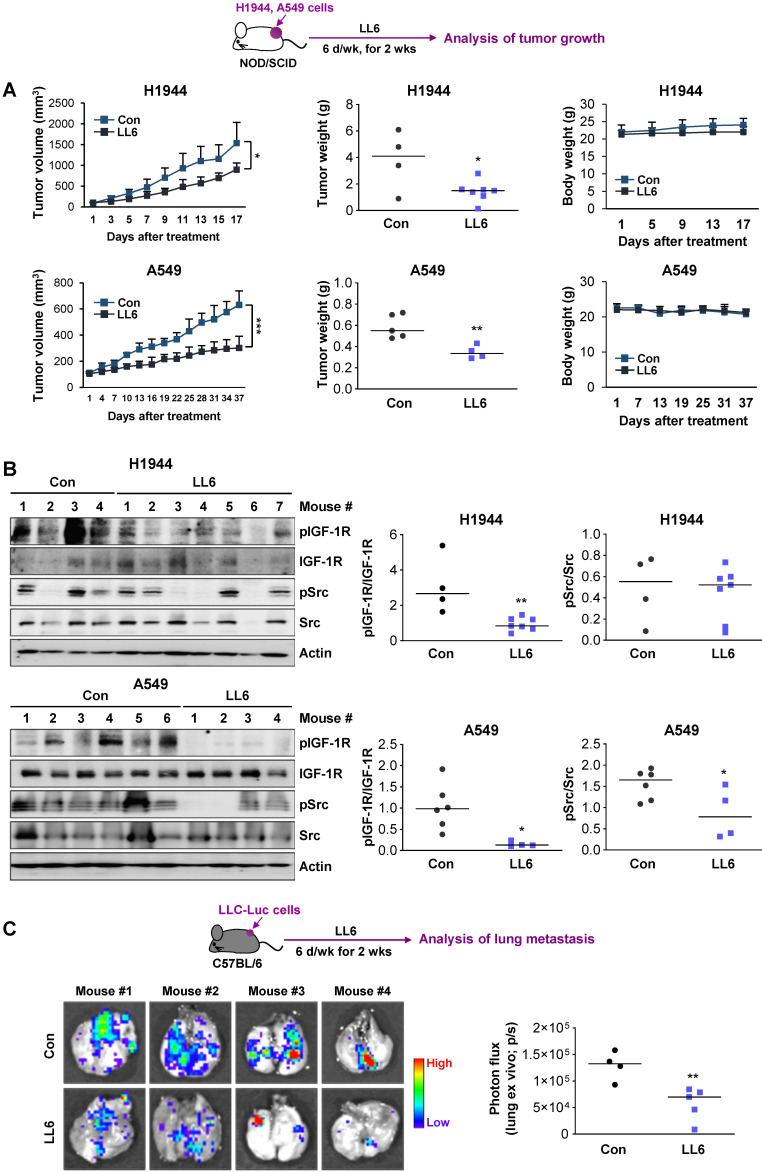
** Inhibition of the growth of xenograft tumors and spontaneous lung metastasis of LLC allografts by administration of LL6 *in vivo*.** (**A**) Inhibition of tumor growth (left) and tumor weight at endpoint (middle) by treatment with LL6 (80 mg/kg) with negligible changes in body weight (right). (**B**) Suppression of the activation of IGF-1R and Src in tumors from mice treated with LL6. *Right*. Quantification of the level of phosphorylated IGF-1R or Src expression versus corresponding total protein expression by densitometric analysis using ImageJ software. (**C**) Significant reduction in metastatic tumor formation in the lungs after treatment with LL6 (80 mg/kg). Bioluminescence imaging shows a decrease in tumor formation in the lungs of LL6-treated mice. Bars represent mean ± SD. *P* < 0.05 and *P* < 0.01, as determined using the two-tailed Student's *t*-test compared with the vehicle-treated control.

We evaluated the effects of LL6 on the tumor growth using xenograft tumors of human NSCLC cell lines (H1944 and A549). Compared with vehicle-treated mice, the LL6-administered mice showed significantly reduced tumor growth and weight with minimal changes in body weight (**Figure 6A**). Western blot analysis of the tumors further showed that the expression of phosphorylated IGF-1R and Src was substantially suppressed in tumors derived from the LL6-treated tumors (**Figure 6B**). LL6 showed quite variable impact on IGF-1R and Src activation in xenograft tumor models, which might be due to the tumor heterogeneity. A subpopulation of slow-cycling cancer cells, characterized by lack of the proliferation marker Ki67 expression and chemoresistance 51, 66, 67, has been found even in rapidly growing tumors and cancer cell lines. Hence, growth rate of subpopulations in the tumors in different mice might have impacted on drug sensitivity. To examine the effects of LL6 on the metastasis of lung cancer cells, C57BL/6 mice bearing subcutaneous LLC allografts 68 were orally treated with vehicle or LL6 daily for 2 weeks (**Figure 6C**). Compared with control lungs from vehicle-treated mice, lungs from LL6-treated mice showed significantly decreased metastatic tumor nodules (**Figure 6C**). Taken together, these results suggest the antitumor and antimetastatic activities of LL6 without overt toxicity *in vivo*.

## Discussion

Numerous studies have demonstrated the plasticity of cancer cells, which mediate anticancer drug resistance through activation of compensatory pathways once specific pathways are blocked. Hence, a paradigm in drug discovery is shifting from one drug-one target to one drug-multiple target model 69, 70. Based on previous studies supporting the function of Src and AXL in resistance to IGF-1R inhibitors 12, 14, 26, we attempted to develop efficacious SMKIs targeting IGF-1R, Src, and AXL. Herein, we report a novel multitarget SMKI, LL6, which effectively inactivates these kinases and has potent anticancer activities with outstanding safety profiles both *in vitro* and *in vivo*. These results provide preclinical evidence for the use of LL6 as a novel anticancer drug targeting IGF-1R, Src, and AXL without overt toxicity.

Molecularly targeted anticancer therapy has been utilized as the first-line therapy either as a single treatment or in combination with other anticancer therapeutics 11, 71. However, drug resistance through mutations in cellular targets and activation of bypass signaling pathways hamper their clinical utility 72. Hence, selection of appropriate targets after full understanding of complex signaling networks in cancer cells and development of SMKIs that simultaneously act on targets involved in bypass signaling pathways have emerged as a novel strategy for anticancer drug discovery 73-75. IGF-1R signaling has shown crucial roles in the development and progression of several types of cancer and resistance to various anticancer therapies 54. However, currently available IGF-1R-targeted therapies have shown minimal efficacy in advanced clinical trials owing to drug resistance through activation of bypass signaling mechanisms. Previous studies have shown the implication of Src and AXL signaling pathways in resistance to IGF-1R-targeted anticancer drugs 12, 14, 26. Src and AXL are frequently overexpressed in various types of human cancers, including NSCLC, and serve as poor prognosis markers in cancer patients 21-23, 76, 77. As several Src- and AXL-targeting anticancer drugs have been evaluated in clinical studies 22, 23, concurrent inhibition of IGF-1R, Src, and AXL by combinatorial treatment with each SMKI would be an effective therapeutic strategy. However, as with many other anticancer regimens, various toxicities have limited the use of Src and AXL inhibitors in the clinic 22, 23, 78. Therefore, we attempted to develop potent but safe, multitarget SMKIs that concurrently target IGF-1R, Src, and AXL as a single entity.

In this regard, our observation of the structural similarity between the ATP-binding pocket of the Src kinase and that of AXL kinase and previous studies showing the blockade of AXL activity by an Src inhibitor provided a starting point for the development of such a candidate. In fact, a recent study employed a ligand-based drug design as an approach to develop PP-based focused library to target AXL and FLT3 31. Our investigation of the syntheses of multitarget SMKIs using the PP-I2 hybrids offered structural implications for the development of such SMKIs, and further SAR analysis and in silico studies provided one lead candidate, LL6. Our findings suggested the following: 1) linker size and attachment position for conjugating two modules with different orientations played an important role in the design of potent anticancer agents, and 2) substituents on the meta position (R_3_) of the aminophenol ring of I2 are detrimental to anticancer activity because of steric clashes. The attachment of the linker on the methoxy group in the meta position resulted in loss of potency in **5a-h**, indicating that the conformation of the IGF-1R ligand (2,4-bis-arylamino-1,3-pyrimidines, I2) with the U shape, shown in the X-ray structure (pdb: 3QQU) bound to IGF-1R (**Figure S3**) 79, is retained in LL6. Although the experimentally measured binding affinities of LL6 to the target kinases have low micromolar ranges (< 5 μM), the kinome-wide selectivity of LL6 was observed. Of note, LL6 showed distinct binding affinities for IR and IGF-1R. It was previously observed that the binding pocket of IR TK is wider than that of IGF-1R 80. Our docking simulations of LL6 using the X-ray structure of IR TK (pdb: 5E1S) suggest that the aminophenol group attached to the triazole linker is buried in the hydrophobic pocket of the IR TK, displaying opposite orientations of the original ligand bound to IGF-1R. Meanwhile, removing all water molecules from the X-ray structure resulted in similar poses. Therefore, potential steric clashes when the PP module is attached to the triazole linker-attached IGF-1R ligand module (I2) might cause the observed abrogated binding affinity of LL6 to IR TK.

Our results from several biological analyses shown in the current study demonstrate that LL6 offers reasonable antitumor effects by inducing apoptosis. Clonogenicity under adherent conditions is associated with survival capacity without cell-to-cell interactions 52; thus, it is likely that LL6 suppresses disseminated cancer cells. In support of this notion, LL6 exhibited significant inhibitory effects on cancer cell migration. Considering the observed feature of LL6 to suppress IGF-1R, Src, and AXL, LL6 offered the expected results, that is, more efficacious antitumor activities compared with concurrent pharmacologic suppression of IGF-1R, Src, and AXL. Combinatorial therapies can be challenged by various drawbacks, especially enhanced side effects and toxicity. Of note, LL6 minimally affected the viability of normal cells derived from various organs *in vitro* and displayed no obvious toxicities *in vivo*, such as diabetes-like symptoms, hepatotoxicity, and histological alterations in major organs. Moreover, LL6 significantly suppressed mutant *Kras*-driven lung tumorigenesis and growth and metastatic tumor formation in a syngeneic mouse model. Although additional investigations on the efficacy and toxicity profiles of LL6 are required, these results collectively support its clinical utility as an anticancer drug.

Multifunctional therapeutic strategies using multitarget-directed ligands that act simultaneously on various biological targets have emerged as a new principle for drug discovery 73-75, especially for the treatment of cancers with heterogeneous natures. Although monoclonal antibody has a defined specificity, a dual-targeting approach with antibodies requires substantial costs for manufacturing. Moreover, bispecific antibodies cope with issues related to stability and pharmacokinetic properties 81. Compared with antibody engineering, concurrent targeting of several oncogenic pathways by multitargeting SMKIs would have a number of potential advantages: synergistic antitumor activities, reduced drug resistance, and limited toxicity may lead to a potent efficacy. Indeed, enormous efforts have been dedicated to developing rationally designed multitarget SMKIs since the FDA approval of sunitinib in 2006 82. In fact, several multitarget SMKIs have been approved for clinical use 83. Most FDA-approved SMKIs have been found to interact with more than one target 46.

Therefore, our study presents LL6 as a novel multitarget SMKI that concurrently targets IGF-1R, Src, and AXL with great potency but limited toxicity. Our strategy could be a starting point in understanding polypharmacological networks for the development of drug candidates with improved therapeutic effects. Considering the antitumor efficacy and safety of LL6, LL6 could provide a new design principle to develop novel multitarget SMKI candidates. Our study also provides the rationale to discover novel AXL inhibitors by repositioning the chemical library of potential Src inhibitors. Additional studies are warranted to validate our ligand-based target profiling approach to evaluate the efficacy, pharmacokinetic and pharmacodynamic profiles, and toxicity of LL6 in advanced preclinical and clinical settings.

## Supplementary Material

Supplementary figures.

## Figures and Tables

**Table 1:** Anti-proliferation effects of compounds **3**-**5** against A549 cancer cell line

	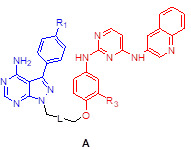			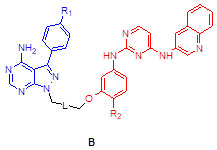	
Compound	Structure	R_1_	R_2_/R_3_	L (linker)	% cells growth at 10 μM
3a	A	Me	H		98
3b		H	H		96
3c		Cl	H		82
4a	A	Me	H	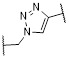	82
4b		H	H		20
4c (LL6)		Cl	H		6.4
4d		CF_3_	H		26
4e		Me	Cl		101
4f		H	Cl		103
4g		Cl	Cl		99
4h		CF_3_	Cl		27
4i		Me	F		88
4j		H	F		96
4k		Cl	F		94
4l		CF_3_	F		47
5a	B	Me	H	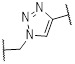	68
5b		H	H		35
5c		Cl	H		52
5d		CF_3_	H		76
5e		Me	OMe		65
5f		H	OMe		91
5g		Cl	OMe		100
5h		CF_3_	OMe		106
Bosutinib					46
1a					98
23a					69

## Abbreviations

ALT: alanine aminotransferase
AST: aspartate aminotransferase
CuAAC: copper(I)-catalyzed alkyne-azide cycloaddition
EGFR: epidermal growth factor receptor
IGF-1R: type I insulin-like growth factor receptor
IR: insulin receptor
NSCLC: non-small cell lung cancer
NOD/SCID: non-obese diabetic-severe combined immunodeficiency
PARP: poly (ADP-ribose) polymerase
RMSD: root-mean-square derivation
ROI: region of interest
SAR: structure-activity relationship
SMKI: small molecule kinase inhibitor
TK: tyrosine kinase
TKI: tyrosine kinase inhibitor
