# Supplementary material for: Development of the phenylpyrazolo[3,4-d]pyrimidine-based, insulin-like growth factor receptor/Src/AXL-targeting small molecule kinase inhibitor
Source: Theranostics. 2021 Jan 1;11(4):1918–36. doi: 10.7150/thno.48865 (PMC7778606; doi:10.7150/thno.48865)
Supplement: Supplementary file 1 — Supplementary figures. [file thnov11p1918s1.pdf]

Supplementary Materials

Development of the phenylpyrazolo[3,4-*d*]pyrimidine-based, insulin-like growth factor receptor/Src/AXL-targeting small molecule kinase inhibitor

Ho Jin Lee, Phuong Chi Pham, Honglan Pei, Bumhee Lim, Seung Yeob Hyun, Byungyeob Baek, Byungjin Kim, Yunha Kim, Min-Hwan Kim, Nae-Won Kang, Hye-Young Min, Dae-Duk Kim, Jeeyeon Lee, Ho-Young Lee

Table S1. Antibodies used in this study.

Target	Vendor	Catalogue Number	Clone	Dilution ratio	Application
pAXL (Y702)	Cell Signaling	5724	D12B2	1:1,000	Western blot (WB)
pAXL (Y779)	R&D	AF2228		1:200	Immunofluorescence (IF) Immunohistochemistry (IHC)
AXL	Santa Cruz	sc-166269	H-3	1:1,000	WB
pIGF-1R (Y1135/6)	Cell Signaling	3024	19H7	1:1,000	WB
pIGF-1R (Y1135/6)	Thermo Fisher Scientific	44-804G		1:200	IF, IHC
IGF-1R	Santa Cruz	sc-713	C-20	1:1,000	WB
pSrc (Y416)	Cell Signaling	6943	D49G4	1:1,000	WB
pSrc (Y419)	Thermo Fisher Scientific	44-660G		1:200	IF, IHC
Src	Cell Signaling	2109	36D10	1:1,000	WB
IGFBP-3	Santa Cruz	sc-9028	H-98	1:1,000	WB
pMet (Y1234/5)	Cell Signaling	3077	D26	1:1,000	WB
Met	Cell Signaling	8198	D1C2	1:1,000	WB
Actin	Santa Cruz	sc-47778	C4	1:1,000	WB
PARP	Santa Cruz	sc-7150	H-250	1:1,000	WB
Cleaved PARP	BD	552596	F21-852	1:1,000	WB
HRP-conjugated goat anti-mouse igG	GeneTex	213111-01		1:5,000	WB
HRP-conjugated goat anti-rabbit igG	GeneTex	213110-01		1:5,000	WB
Alexa Fluor 488-conjugated goat anti-rabbit igG	Thermo Fisher Scientific	A-11034		1:1,000	IF
Biotinylated goat anti-rabbit igG	Bethyl Laboratories	A120-101B		1:1,000	IHC

Table S2. IC_{50} values of selected compounds against the viability of A549 cells.

Compound	IC_{50} (μM)
**4b**	5.77
**4c (LL6)**	2.59
**4d**	4.87
**4l**	8.24
**5b**	5.51
**5c**	7.15

Table S3. Human kinome profile screened for LL6 (4c) at 10 μM.

DiscoveRx Gene Symbol	Percent Control	DiscoveRx Gene Symbol	Percent Control
ABL1(E255K)-phosphorylated	92	KIT(D816V)	66
ABL1(T315I)-phosphorylated	100	KIT(V559D,T670I)	80
ABL1-nonphosphorylated	61	LKB1	90
ABL1-phosphorylated	62	MAP3K4	75
ACVR1B	50	MAPKAPK2	100
ADCK3	67	MARK3	100
AKT1	100	MEK1	65
AKT2	77	MEK2	95
ALK	100	MET	75
AURKA	48	MKNK1	100
AURKB	55	MKNK2	89
AXL	22	MLK1	75
BMPR2	40	p38-alpha	100
BRAF	45	p38-beta	65
BRAF(V600E)	22	PAK1	100
BTK	100	PAK2	100
CDK11	13	PAK4	97
CDK2	99	PCTK1	78
CDK3	97	PDGFRA	64
CDK7	56	PDGFRB	5
CDK9	100	PDPK1	87
CHEK1	100	PIK3C2B	100
CSF1R	29	PIK3CA	100
CSNK1D	14	PIK3CG	63
CSNK1G2	63	PIM1	98
DCAMKL1	100	PIM2	100
DYRK1B	55	PIM3	100
EGFR	82	PKAC-alpha	36
EGFR(L858R)	88	PLK1	100
EPHA2	100	PLK3	90
ERBB2	100	PLK4	66
ERBB4	40	PRKCE	100
ERK1	100	RAF1	82
FAK	73	RET	81
FGFR2	62	RIOK2	100
FGFR3	89	ROCK2	100
FLT3	43	RSK2(Kin.Dom.1-N-terminal)	25
GSK3B	67	SNARK	100
IGF1R	16	SRC	26
IKK-alpha	100	SRPK3	84
IKK-beta	100	TGFBR1	46
IR	35	TIE2	62
JAK2(JH1domain-catalytic)	100	TRKA	100
JAK3(JH1domain-catalytic)	100	TSSK1B	100
JNK1	100	TYK2(JH1domain-catalytic)	100
JNK2	67	ULK2	96
JNK3	42	VEGFR2	65
KIT	1.8	YANK3	100
		ZAP70	100

Table S4. IC_{50} values of LL6 or positive controls against the viability of NSCLC cells, those resistant to anticancer therapies, and Wi38 human lung fibroblasts.

Compound	Cell line	IC_{50} (μM)
LL6	H226Br	4.32
	H1944	1.02
	H226B	1.37
	HCC15	3.73
	H1993	7.79
	H460	3.43
	H522	3.55
	A549	6.63
	H1299	2.93
	Wi38	>10
	H1299/CsR	1.07
	H1299/PmR	1.62
	H460/PmR	3.64
	PC9	>10
	PC9/ER	8.61
Linsitinib	H1299	12.83
	H460	3.78
Dasatinib	H1299	0.02
	H460	0.54
Bemcentinib	H1299	0.53
	H460	0.18

Table S5. Pharmacokinetic parameters of LL6 after intravenous administration at a dose of 1 mg/kg in rats.

Parameter	
$T_{1/2}$ (min)	29.1 ± 10.0
AUC_{last} (ug · min/mL)	51.0 ± 3.5
AUC_{inf} (ug · min/mL)	51.1 ± 3.6
C0 (μg/mL)	22.7 ± 4.9
CL (mL/min/kg)	19.3 ± 1.2
Vss (mL/kg)	391.6 ± 186.1
MRT (min)	20.3 ± 9.6

Table S6. Targeted sequencing analysis showing EGFR mutation status in PC9 and PC9/ER cells.

Base change	Amino acid change	PC9				PC9/ER			
		Total Depth[1)]	Ref Depth[2)]	Alt Depth[3)]	VAF[4)] (%)	Total Depth	Ref Depth	Alt Depth	VAF (%)
c.27A>C	p.Ala9Ala	-	-	-	-	1,023	1,000	23	2.25
c.474C>T	p.Asn158Asn	5,645	674	4,968	88.01	9,839	690	9,147	92.97
c.1887T>A	p.Thr629Thr	4,560	4,069	491	10.77	5,344	5,002	341	6.38
c.2361G>A	p.Gln787Gln	5,565	5,060	505	9.07	10,782	10,151	625	5.80
c.2369C>T	p.Thr790Met	-	-	-	-	10,344	4,840	5,497	53.14
c.2709T>C	p.Thr903Thr	4,527	5	4,518	99.82	7,830	3	7,816	99.82

1) Total Depth: Number of reads aligned at this position
2) Ref Depth: Reference Allele Depth, Number of reads containing the reference allele
3) Alt Depth: Variant Allele Depth, Number of reads containing the variant allele
4) VAF: Variant allele frequency, Proportion of the variant allele among all alleles being considered

A

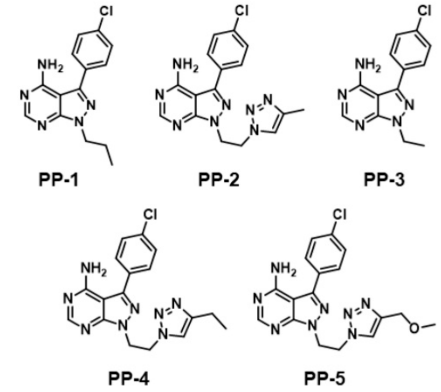

B

Molecule	Total score	Crash	Polar
**PP-5**	**6.4335**	**-1.2465**	**0.3649**
Ligand (5U6B)	5.9432	-0.7322	1.2479
PP-4	5.7674	-0.5044	1.1893
PP-1	5.7555	-0.4850	1.2981
PP-2	4.9655	-0.5309	1.1942
PP-3	4.4915	-0.5030	1.1809

C

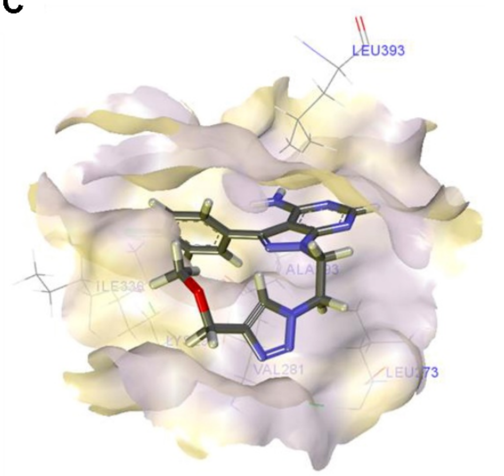

D

Molecule	Total score	Crash	Polar
**PP-5**	**10.0720**	**-0.9301**	**2.7640**
PP-2	9.3354	-0.7753	2.8747
PP-4	8.7705	-1.0144	2.5149
Ligand (2SRC)	8.7222	-0.9211	6.3905
PP-3	8.2457	-0.4354	2.4105
PP-1	7.6424	-0.4220	2.3347

Figure S1. Docking analysis of 3-phenylpyrazolo[3,4-*d*]pyrimidin-4-amine (PP) derivatives. (**A**) Structures of PP derivatives used for docking study. (**B**) Docking scores of PP derivatives compared to the original ligand used in X-ray structure of AXL (pdb: 5U6B). (**C**) Docking pose of **PP5** in the binding pocket of Src. (**D**) Docking scores of PP derivatives compared to the original ligand used in X-ray structure of (PDB:2SRC).

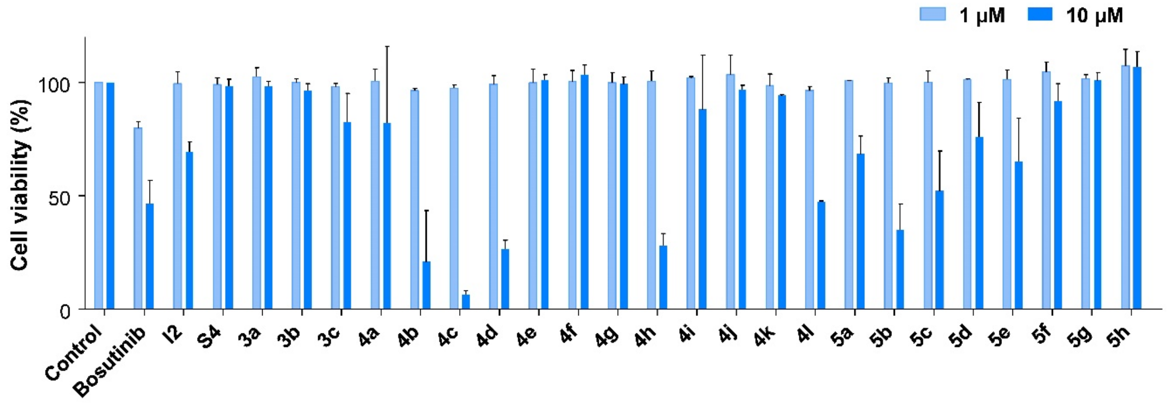

Figure S2. **Screening of compounds 3a-c, 4a-l, and 5a-h by measuring inhibitory effects on the viability of A549 by the MTT assay.**

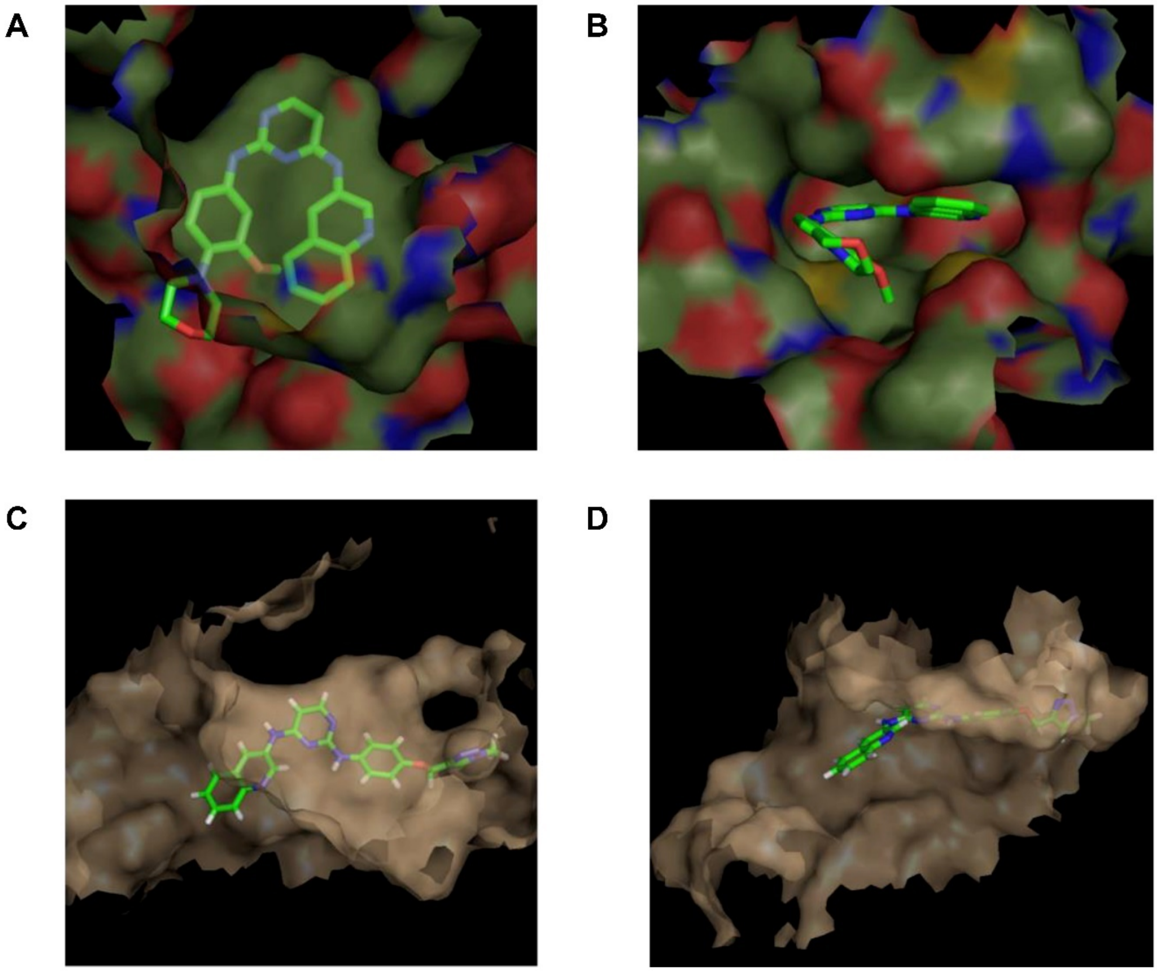

Figure S3. Docking analysis of 4-bis-arylamino-1,3-pyrimidines (I2). (**A-B**) X-ray structures of 2,4-bis-arylamino-1,3-pyrimidines (**I2**) bound to IGF-1R (pdb: 3QQU). Binding pockets from top (**A**) and front views (**B**). (**C-D**) Docking pose of triazole linker-attached **I2** module in the binding pocket of insulin receptor tyrosine kinase (pdb: 5E1S). Binding pockets from top (**C**) and front views (**D**). Note that the aminophenol group of the ligand is not pointing toward the solvent exposed surface site.

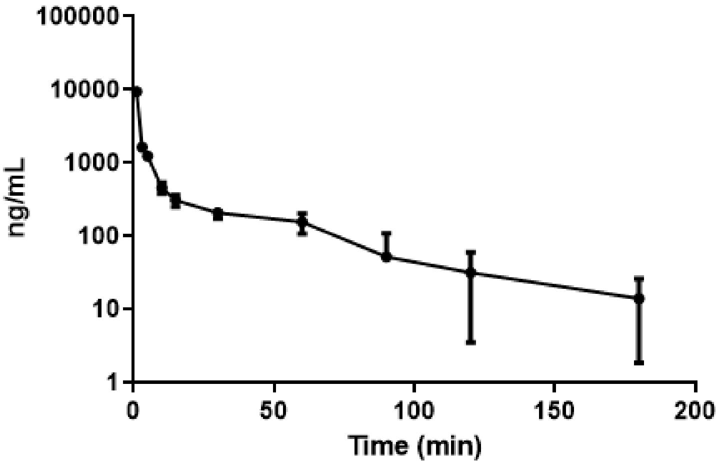

Figure S4. Plasma concentration versus time profiles of LL6 after intravenous administration at a dose of 1 mg/kg in rats.

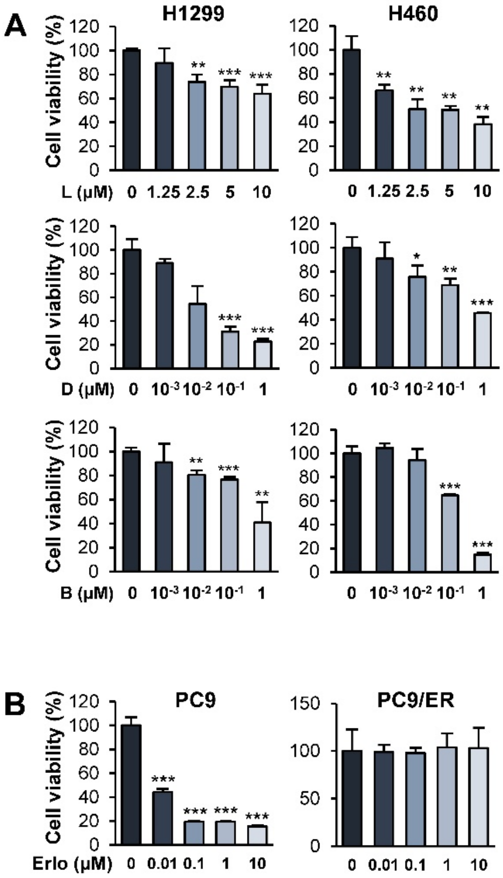

Figure S5. Dose-dependent inhibitory effect of linsitinib, dasatinib, and bemcentinib in H1299 and H460 cells and sustained resistance to erlotinib in PC9/ER cells. (**A**) H1299 and H460 cells were treated with various concentrations of linsitinib (L), dasatinib (D), and bemcentinib (B) for three days. Cell viability was determined by the MTT assay. (**B**) PC9 and PC9/ER cells, cultured in the absence of erlotinib (Erlo) for more than a month, were treated with increasing concentrations of erlotinib for three days. Cell viability was determined by the MTT assay. Bars represent mean ± SD. **P* < 0.05, ***P* < 0.01, and ****P* < 0.001, as determined using the two-tailed Student's *t*-test compared with the vehicle-treated control.

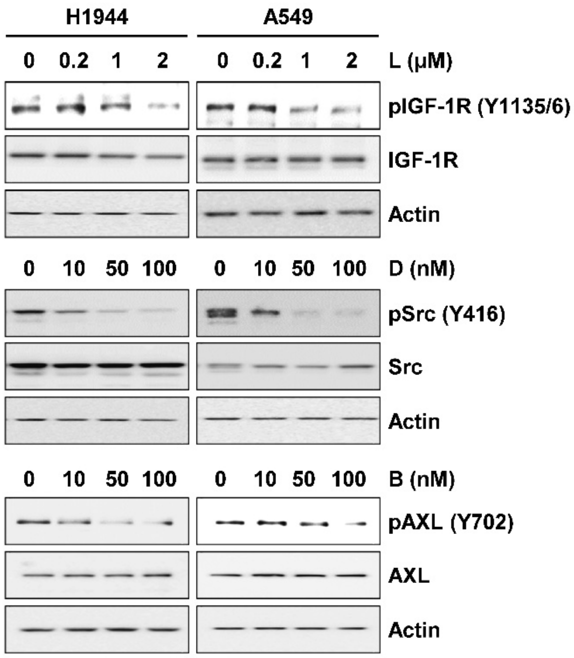

Figure S6. Inhibition of the phosphorylation of AXL, IGF-1R, and Src by treatment with linsitinib, dasatinib, and bemcentinib, respectively. H1944 and A549 cells were treated with various concentrations of linsitinib (L), dasatinib (D), and bemcentinib (B) for 1 day. Prior to harvesting, cells were further stimulated with 10% FBS for 20 min. Cell lysates were prepared, and the expression of total and phosphorylated forms of AXL, IGF-1R, and Src was determined by Western blot analysis.

Appendix

General information on synthesis, detailed synthetic procedures, and NMR spectra of screened compounds and their synthetic intermediates

Table of Contents

I. Synthesis **S2 – S12**

1. General information S2
2. Synthesis
    - 2.1 General synthetic procedure of **3** S3 – S4
    - 2.2 General synthetic procedure of **4-5** S5 – S16
3. ^{1}H- and ^{13}C-NMR spectra S17 – S39

I. Synthesis

1. General information

Unless specified, all reagents and solvents were purchased from commercial venders and used without further purification. All reactions were carried out under dry nitrogen using oven-dried glassware. Analytical thin layer chromatography on silica gel 60 F254 plate (Merck, Darmstadt, Germany) was used for monitoring the reaction under the UV light at 254 nm (VL-4.LC, Vilber Lourmat, Eberhardzell, Germany). Column chromatography was carried out on ZEOprep silica gel (230~400 mesh; Zeochem, Lake Zurich, Switzerland) with methanol, ethyl acetate and dichloromethane as eluents. ^{1}H-(400, 500, 600 and 800 MHz) and ^{13}C-NMR (100, 125, 150 and 200 MHz) spectra were collected on a JNM-ECZ400s (JEOL, Tokyo, Japan), Avance 500 (Bruker, Billerica, MA, USA) and JNM-ECA-600 (JEOL, Tokyo, Japan) at ambient temperature. All chemical shifts (δ) are quoted in parts per million (ppm) using the residual signal of solvent as an internal reference and coupling constants (*J*) are reported in hertz (Hz). The multiplicity of the signal is indicated as follows: s (singlet), d (doublet), t (triplet), q (quartet), m (muliplet), dd (doublet of doublets), bs (broad singlet). High-resolution mass spectra (HR-MS) were carried out on a JMS-700 MStation (JEOL, Tokyo, Japan) under fast atom bombardments (FAB) condition.

2. Synthesis

The procedures for the synthesis of **1**, **6**, and the control compounds for Src (**1a**) and IGF-1R (**23a**) modules used in assays are described in the previous report.(1)

2.1 General synthetic procedure of 3

To a solution of **1a** (1.0 equiv.) and **6f** (1.0 equiv.) in anhydrous DMF (2 mL) under a N_2 atmosphere was added cesium carbonate (4.0 equiv.) and the mixture was stirred at 80 °C for 3 h. The resulting mixture was partitioned between water and ethyl acetate (3 X 100 mL). The organic layer was dried with sodium sulfate and evaporated *in vacuo*. The crude product was purified using silica gel chromatography with ethyl acetate/methanol gradient.

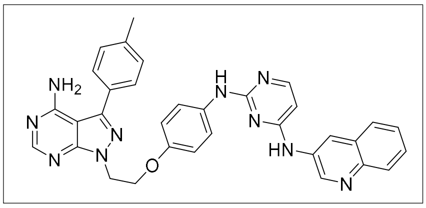

N^2-(4-(2-(4-amino-3-(p-tolyl)-1H-pyrazolo[3,4-d]pyrimidin-1-yl) ethoxy)phenyl)-N^4-(quinolin-3-yl)pyrimidine-2,4-diamine (3a) This compound was synthesized using the general procedure depicted above (section 2.1), yield 48.1%. ^{1}H NMR (600 MHz, DMSO-*d*$_6$) δ 9.82 (s, 1H), 9.09 (s, 1H), 8.92 (brs, 1H), 8.89 (s, 1H), 8.29 (s, 1H), 8.06 (d, *J* = 5.5 Hz, 1H), 7.90 (d, *J* = 8.3 Hz, 1H), 7.70 (brs, 1H), 7.57 (d, *J* = 7.8 Hz, 2H), 7.54-7.50 (m, 3H), 7.46-7.45 (m, 1H), 7.34 (d, *J* = 7.8 Hz, 2H), 6.84 (d, *J* = 9.2 Hz, 2H), 6.29 (d, *J* = 5.5 Hz, 1H), 4.74 (t, *J* = 5.5 Hz, 2H), 4.48 (t, *J* = 5.5 Hz, 2H), 2.38 (s, 3H) ppm. ^{13}C NMR (150 MHz, DMSO- *d*$_6$) δ 160.38, 159.86, 158.13, 156.46, 155.77, 154.75, 153.16, 145.02, 143.91, 143.22, 138.05, 134.00, 130.02, 129.68 (3C), 128.46, 128.11 (3C), 127.21, 126.82, 126.67, 121.86, 121.08, 114.89, 114.62 (2C), 98.73, 97.40, 66.00, 45.98, 20.86 ppm. LC-MS (ESI) *m/z* 581.00 [M + H]$^+$. HRMS (FAB) calculated for $C_{33}H_{28}N_{10}O$ [M + H]$^+$: 581.2526, found: 581.2522.

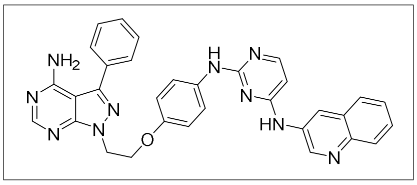

N^2-(4-(2-(4-amino-3-phenyl-1H-pyrazolo[3,4-d]pyrimidin-1-yl)ethoxy)phenyl)-N^4-(quinolin-3-yl)pyrimidine-2,4-diamine (3b) This compound was synthesized using the general procedure depicted above (section 2.1), yield 46.6%. ^{1}H NMR (600 MHz, DMSO-*d$_6$*) δ 9.80 (s, 1H), 9.08 (s, 1H), 8.92 (brs, 1H), 8.89 (s, 1H), 8.30 (s, 1H), 8.06 (d, *J* = 5.5 Hz, 1H), 7.90 (d, *J* = 8.2 Hz, 1H), 7.69-7.68 (m, 3H), 7.56-7.52 (m, 4H), 7.50-7.47 (m, 2H), 7.45 (t, *J* = 7.1 Hz, 1H), 6.85 (d, *J* = 9.2 Hz, 2H), 6.28 (d, *J* = 5.9 Hz, 1H), 4.75 (t, *J* = 5.5 Hz, 2H), 4.49 (t, *J* = 5.5 Hz, 2H) ppm. ^{13}C NMR (150 MHz, DMSO-*d$_6$*) δ 160.37, 159.86, 158.13, 156.47, 155.80, 154.80, 153.15, 145.01, 143.89, 143.21, 133.99, 132.85, 129.10 (3C), 128.63, 128.45, 128.20 (2C), 128.11, 127.20, 126.82, 126.67, 121.85, 121.06, 114.89, 114.62 (2C), 98.72, 97.41, 65.98, 46.02 ppm. LC-MS (ESI) *m/z* 567.00 [M + H]$^+$. HRMS (FAB) calculated for $C_{32}H_{26}N_{10}O$ [M + H]$^+$: 567.2369, found: 567.2365.

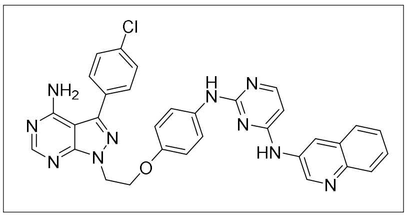

N^2-(4-(2-(4-amino-3-(4-chlorophenyl)-1H-pyrazolo[3,4-d]pyrimidin-1-yl)ethoxy)phenyl)-N^4-(quinolin-3-yl)pyrimidine-2,4-diamine (3c) This compound was synthesized using the general procedure depicted above (section 2.1), yield 35.6%. ^{1}H NMR (600 MHz, DMSO-*d$_6$*) δ 9.81 (s, 1H), 9.08 (s, 1H), 8.91(brs, 1H), 8.89 (s, 1H), 8.29 (s, 1H), 8.06 (d, *J* = 5.9 Hz, 1H), 7.90 (d, *J* = 8.3 Hz, 1H), 7.68 (d, *J* = 8.2 Hz, 3H), 7.58 (d, *J* = 8.2 Hz, 2H), 7.53-7.50 (m, 3H), 7.46-7.45 (m, 1H), 6.84 (d, *J* = 8.7 Hz, 2H), 6.29 (d, *J* = 5.5 Hz, 1H), 4.74 (t, *J* = 5.5 Hz, 2H), 4.48 (t, *J* = 5.3 Hz, 2H) ppm. ^{13}C NMR (150 MHz, DMSO-*d$_6$*) δ 160.37, 159.85, 158.15, 156.46, 155.85, 154.90, 153.13, 145.02, 143.21, 142.80, 134.00, 133.33, 131.63, 129.99 (3C), 129.07 (2C), 128.46, 128.12, 127.21, 126.82, 126.66, 121.87, 121.07, 114.89, 114.62 (2C), 98.71, 97.38, 65.95, 46.07 ppm. LC-MS (ESI) *m/z* 601.00 [M + H]$^+$. HRMS (FAB) calculated for $C_{32}H_{25}ClN_{10}O$ [M + H]$^+$: 601.1980, found: 601.1974.

2.2. General synthetic procedure for 4-5 using the copper(I)-catalyzed alkyne-azide cycloaddition (CuAAC) reaction

To a solution of **2a** (1.0 equiv.) and **6a** (1.0 equiv.) in a mixture of DMF, *t*BuOH and water (2:2:1, v/v/v) were added sodium ascorbate (0.2 equvi.) and copper(II) sulfate pentahydrate (0.1 equiv.). The mixture was stirred at 70 °C for 3 h. The resulting mixture was concentrated *in vacuo* and then extracted with DCM (3 X 100 mL). The organic phase was dried with anhydrous sodium sulfate, the solvent evaporated, and the crude product was purified using silica gel chromatography with dichloromethane /methanol gradient

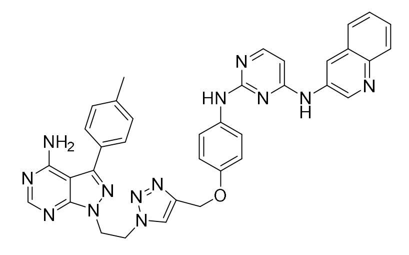

N^2-(4-((1-(2-(4-amino-3-(p-tolyl)-1H-pyrazolo[3,4-d]pyrimidin-1-yl)ethyl)-1H-1,2,3-triazol-4-yl)methoxy)phenyl)-N^4-(quinolin-3-yl)pyrimidine-2,4-diamine (4a) This compound was synthesized using the general procedure depicted above (section 2.2), yield 54.9%. ^{1}H NMR (400 MHz, DMSO-*d*$_6$) δ 9.82 (s, 1H), 9.10 (s, 1H), 8.93 (brs, 2H), 8.19 (s, 1H), 8.12 (s, 1H), 8.08 (d, J = 5.7 Hz, 1H), 7.92 (d, J = 7.6 Hz, 1H), 7.76 (d, J = 6.3 Hz, 1H), 7.60–7.54 (m, 4H), 7.50 (d, J = 8.0 Hz, 2H), 7.32 (d, J = 7.9 Hz, 2H), 6.94 (d, J = 9.0 Hz, 2H), 6.30 (d, J = 5.8 Hz, 1H), 5.05 (s, 2H), 4.91 (t, J = 5.5 Hz, 2H), 4.82 (t, J = 5.5 Hz, 2H), 2.35 (s, 3H) ppm. ^{13}C NMR (100 MHz, DMSO-*d*$_6$) δ. 160.41, 159.86, 158.06, 156.45, 155.75, 154.59, 153.17, 145.07, 144.18, 143.25, 142.83, 138.08, 134.04, 133.99, 129.85, 129.64 (3C), 128.50, 128.17, 128.10 (2C), 127.27, 126.91, 126.79, 124.74, 121.71, 121.06, 114.61 (2C), 98.75, 97.31, 61.32, 48.55, 46.26, 20.86 ppm. HRMS (FAB) calculated for $C_{36}H_{31}N_{13}O$ [M + H]$^+$: 662.2853, found: 662.2849.

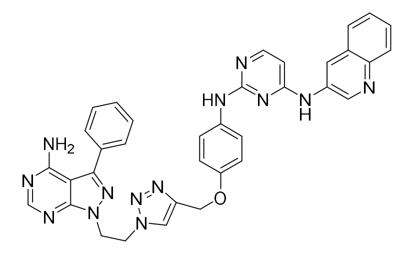

N^2-(4-((1-(2-(4-amino-3-phenyl-1H-pyrazolo[3,4-d]pyramidin-1-yl)ethyl)-1H-1,2,3-triazol-4-yl)methoxy)phenyl)-N^4-(quinolin-3-yl)pyrimidine-2,4-diamine (4b) This compound was synthesized using the general procedure depicted above (section 2.2), yield 66.6%. ^{1}H NMR (600 MHz, DMSO-d_6) δ 9.82 (s, 1H), 9.11 (s, 1H), 8.92 (brs, 2H), 8.21 (s, 1H), 8.14 (s, 1H), 8.08 (d, J = 5.9 Hz, 1H), 7.92 (d, J = 8.3 Hz, 1H), 7.76 (brs, 1H), 7.62 (d, J = 7.3 Hz, 2H), 7.59-7.56 (m, 3H), 7.54-7.51 (m, 3H), 7.46 (t, J = 7.4 Hz, 1H), 6.95 (d, J = 8.7 Hz, 2H), 6.30 (d, J = 5.9 Hz, 1H), 5.05 (s, 2H), 4.92 (t, J = 5.7 Hz, 2H), 4.84 (t, J = 5.7 Hz, 2H) ppm. ^{13}C NMR (150 MHz, DMSO-d_6) δ 160.40, 159.86, 158.05, 156.45, 155.77, 154.64, 153.17, 145.06, 144.15, 143.25, 142.82, 134.02, 134.00, 132.68, 129.05 (3C), 128.65, 128.49, 128.20 (2C), 128.16, 127.26, 126.91, 126.78, 124.74, 121.72, 121.09, 114.60 (2C), 98.72, 97.31, 61.32, 48.54, 46.32 ppm. HRMS (FAB) calculated for $C_{35}H_{29}N_{13}O$ [M + H]$^+$: 648.2691, found: 648.2701.

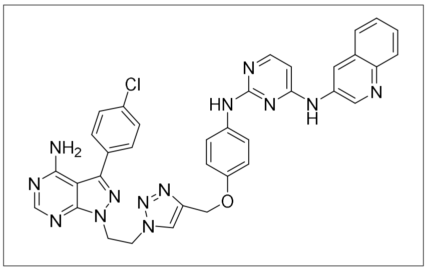

N^2-(4-((1-(2-(4-amino-3-(4-chlorophenyl)-1H-pyrazolo[3,4-d]pyrimidin-1-yl)ethyl)-1H-1,2,3-triazol-4yl)methoxy)phenyl)-N^4-(quinolin-3-yl)pyrimidine-2,4-diamine (4c) This compound was synthesized using the general procedure depicted above (section 2.2), yield 49.4%. ^{1}H NMR (500 MHz, DMSO-d_6) δ 9.83 (s, 1H), 9.12 (s, 1H), 8.94-8.92 (m, 2H), 8.20 (s, 1H), 8.13 (s, 1H), 8.08 (d, J = 5.2 Hz, 1H), 7.92 (d, J = 7.9 Hz, 1H), 7.76 (brs, 1H), 7.61-7.54 (m, 8H), 6.94 (d, J = 8.6 Hz, 2H), 6.30 (d, J = 5.6 Hz, 1H), 5.04 (s, 2H), 4.91-4.90 (m, 2H), 4.84-4.83 (m, 2H) ppm. ^{13}C NMR (125 MHz, DMSO-d_6) δ 162.90, 160.43, 159.85, 158.09, 156.40, 155.84, 154.75, 153.19, 145.08, 143.27, 143.09, 142.85, 134.04, 133.98, 133.40, 131.48, 130.00 (2C), 129.06 (2C), 128.51, 128.19, 127.30, 126.95, 126.82, 124.79, 121.74, 121.13, 114.64 (2C), 98.77, 97.30, 61.31, 48.56, 46.39 ppm. HRMS (FAB) calculated for $C_{35}H_{28}ClN_{13}O$ [M + H]$^+$: 682.2301, found: 682.2305.

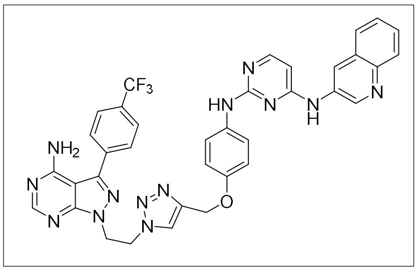

N*[2]*-(4-((1-(2-(4-amino-3-(4-(trifluoromethyl)phenyl)-1H-pyrazolo[3,4-d]pyrimidin-1-yl)ethyl)-1H-1,2,3-triazol-4-yl)methoxy)phenyl)-N*[4]*-(quinolin-3-yl)pyrimidine-2,4-diamine (4d) This compound was synthesized using the general procedure depicted above (section 2.2), yield 73.3%. ^{1}H NMR (600 MHz, DMSO-*d*$_6$) δ 9.82 (s, 1H), 9.11 (s, 1H), 8.94-8.92 (m, 2H), 8.22 (s, 1H), 8.15 (s, 1H), 8.08 (d, J = 5.5 Hz, 1H), 7.92 (d, J = 7.8 Hz, 1H), 7.85 (d, J = 8.7 Hz, 2H), 7.81 (d, J = 8.3 Hz, 2H), 7.76 (brs, 1H), 7.60-7.53 (m, 4H), 6.94 (d, J = 8.7 Hz, 2H), 6.30 (d, J = 6 Hz, 1H), 5.05 (s, 2H), 4.93 (t, J = 5.7 Hz, 2H), 4.86 (t, J = 5.7 Hz, 2H) ppm. ^{13}C NMR (150 MHz, DMSO-*d*$_6$) δ 160.42, 159.87, 158.09, 156.46, 155.89, 154.88, 153.17, 145.07, 143.26, 142.86, 142.84, 136.58, 134.05, 134.01, 128.96 (3C), 128.68 (d, J_{C-F} = 31.6 Hz), 128.50, 128.18, 127.28, 126.91, 126.80, 125.88 (d, J_{C-F} = 3.6 Hz, 2C), 124.80, 124.26 (d, J_{C-F} = 270.0 Hz), 121.71, 121.09, 114.61 (2C), 98.76, 97.38, 61.31, 48.56, 46.48 ppm. HRMS (FAB) calculated for $C_{36}H_{28}F_3N_{13}O$ [M + H]$^+$: 716.2570, found: 716.2572.

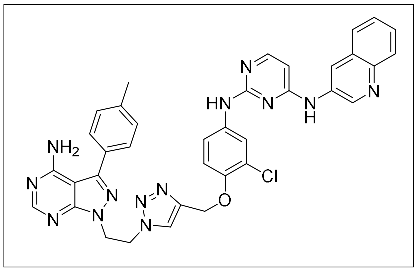

N*[2]*-(4-((1-(2-(4-amino-3-(p-tolyl)-1H-pyrazolo[3,4-d]pyrimidin-1-yl)ethyl)-1H-1,2,3-triazol-4-yl) methoxy)-3-chlorophenyl)-N*[4]*-(quinolin-3-yl)pyrimidine-2,4-diamine (4e) This compound was synthesized using the general procedure depicted above (section 2.2), yield 49.5%. ^{1}H NMR (400 MHz, DMSO-*d*$_6$) δ 9.90 (s, 1H), 9.29 (s, 1H), 8.95 (brs, 2H), 8.18 (s, 1H), 8.15 (s, 1H), 8.12 (d, J = 5.6 Hz, 1H), 7.93 (d, J = 7.8 Hz, 1H), 7.90 (brs, 1H), 7.80 (m, 1H), 7.60-7.53 (m, 3H), 7.49 (d, J = 8.0 Hz, 2H), 7.31 (d, J = 7.9 Hz, 2H), 7.21 (d, J = 9 Hz, 1H), 6.35 (d, J = 5.8 Hz, 1H), 5.12 (s, 2H), 4.93-4.91 (m, 2H), 4.83-4.82 (m, 2H), 2.35 (s, 3H) ppm. ^{13}C NMR (100 MHz, DMSO-*d*$_6$) δ 160.46, 159.38, 158.09, 156.38, 155.79, 154.63, 148.05, 145.20, 144.23, 143.39, 142.48, 138.12, 135.07, 133.98, 129.88, 129.69 (2C), 128.53, 128.18, 128.14 (2C), 127.48, 127.10, 126.87, 124.94, 121.31, 121.24, 120.91, 119.11, 114.78, 99.45, 97.30, 62.43, 48.61, 46.32, 20.92 ppm. HRMS (FAB) calculated for $C_{36}H_{30}ClN_{13}O$ [M + H]$^+$: 696.2463, found: 696.2468.

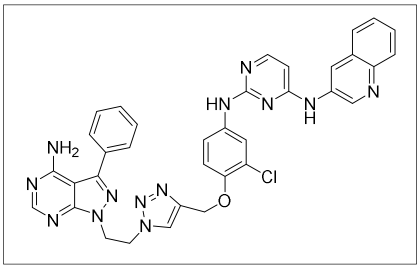

N^2-(4-((1-(2-(4-amino-3-phenyl-1H-pyrazolo[3,4-d]pyrimidin-1-yl)ethyl)-1H-1,2,3-triazol-4-yl) methoxy)-3-chlorophenyl)-N^4-(quinolin-3-yl)pyrimidine-2,4-diamine (4f) This compound was synthesized using the general procedure depicted above (section 2.2), yield 71.5%. ^{1}H NMR (600 MHz, DMSO-d_6) δ 9.86 (s, 1H), 9.27 (s, 1H), 8.96 (s, 1H), 8.92 (brs, 1H), 8.19 (s, 1H), 8.15 (s, 1H), 8.12 (d, J = 5.9 Hz, 1H), 7.93 (d, J = 8.3 Hz, 1H), 7.89 (brs, 1H), 7.82 (d, J = 6.9 Hz, 1H), 7.62 (d, J = 7.6 Hz, 2H), 7.60-7.56 (m, 3H), 7.54-7.51 (m, 2H), 7.47 (t, J = 7.3 Hz, 1H), 7.21 (d, J = 8.7 Hz, 1H), 6.35 (d, J = 5.5 Hz, 1H), 5.12 (s, 2H), 4.94 (t, J = 5.7 Hz, 2H), 4.84 (t, J = 5.7 Hz, 2H) ppm. ^{13}C NMR (150 MHz, DMSO-d_6) δ 160.43, 159.36, 158.04, 156.34, 155.77, 154.66, 148.05, 145.19, 144.17, 143.38, 142.45, 135.06, 133.92, 132.68, 129.04 (2C), 128.66, 128.48, 128.19 (2C), 128.13, 127.42, 127.03, 126.79, 124.86, 121.34, 121.27, 120.92, 119.11, 114.84, 99.36, 97.31, 62.48, 48.54, 46.31 ppm. HRMS (FAB) calculated for $C_{35}H_{28}ClN_{13}O$ [M + H]$^+$: 682.2307, found: 682.2310.

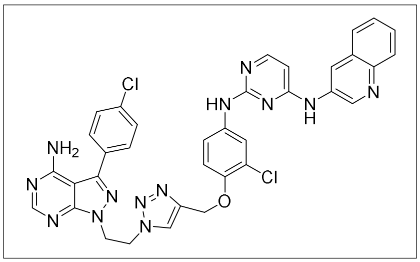

N^2-(4-((1-(2-(4-amino-3-(4-chlorophenyl)-1H-pyrazolo[3,4-d]pyrimidin-1-yl)ethyl)-1H-1,2,3-triazol-4-yl)methoxy)-3-chlorophenyl)-N^4-(quinolin-3-yl)pyrimidine-2,4-diamine (4g) This compound was synthesized using the general procedure depicted above (section 2.2), yield =39.2%. ^{1}H NMR (800 MHz, DMSO-d_6) δ 9.87 (s, 1H), 9.28 (s, 1H), 8.96 (s, 1H), 8.92 (brs, 1H), 8.19 (s, 1H), 8.15 (s, 1H), 8.12 (d, J = 5.6 Hz, 1H), 7.93 (d, J = 8.2 Hz, 1H), 7.90 (brs, 1H), 7.81 (brs, 1H), 7.60 (d, J = 8.4 Hz, 2H), 7.58-7.54 (m, 5H), 7.22 (d, J = 9.0 Hz, 1H), 6.35 (d, J = 5.7 Hz, 1H), 5.13 (s, 2H), 4.93 (t, J = 5.7 Hz, 2H), 4.83 (t, J = 5.7 Hz, 2H) ppm. ^{13}C NMR (200 MHz, DMSO-d_6) δ 160.45, 159.37, 158.08, 156.34, 155.83, 154.75, 148.04, 145.20, 143.39, 143.08, 142.47, 135.08, 133.94, 133.38, 131.47, 129.97 (2C), 129.04 (2C), 128.50, 128.15, 127.44, 127.05, 126.81, 124.92, 121.33, 121.26, 120.90, 119.10, 114.82, 99.39, 97.28, 62.45, 48.55, 46.37 ppm. HRMS (FAB) calculated for $C_{35}H_{27}Cl_2N_{13}O$ [M + H]$^+$: 716.1917, found: 716.1920.

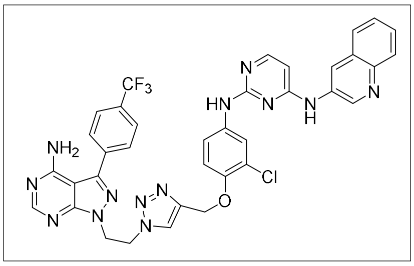

N^2-(4-((1-(2-(4-amino-3-(4-(trifluoromethyl)phenyl)-1H-pyrazolo[3,4-d]pyrimidin-1-yl)ethyl)-1H-1,2,3-triazol-4-yl)methoxy)-3-chlorophenyl)-N^4-(quinolin-3-yl)pyrimidine-2,4-diamine (4h)

This compound was synthesized using the general procedure depicted above (section 2.2), yield 68.1%. ^{1}H NMR (600 MHz, DMSO-d_6) δ 9.87 (s, 1H), 9.27 (s, 1H), 8.96 (s, 1H), 8.92 (brs, 1H), 8.21 (s, 1H), 8.17 (s, 1H), 8.12 (d, J = 5.5 Hz, 1H), 7.93 (d, J = 8.3 Hz, 1H), 7.89 (brs, 1H), 7.84 (d, J = 8.7 Hz, 2H), 7.81 (d, J = 8.2 Hz, 3H), 7.59-7.54 (m, 3H), 7.22 (d, J = 9.2 Hz, 1H), 6.35 (d, J = 5.5 Hz, 1H), 5.13 (s, 2H), 4.94 (t, J = 5.7 Hz, 2H), 4.86 (t, J = 5.7 Hz, 2H) ppm. ^{13}C NMR (150 MHz, DMSO-d_6) δ 160.44, 159.36, 158.08, 156.32, 155.87, 154.87, 148.05, 145.19, 143.38, 142.82, 142.48, 136.57, 135.08, 133.93, 128.93 (2C), 128.66 (d, J_{C-F} = 31.6 Hz), 128.48, 128.14, 127.42, 127.03, 126.79, 125.86 (d, J_{C-F} = 3.6 Hz, 2C), 124.92, 124.26 (d, J_{C-F} = 270.7 Hz), 121.31, 121.25, 120.91, 119.10, 114.83, 99.38, 97.36, 62.46, 48.55, 46.46 ppm. HRMS (FAB) calculated for $C_{36}H_{27}ClF_3N_{13}O$ [M + H]$^+$: 750.2180, found: 750.2174.

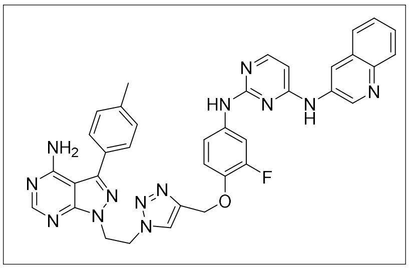

N^2-(4-((1-(2-(4-amino-3-(p-tolyl)-1H-pyrazolo[3,4-d]pyrimidin-1-yl)ethyl)-1H-1,2,3-triazol-4-yl) methoxy)-3-fluorophenyl)-N^4-(quinolin-3-yl)pyrimidine-2,4-dia mine (4i) This compound was synthesized using the general procedure depicted above (section 2.2), yield 72.2%. ^{1}H NMR (400 MHz, DMSO-d_6) δ 9.88 (s, 1H), 9.32 (s, 1H), 8.95 (s, 1H), 8.93 (brs, 1H), 8.18 (s, 1H), 8.17 (s, 1H), 8.12 (d, J = 5.7 Hz, 1H), 7.94 (d, J = 8.0 Hz, 1H), 7.81 (t, J = 6.5 Hz, 1H), 7.76 (s, 1H), 7.61-7.54 (m, 2H), 7.49 (d, J = 7.9 Hz, 2H), 7.35-7.30 (m, 3H), 7.18 (t, J = 9.3 Hz, 1H), 6.35 (d, J = 5.7 Hz, 1H), 5.11 (s, 2H), 4.92 (t, J = 5.8 Hz, 2H), 4.82 (t, J = 5.6 Hz, 2H), 2.35 (s, 3H) ppm. ^{13}C NMR (100 MHz, DMSO-d_6) δ 160.42, 159.35, 158.07, 156.38, 155.75, 154.59, 151.46 (d, J_{C-F} = 239.9 Hz), 145.20, 144.18, 143.38, 142.45, 140.04, 138.07, 135.01 (d, J_{C-F} = 9.7 Hz), 133.90, 129.85, 129.64 (3C), 128.51, 128.13, 128.10 (3C), 127.33, 127.07, 126.86, 124.98, 121.33, 116.04,

115.09, 97.29, 62.53, 48.58, 46.29, 20.87 ppm. HRMS (FAB) calculated for $C_{36}H_{30}FN_{13}O$ [M + H]$^+$: 680.2759, found: 680.2755.

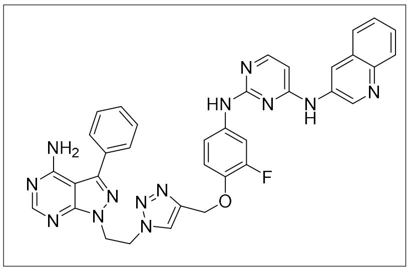

N^2-(4-((1-(2-(4-amino-3-phenyl-1H-pyrazolo[3,4-d]pyrimidin-1-yl)ethyl)-1H-1,2,3-triazol-4-yl)methoxy)-3-fluorophenyl)-N^4-(quinolin-3-yl)pyrimidine-2,4-diamine (4j) This compound was synthesized using the general procedure depicted above (section 2.2), yield 74.5%. ^{1}H NMR (400 MHz, DMSO-*d₆*) δ 9.88 (s, 1H), 9.31 (s, 1H), 8.96 (d, *J* = 2.3 Hz, 1H), 8.92 (brs, 1H), 8.20 (s, 1H), 8.18 (s, 1H), 8.12 (d, *J* = 5.7 Hz, 1H), 7.94 (d, *J* = 7.8 Hz, 1H), 7.81 (t, *J* = 7.6 Hz, 1H), 7.76 (s, 1H), 7.63-7.59 (m, 3H), 7.57 (d, *J* = 2 Hz, 1H), 7.56-7.44 (m, 4H), 7.35 (d, *J* = 8.6 Hz, 1H), 7.19 (t, *J* = 9.3 Hz, 1H), 6.35 (d, *J* = 5.8 Hz, 1H), 5.11 (s, 2H), 4.92 (t, *J* = 5.5 Hz, 2H), 4.83 (t, *J* = 5.5 Hz, 2H) ppm. ^{13}C NMR (100 MHz, DMSO-d_6) δ 160.42, 159.35, 158.06, 156.37, 155.77, 154.64, 151.46 (d, $J_{C\text{-}F}$ = 239.7 Hz), 145.21, 144.16, 143.39, 142.44, 140.16, 134.97, 133.89, 132.68, 129.05 (3C), 128.67, 128.51, 128.20 (2C), 128.13, 127.32, 127.07, 126.86, 124.99, 121.35, 116.08 (d, $J_{C\text{-}F}$ = 2.3 Hz), 115.09, 99.33, 97.31, 62.56, 48.57, 46.33 ppm. HRMS (FAB) calculated for $C_{35}H_{28}FN_{13}O$ [M + H]$^+$: 666.2602, found: 666.2603.

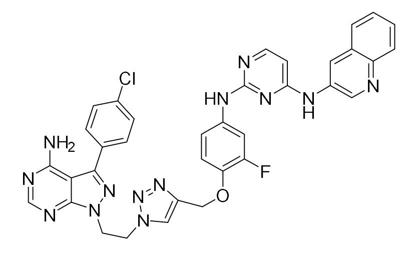

N^2-(4-((1-(2-(4-amino-3-(4-chlorophenyl)-1H-pyrazolo[3,4-d]pyrimidin-1-yl)ethyl)-1H-1,2,3-triazol-4-yl)methoxy)-3-fluorophenyl)-N^4-(quinolin-3-yl)pyrimidine-2,4-diamine (4k) This compound was synthesized using the general procedure depicted above (section 2.2), yield 78.7%. ^{1}H NMR (400 MHz, DMSO-d_6) δ 9.89 (s, 1H), 9.32 (s, 1H), 8.95 (s, 1H), 8.93 (brs, 1H), 8.19 (s, 1H), 8.17 (s, 1H), 8.12 (d, *J* = 5.6 Hz, 1H), 7.93 (d, *J* = 7.9 Hz, 1H), 7.83-7.76 (m, 2H), 7.61-7.53 (m, 6H), 7.35 (d, *J* = 8.7 Hz, 1H), 7.19 (t, *J* = 9.3 Hz, 1H), 6.36 (d, *J* = 5.7 Hz, 1H), 5.11 (s, 2H), 4.92 (t, *J* = 5.3 Hz, 2H), 4.82 (t, *J* = 5.2 Hz, 2H) ppm. ^{13}C NMR (100 MHz, DMSO-d_6) δ 162.90, 160.44, 159.36, 158.09, 156.38, 155.84, 154.74, 151.46 (d, $J_{C\text{-}F}$ = 239.8 Hz), 145.22,

143.39, 143.08, 142.46, 140.10 (d, $J_{C\text{-}F}$ = 10.9 Hz), 135.03 (d, $J_{C\text{-}F}$ = 9.7 Hz), 133.92, 133.39, 131.47, 129.99 (2C), 129.04 (2C), 128.52, 128.15, 127.22 (d, $J_{C\text{-}F}$ = 26.2 Hz), 126.87, 125.03, 121.35, 116.06 (d, $J_{C\text{-}F}$ = 2.2 Hz), 115.09, 107.83 (d, $J_{C\text{-}F}$ = 22.1 Hz), 99.38, 97.29, 62.53, 48.58, 46.40 ppm. HRMS (FAB) calculated for $C_{35}H_{27}ClFN_{13}O$ $[M + H]^+$: 700.2212, found: 700.2217.

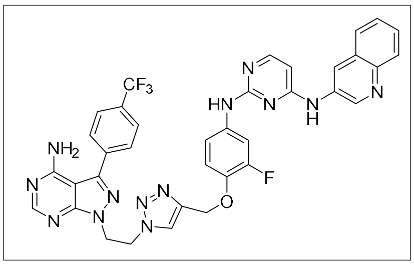

N^2-(4-((1-(2-(4-amino-3-(4-(trifluoromethyl)phenyl)-1H-pyrazolo[3,4-d]pyrimidin-1-yl)ethyl)-1H-1,2,3-triazol-4-yl)methoxy)-3-fluorophenyl)-N^4-(quinolin-3-yl)pyrimidine-2,4-diamine (4l) This compound was synthesized using the general procedure depicted above (section 2.2), yield = 76.0%. ^{1}H NMR (600 MHz, DMSO-d_6) δ 9.86 (s, 1H), 9.29 (s, 1H), 8.96 (d, J = 2.3 Hz, 1H), 8.91 (brs, 1H), 8.21 (s, 1H), 8.18 (s, 1H), 8.12 (d, J = 5.5 Hz, 1H), 7.93 (d, J = 8.3 Hz, 1H), 7.84 (d, J = 8.3 Hz, 2H), 7.80 (d, J = 8.2 Hz, 2H), 7.77 (d, $J_{C\text{-}F}$ = 13.7 Hz, 1H), 7.60-7.54 (m, 2H), 7.35 (d, J = 8.7 Hz, 1H), 7.19 (t, J = 9.4 Hz, 1H), 6.35 (d, J = 5.5 Hz, 1H), 5.11 (s, 2H), 4.93 (t, J = 5.5 Hz, 2H), 4.85 (t, J = 5.7 Hz, 2H) ppm. ^{13}C NMR (150 MHz, DMSO-d_6) δ 160.40, 159.33, 158.04, 156.31, 155.82, 154.83, 151.47 (d, $J_{C\text{-}F}$ = 240.6 Hz), 145.20, 143.38, 142.78, 142.46, 140.08 (d, $J_{C\text{-}F}$ = 11.5 Hz), 136.55, 135.03 (d, $J_{C\text{-}F}$ = 9.3 Hz), 133.85, 128.89 (2C), 128.66 (d, $J_{C\text{-}F}$ = 31.6 Hz), 128.46, 128.09, 127.26, 127.00, 126.77, 125.79 (d, $J_{C\text{-}F}$ = 3.6 Hz, 2C), 124.93, 124.21 (d, $J_{C\text{-}F}$ = 270.7 Hz), 121.36, 116.12, 115.08, 107.81 (d, $J_{C\text{-}F}$ = 22.3 Hz), 99.29, 97.36, 62.60, 48.50, 46.43 ppm. HRMS (FAB) calculated for $C_{36}H_{27}F_4N_{13}O$ $[M + H]^+$: 734.2476, found: 734.2463.

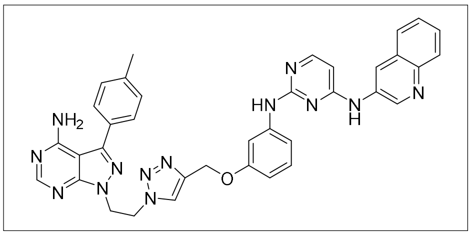

N^2-(3-((1-(2-(4-amino-3-(p-tolyl)-1H-pyrazolo[3,4-d]pyrimidin-1-yl)ethyl)-1H-1,2,3-triazol-4-yl)methoxy)phenyl)-N^4-(quinolin-3-yl)pyrimidine-2,4-diamine (5a) This compound was synthesized using the general procedure depicted above (section 2.2), yield 48.1%. ^{1}H NMR (800 MHz, DMSO-d_6) δ 9.87 (s, 1H), 9.30 (s, 1H), 9.00 (brs, 1H), 8.96 (d, J = 2.2 Hz, 1H), 8.19 (s, 1H), 8.12 (d, J = 5.6 Hz, 1H), 8.02 (brs, 1H), 7.92 (d, J = 8.2 Hz, 1H),

7.84 (d, J = 7.2 Hz, 1H), 7.58 (t, J = 7.5 Hz, 1H), 7.54 (t, J = 7.2 Hz, 1H), 7.49 (d, J = 7.9 Hz, 2H), 7.47 (s, 1H), 7.34 (d, J = 8.1 Hz, 1H), 7.32 (d, J = 7.8 Hz, 2H), 7.16 (t, J = 8.1 Hz, 1H), 6.64 (dd, J = 8.0 Hz, 2.12 Hz, 1H), 6.36 (d, J = 5.7 Hz, 1H), 5.01 (s, 2H), 4.89 (t, J = 5.8 Hz, 2H), 4.80 (t, J = 5.8 Hz, 2H), 2.34 (s, 3H) ppm. ^{13}C NMR (200 MHz, DMSO-d_6) δ 160.40, 159.43, 158.34, 158.06, 156.32, 155.74, 154.56, 145.11, 144.16, 143.31, 142.66, 141.85, 138.06, 133.95, 129.84, 129.64 (2C), 129.14, 128.47, 128.17, 128.08 (2C), 127.36, 126.97, 126.82, 124.68, 121.16, 112.15, 107.03, 106.09, 99.49, 97.31, 60.86, 48.52, 46.26, 20.84 ppm. HRMS (FAB) calculated for $C_{36}H_{31}N_{13}O$ [M + H]$^+$: 662.2847, found: 662.2855.

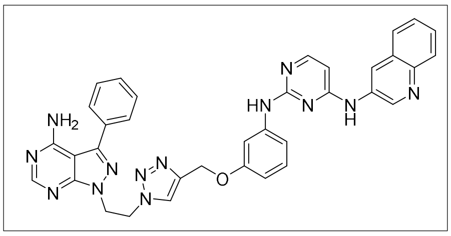

N^2-(3-((1-(2-(4-amino-3-phenyl-1H-pyrazolo[3,4-d]pyrimidin-1-yl)ethyl)-1H-1,2,3-triazol-4-yl)methoxy)phenyl)-N^4-(quinolin-3-yl)pyrimidine-2,4-diamine (5b) This compound was synthesized using the general procedure depicted above (section 2.2), yield 61.7%. ^{1}H NMR (800 MHz, DMSO-d_6) δ 9.88 (s, 1H), 9.31 (s, 1H), 9.00 (s, 1H), 8.96 (d, J = 2.1 Hz, 1H), 8.20 (s, 1H), 8.13 (d, J = 5.6 Hz, 1H), 8.04 (s, 1H), 7.93 (d, J = 8.2 Hz, 1H), 7.84 (d, J = 7.2 Hz, 1H), 7.61 (d, J = 7.0 Hz, 2H), 7.58 (td, J = 7.5 Hz, 1.36 Hz, 1H), 7.55-7.50 (m, 4H), 7.46 (t, J = 7.4 Hz, 1H), 7.35 (dd, J = 8.1 Hz, 1.3 Hz, 1H), 7.17 (t, J = 8.1 Hz, 1H), 6.64 (dd, J = 8.1 Hz, 2.1 Hz, 1H), 6.37 (d, J = 5.7 Hz, 1H), 5.01 (s, 2H) 4.90 (t, J = 5.8 Hz, 2H), 4.82 (t, J = 5.8 Hz, 2H) ppm. ^{13}C NMR (200 MHz, DMSO-d_6) δ 160.42, 159.42, 158.36, 158.07, 156.30, 155.79, 154.64, 145.13, 144.16, 143.33, 142.68, 141.86, 133.96, 132.69, 129.17, 129.07 (2C), 128.66, 128.49, 128.21 (2C), 128.19, 127.38, 126.99, 126.84, 124.71, 121.19, 112.18, 107.11, 106.07, 99.51, 97.33, 60.88, 48.54, 46.32 ppm. HRMS (FAB) calculated for $C_{35}H_{29}N_{13}O$ [M + H]$^+$: 648.2696, found: 648.2689.

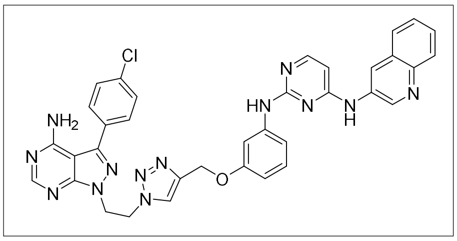

N^2-(3-((1-(2-(4-amino-3-(4-chlorophenyl)-1H-pyrazolo[3,4 d]pyrimidin-1-yl)ethyl)-1H-1,2,3-triazol-4-yl)methoxy)phenyl)-N^4-(quinolin-3-yl)pyrimidine-2,4-diamine (5c) This compound was synthesized using the general procedure depicted above (section 2.2), yield 54.8%. ^{1}H NMR (400 MHz, DMSO-d_6) δ 9.88 (s, 1H), 9.31 (s, 1H), 9.00 (s, 1H), 8.96 (d, J = 2.2 Hz, 1H), 8.20 (s, 1H), 8.13 (d, J = 5.6 Hz, 1H), 8.03 (s, 1H), 7.92 (d, J = 7.9 Hz, 1H), 7.84 (d, J = 7.4 Hz, 1H), 7.61-7.51 (m, 7H), 7.35 (d, J = 8.0 Hz, 1H), 7.17 (t, J = 8.1 Hz, 1H), 6.63 (dd, J = 8.1 Hz, 1.8 Hz, 1H), 6.37 (d, J = 5.7 Hz, 1H), 5.01 (s, 2H), 4.89 (t, J = 5.2 Hz, 2H), 4.81 (t, J = 5.6 Hz, 2H) ppm. ^{13}C NMR (100 MHz, DMSO-d_6) δ 160.42, 159.43, 158.36, 158.10, 156.33, 155.84, 154.72, 145.13, 143.32, 143.07, 142.69, 141.89, 133.98, 133.39, 131.46, 129.98 (2C), 129.17, 129.06 (2C), 128.49, 128.20, 127.39, 126.99, 126.84, 124.74, 121.18, 112.15, 107.03, 106.06, 99.52, 97.30, 60.87, 48.53, 46.38 ppm. HRMS (FAB) calculated for $C_{35}H_{28}ClN_{13}O$ [M + H]$^+$: 682.2301, found: 682.2306.

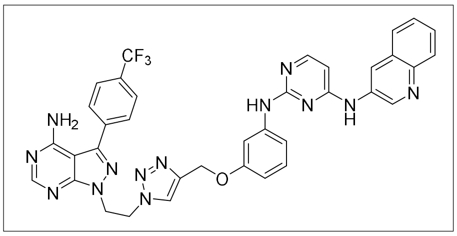

N^2-(3-((1-(2-(4-amino-3-(4-(trifluoromethyl)phenyl)-1H-pyrazolo[3,4-d]pyrimidin-1-yl)ethyl)-1H-1,2,3-triazol-4-yl)methoxy)phenyl)-N^4-(quinolin-3-yl)pyrimidine-2,4-diamine (5d) This compound was synthesized using the general procedure depicted above (section 2.2), yield = 57.8%. ^{1}H NMR (600 MHz, DMSO-d_6) δ 9.86 (s, 1H), 9.29 (s, 1H), 8.99 (brs, 1H), 8.96 (d, J = 2.3 Hz, 1H), 8.22 (s, 1H), 8.12 (d, J = 5.5 Hz, 1H), 8.04 (s, 1H), 7.92 (d, J = 8.3 Hz, 1H), 7.85 (d, J = 8.2 Hz, 3H), 7.80 (d, J = 8.3 Hz, 2H), 7.58 (t, J = 7.2 Hz, 1H), 7.54 (t, J = 6.9 Hz, 1H), 7.51 (brs, 1H), 7.34 (d, J = 7.8 Hz, 1H), 7.16 (t, J = 8.3 Hz, 1H), 6.63 (dd, J = 8.3 Hz, 2.3 Hz, 1H), 6.36 (d, J = 6.0 Hz, 1H), 5.01 (s, 2H), 4.91 (t, J = 5.7 Hz, 2H), 4.84 (t, J = 5.7 Hz, 2H) ppm. ^{13}C NMR (150 MHz, DMSO-d_6) δ 160.40, 159.41, 158.33, 158.07, 156.28, 155.86, 154.84, 145.11, 143.31, 142.80, 142.70, 141.86, 136.55, 133.93, 129.10, 128.91 (2C), 128.66 (d, J_{C-F} = 31.6 Hz), 128.45, 128.16, 127.34, 126.95, 126.78, 125.85 (d, J_{C-F} = 3.6 Hz, 2C), 124.68, 124.23 (d, J_{C-F} = 270.0 Hz),

121.18, 112.13, 107.06, 106.04, 99.46, 97.37, 60.88, 48.49, 46.44 ppm. HRMS (FAB) calculated for $C_{36}H_{28}F_3N_{13}O$ $[M + H]^+$: 716.2565, found: 716.2573.

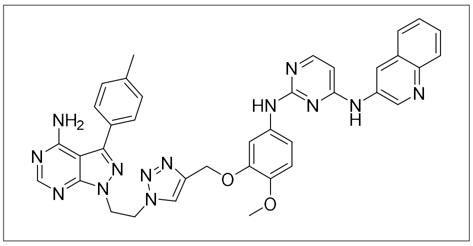

N^2-(3-((1-(2-(4-amino-3-(p-tolyl)-1H-pyrazolo[3,4-d]pyrimidin-1-yl)ethyl)-1H-1,2,3-triazol-4-yl)methoxy)-4-methoxyphenyl)-N^4-(quinolin-3-yl)pyrimidine-2,4-diamine (5e) This compound was synthesized using the general procedure depicted above (section 2.2), yield 79.4%. ^{1}H NMR (400 MHz, DMSO-d_6) δ 9.83 (s, 1H), 9.09 (s, 1H), 8.96 (s, 1H), 8.94 (s, 1H), 8.19 (s, 1H), 8.09 (d, *J* = 5.6 Hz, 1H), 8.03 (brs, 1H), 7.91 (d, *J* = 8.0 Hz, 1H), 7.77 (d, *J* = 6.2 Hz, 1H), 7.59-7.52 (m, 2H), 7.50 (d, *J* = 8.0 Hz, 2H), 7.45 (brs, 1H), 7.33-7.28 (m, 3H), 6.90 (d, *J* = 8.8 Hz, 1H), 6.30 (d, *J* = 5.7 Hz, 1H), 4.96 (s, 2H), 4.89 (t, *J* = 5.5 Hz, 2H), 4.80 (t, *J* = 5.4 Hz, 2H), 3.72 (s, 3H), 2.36 (s, 3H) ppm. ^{13}C NMR (100 MHz, DMSO-d_6) δ 160.38, 159.77, 158.08, 156.44, 155.78, 154.58, 147.26, 145.06, 144.27, 144.19, 143.23, 142.53, 138.08, 134.08, 129.85, 129.65 (3C), 128.50, 128.18, 128.10 (3C), 127.29, 126.91, 126.78, 124.87, 121.02, 112.59, 107.47, 98.87, 97.31, 61.63, 55.87, 48.50, 46.26, 20.88 ppm. HRMS (FAB) calculated for $C_{37}H_{33}N_{13}O_2$ $[M + H]^+$: 692.2958, found: 692.2854.

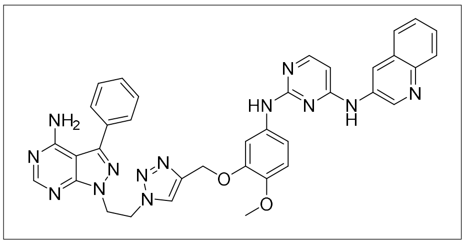

N^2-(3-((1-(2-(4-amino-3-phenyl-1H-pyrazolo[3,4-d]pyrimidin-1-yl)ethyl)-1H-1,2,3-triazol-4-yl)methoxy)-4-methoxyphenyl)-N^4-(quinolin-3-yl)pyrimidine-2,4-diamine (5f) This compound was synthesized using the general procedure depicted above (section 2.2), yield 72.4%. ^{1}H NMR (600 MHz, DMSO-d_6) δ 9.81 (s, 1H), 9.07 (s, 1H), 8.94 (brs, 2H), 8.21 (s, 1H), 8.10 (brs, 1H), 8.04 (s, 1H), 7.91 (d, *J* = 8.3 Hz, 1H), 7.77 (brs, 1H), 7.62 (d, *J* = 8.0 Hz, 2H), 7.57 (t, *J* = 6.9 Hz, 1H), 7.54-7.51 (m, 3H), 7.48-7.44 (m, 2H), 7.29 (d, *J* = 8.8 Hz, 1H), 6.90 (d, *J* = 8.7 Hz, 1H), 6.31 (d, *J* = 5.5 Hz, 1H), 4.96 (s, 2H), 4.90 (t, *J* = 5.7 Hz, 2H), 4.81 (t, *J* = 5.7 Hz, 2H), 3.72 (s, 3H) ppm. ^{13}C NMR (150 MHz, DMSO-d_6) δ 160.37, 159.78, 158.06, 156.40,

155.78, 154.63, 147.28, 145.05, 144.28, 144.16, 143.24, 142.55, 134.08, 132.67, 129.04 (3C), 128.65, 128.49, 128.19 (3C), 127.27, 126.89, 126.76, 124.83, 121.04, 112.91, 112.64, 107.54, 98.87, 97.35, 61.66, 55.89, 48.46, 46.28 ppm. HRMS (FAB) calculated for $C_{36}H_{31}N_{13}O_2$ [M + H]$^+$: 678.2802, found: 678.2792.

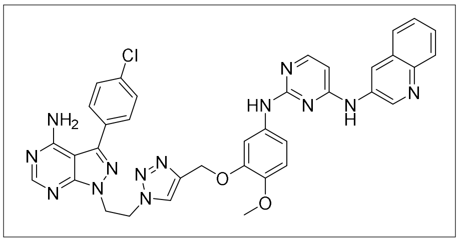

N^2-(3-((1-(2-(4-amino-3-(4-chlorophenyl)-1H-pyrazolo[3,4-d]pyrimidin-1-yl)ethyl)-1H-1,2,3-triazol-4-yl)methoxy)-4-methoxyphenyl)-N^4-(quinolin-3-yl)pyrimidine-2,4-diamine (5g) This compound was synthesized using the general procedure depicted above (section 2.2), yield 46.5%. ^{1}H NMR (400 MHz, DMSO-*d₆*) δ 9.83 (s, 1H), 9.09 (s, 1H), 8.96 (brs, 1H), 8.94 (d, *J* = 2 Hz, 1H), 8.20 (s, 1H), 8.09 (d, *J* = 5.7 Hz, 1H), 8.04 (s, 1H), 7.91 (d, *J* = 8.0 Hz, 1H), 7.77 (d, *J* = 6.2 Hz, 1H), 7.61-7.51 (m, 6H), 7.45 (s, 1H), 7.30 (dd, *J* = 8.6 Hz, 2.0 Hz, 1H), 6.90 (d, *J* = 8.7 Hz, 1H), 6.31 (d, *J* = 5.7 Hz, 1H), 4.96 (s, 2H), 4.89 (t, *J* = 5.5 Hz, 2H), 4.81 (t, *J* = 5.3 Hz, 2H), 3.72 (s, 3H) ppm. ^{13}C NMR (100 MHz, DMSO-d_6) δ 160.38, 159.77, 158.09, 156.43, 155.85, 154.71, 147.27, 145.07, 144.25, 143.24, 143.06, 142.55, 134.08, 133.37, 131.46, 129.99 (3C), 129.04 (2C), 128.50, 128.19, 127.29, 126.90, 126.78, 124.90, 121.03, 112.89, 112.59, 107.47, 98.88, 97.30, 61.63, 55.86, 48.49, 46.34 ppm. HRMS (FAB) calculated for $C_{36}H_{30}ClN_{13}O_2$ [M + H]$^+$: 712.2412, found: 712.2403.

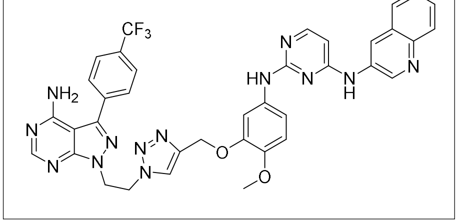

N^2-(3-((1-(2-(4-amino-3-(4-(trifluoromethyl)phenyl)-1H-pyrazolo[3,4-d]pyrimidin-1-yl)ethyl)-1H-1,2,3-triazol-4-yl)methoxy)-4-methoxyphenyl)-N^4-(quinolin-3-yl)pyrimidine-2,4-diamine (5h) This compound was synthesized using the general procedure depicted above (section 2.2), yield 80.0 %. ^{1}H NMR (400 MHz, DMSO-d_6) δ 9.83 (s, 1H), 9.09 (s, 1H), 8.96 (brs, 1H), 8.94 (d, *J* = 2.04 Hz, 1H), 8.22 (s, 1H), 8.09 (d, *J* = 5.8 Hz, 1H), 8.06 (s, 1H), 7.91 (d, *J* = 8.0 Hz, 1H), 7.86-7.76 (m, 4H), 7.59-7.51 (m, 2H), 7.46 (s, 1H), 7.30 (dd, *J* =

8.6 Hz, 2.0 Hz, 1H), 6.90 (d, J = 8.8 Hz, 1H), 6.31 (d, J = 5.6 Hz, 1H), 4.97 (s, 2H), 4.90 (t, J = 5.3 Hz, 2H), 4.83 (t, J = 5.6 Hz, 2H), 3.72 (s, 3H) ppm. ^{13}C NMR (100 MHz, DMSO-d_6) δ 160.38, 159.77, 158.10, 156.41, 155.90, 154.84, 147.27, 145.06, 144.24, 143.24, 142.82, 142.57, 136.57, 134.08, 128.95 (3C), 128.81, 128.50, 128.19, 127.29, 126.90, 126.76, 125.86 (d, J_{C-F} = 3.7 Hz, 2C), 124.91, 124.28 (d, J_{C-F} = 270.4 Hz), 121.02, 112.89, 112.56, 107.46, 98.86, 97.39, 61.64, 55.83, 48.49, 46.45 ppm. HRMS (FAB) calculated for $C_{37}H_{30}F_3N_{13}O_2$ [M + H]$^+$: 746.2676, found: 746.2668.

3. ^{1}H- and ^{13}C-NMR spectra

^{1}H NMR of compound **3a** (600 MHz, DMSO-d_6)

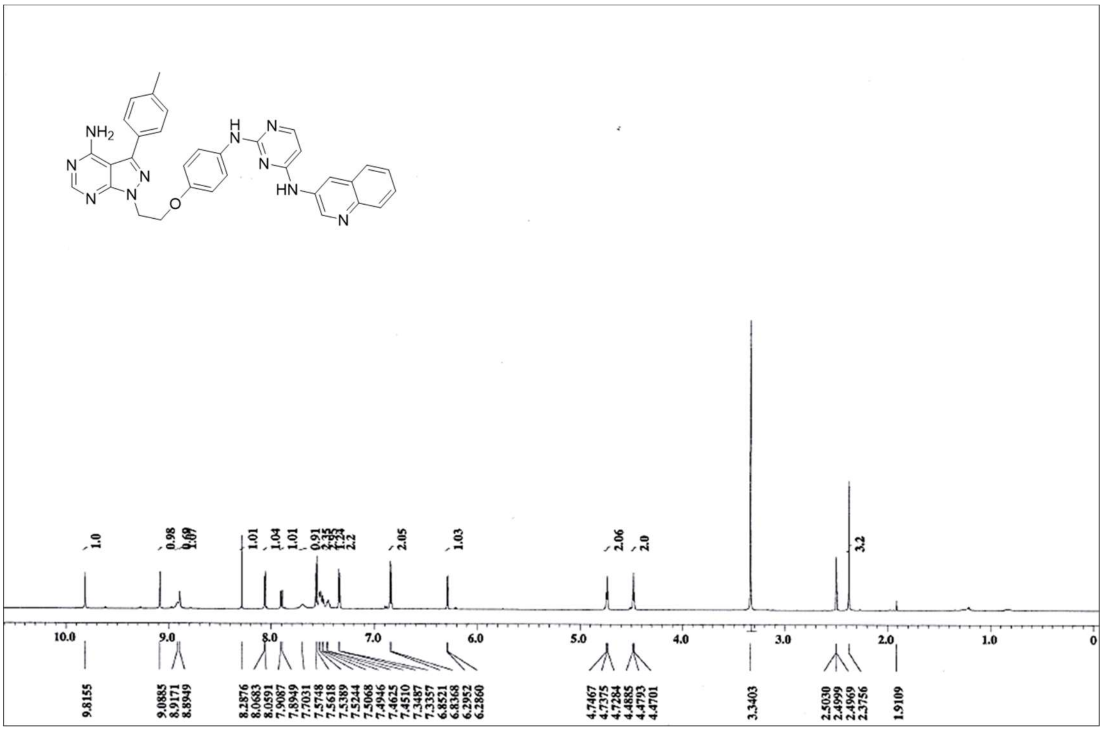

^{13}C NMR of compound **3a** (150 MHz, DMSO-d_6)

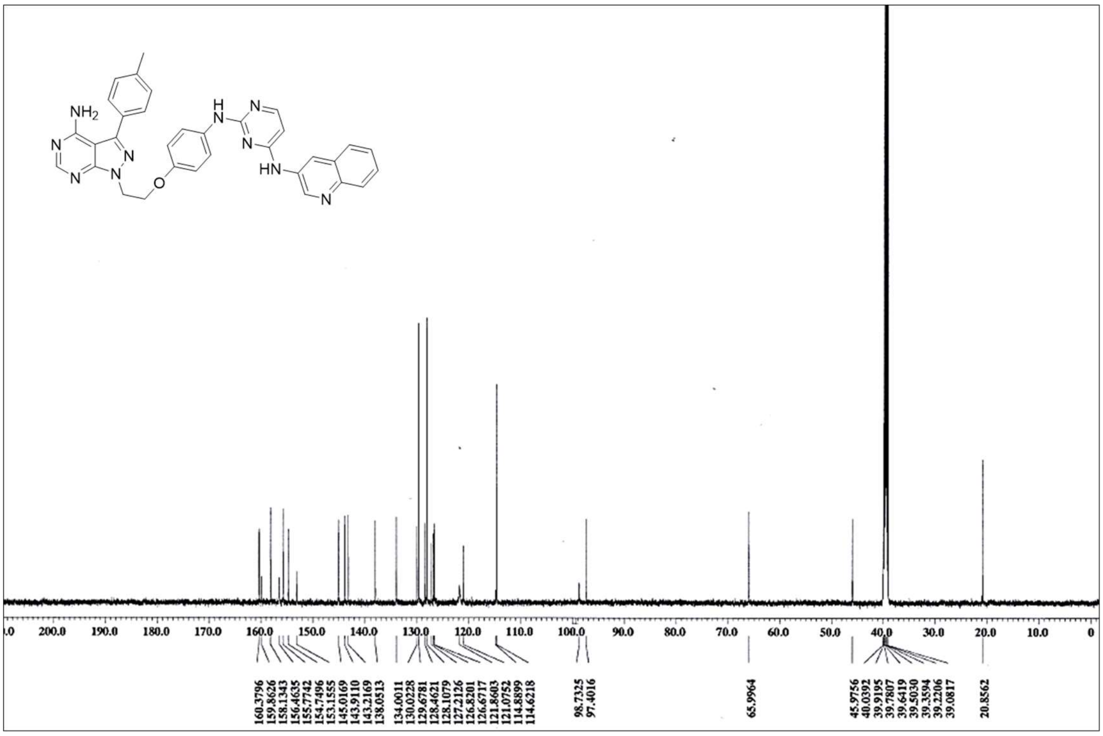

^{1}H NMR of compound **3b** (600 MHz, DMSO-d_6)

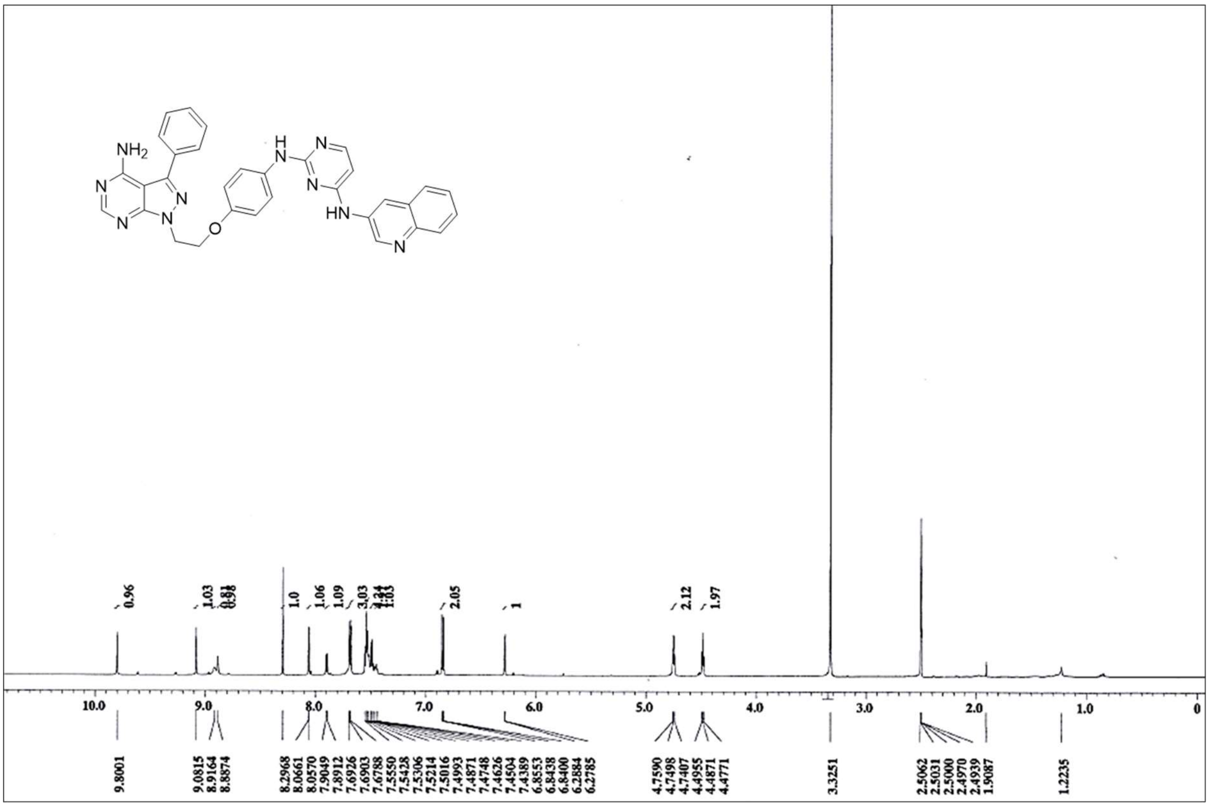

^{13}C NMR of compound **3b** (150 MHz, DMSO-d_6)

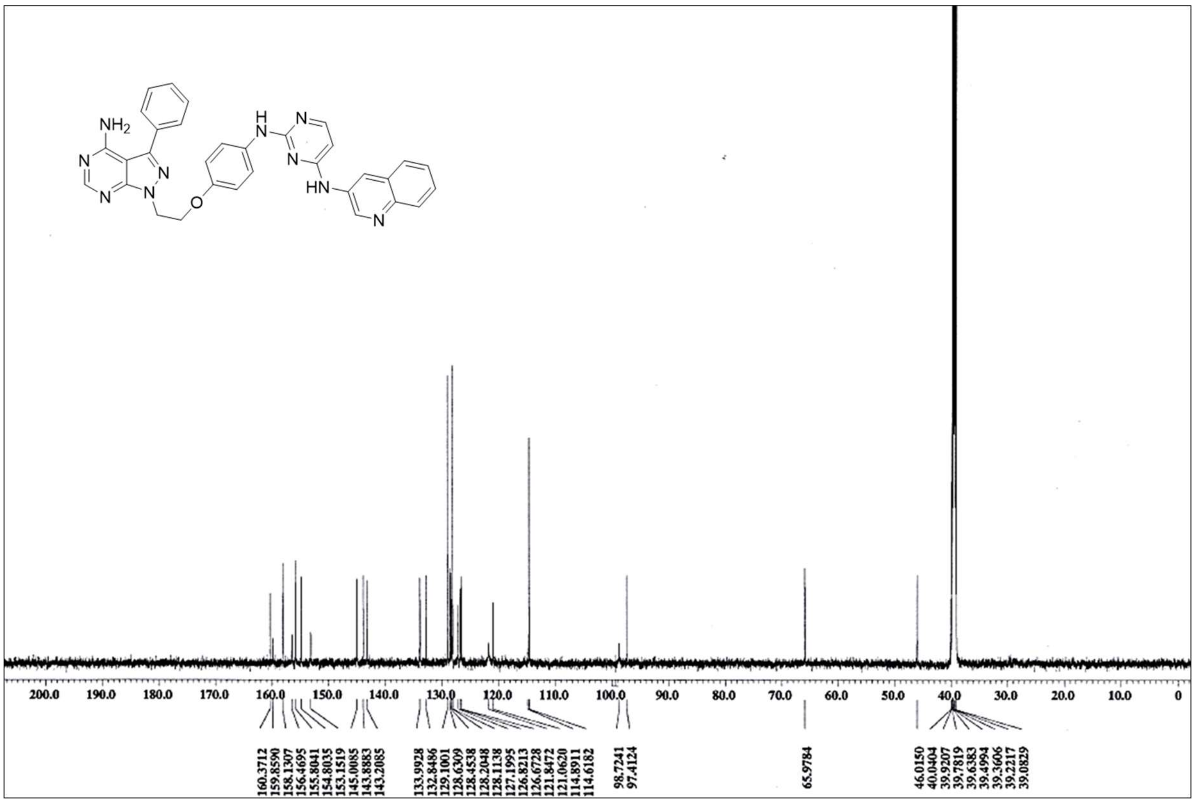

^{1}H NMR of compound **3c** (600 MHz, DMSO-d_6)

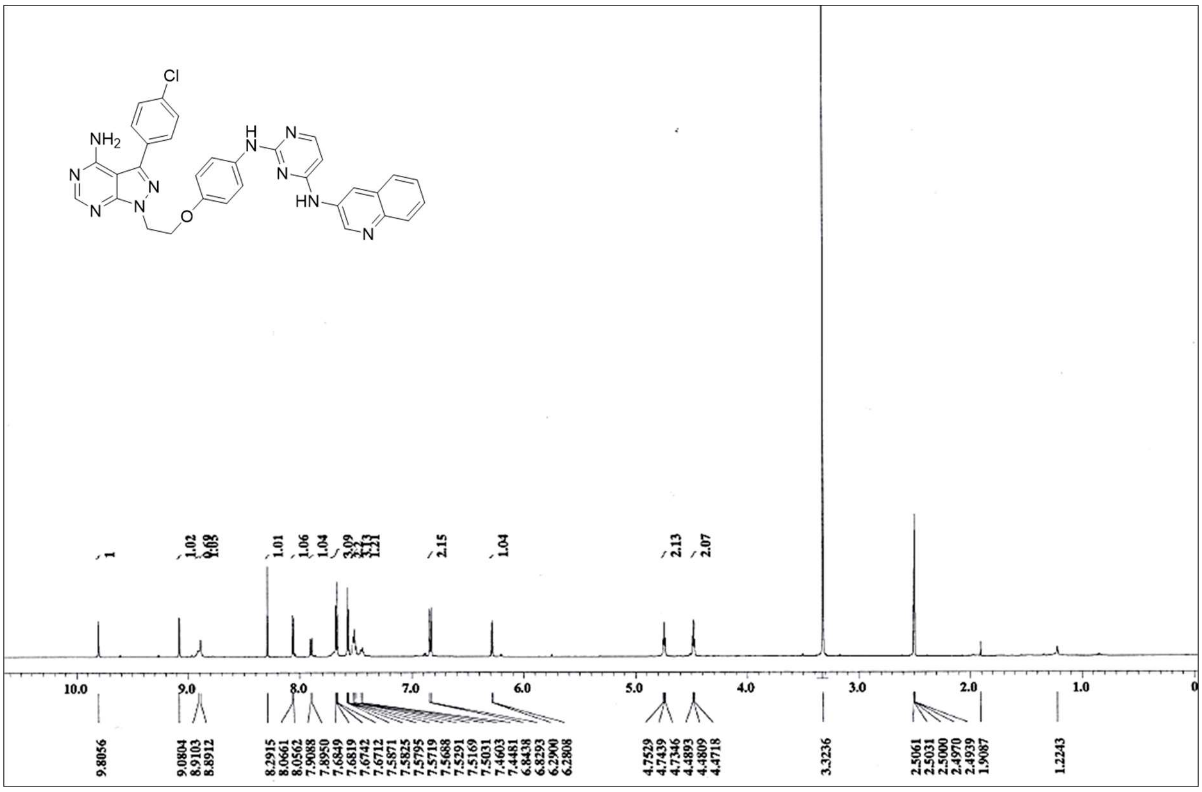

^{13}C NMR of compound **3c** (150 MHz, DMSO-d_6)

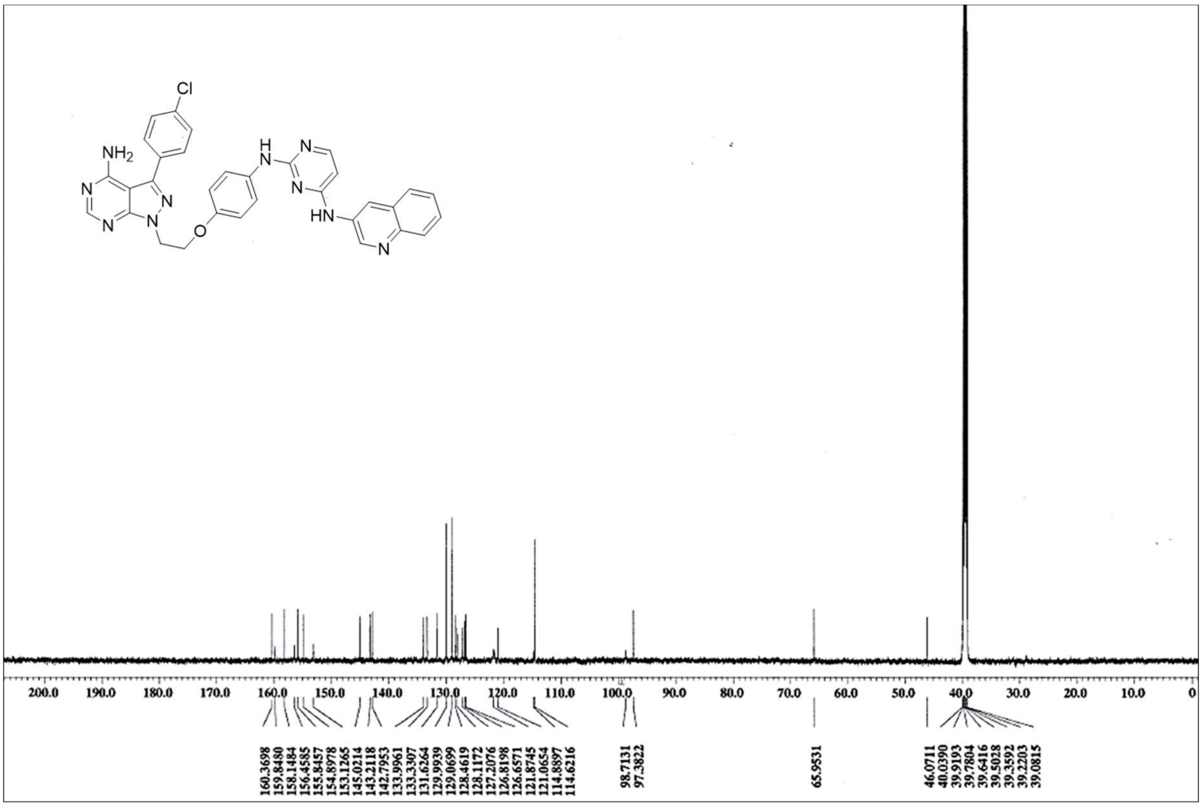

^{1}H NMR of compound **4a** (400 MHz, DMSO-d_6)

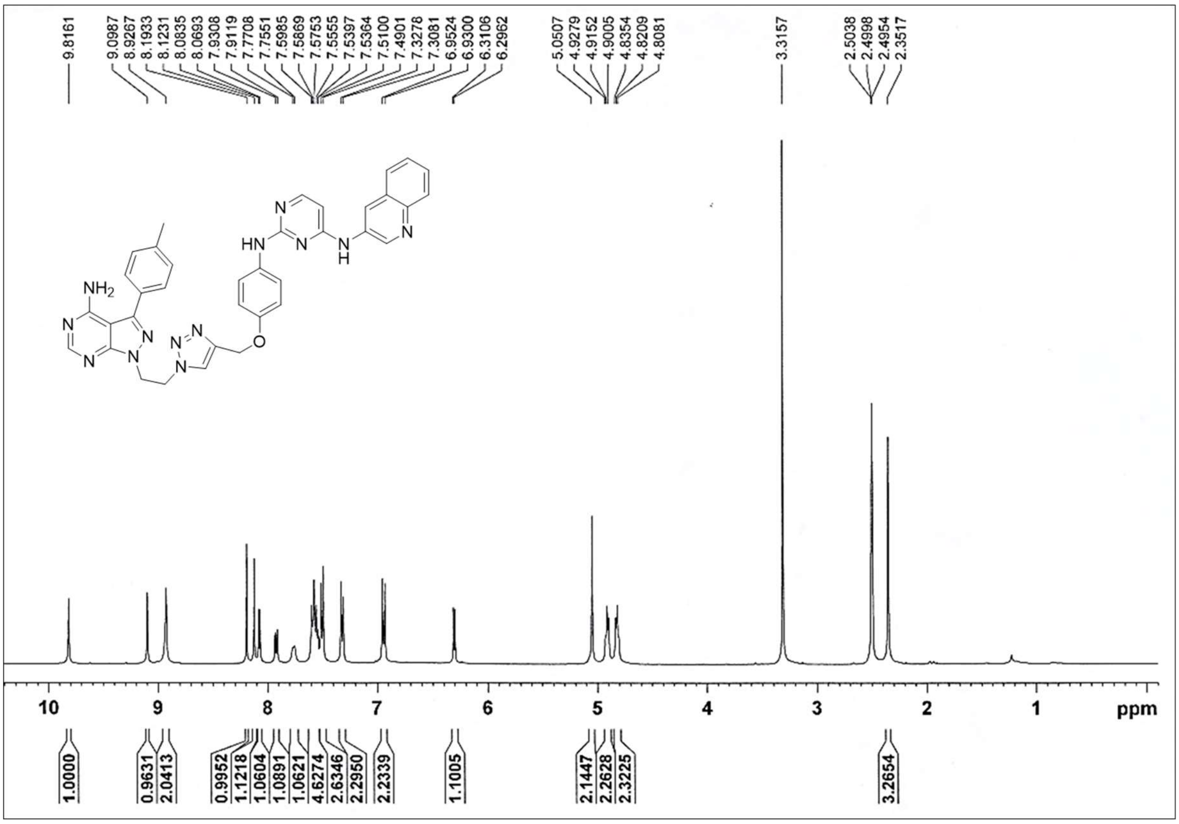

^{13}C NMR of compound **4a** (100 MHz, DMSO-d_6)

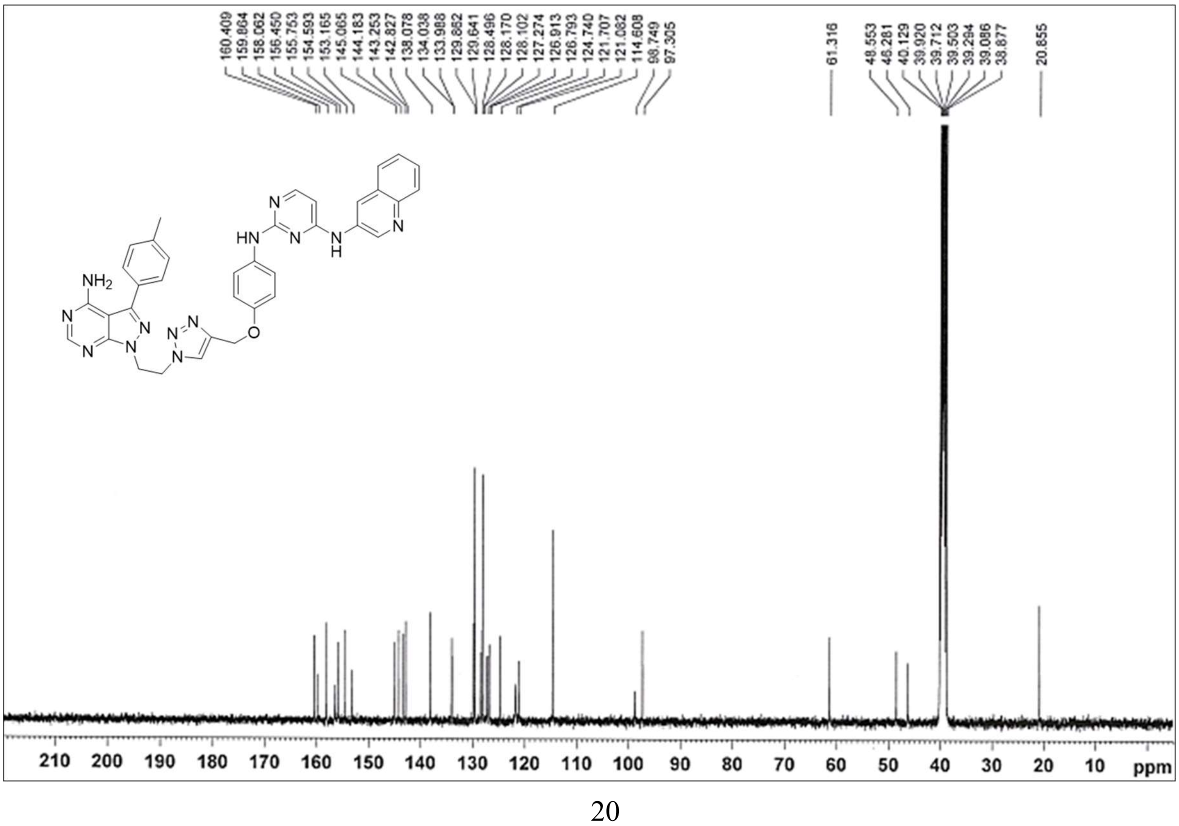

^{1}H NMR of compound **4b** (600 MHz, DMSO-d_6)

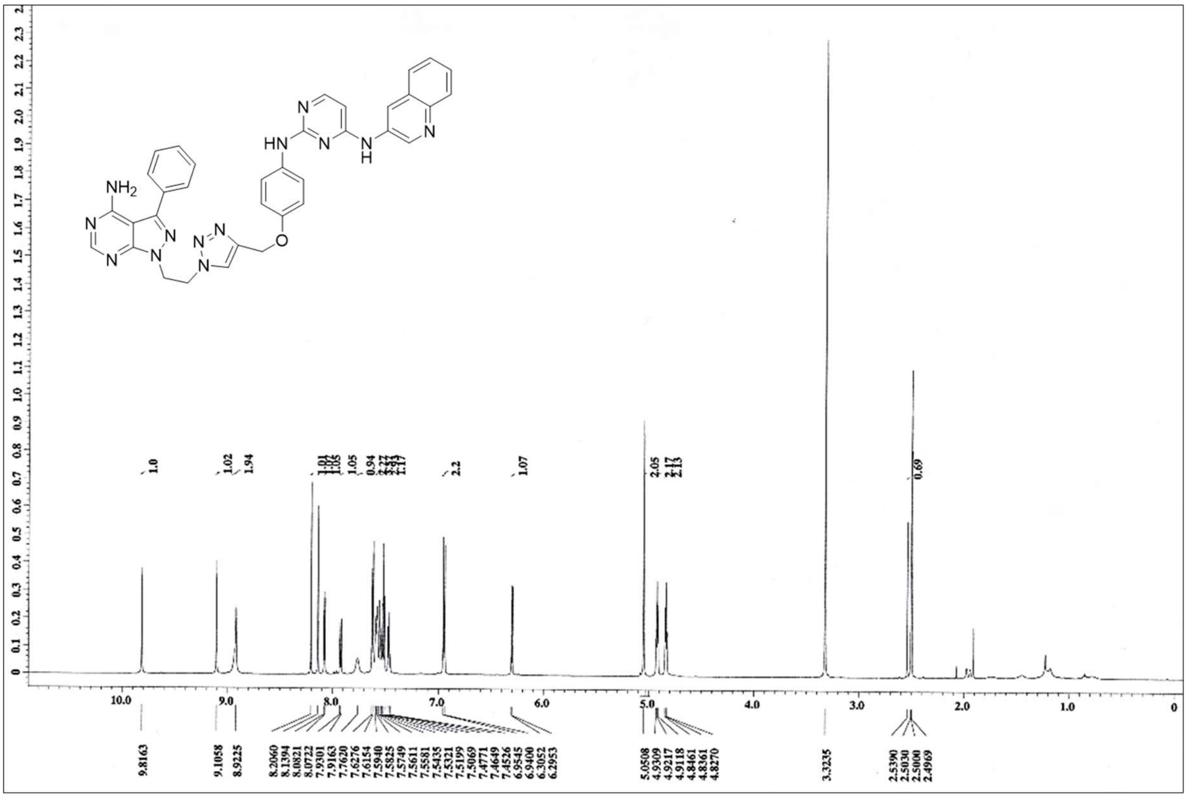

^{13}C NMR of compound **4b** (150 MHz, DMSO-d_6)

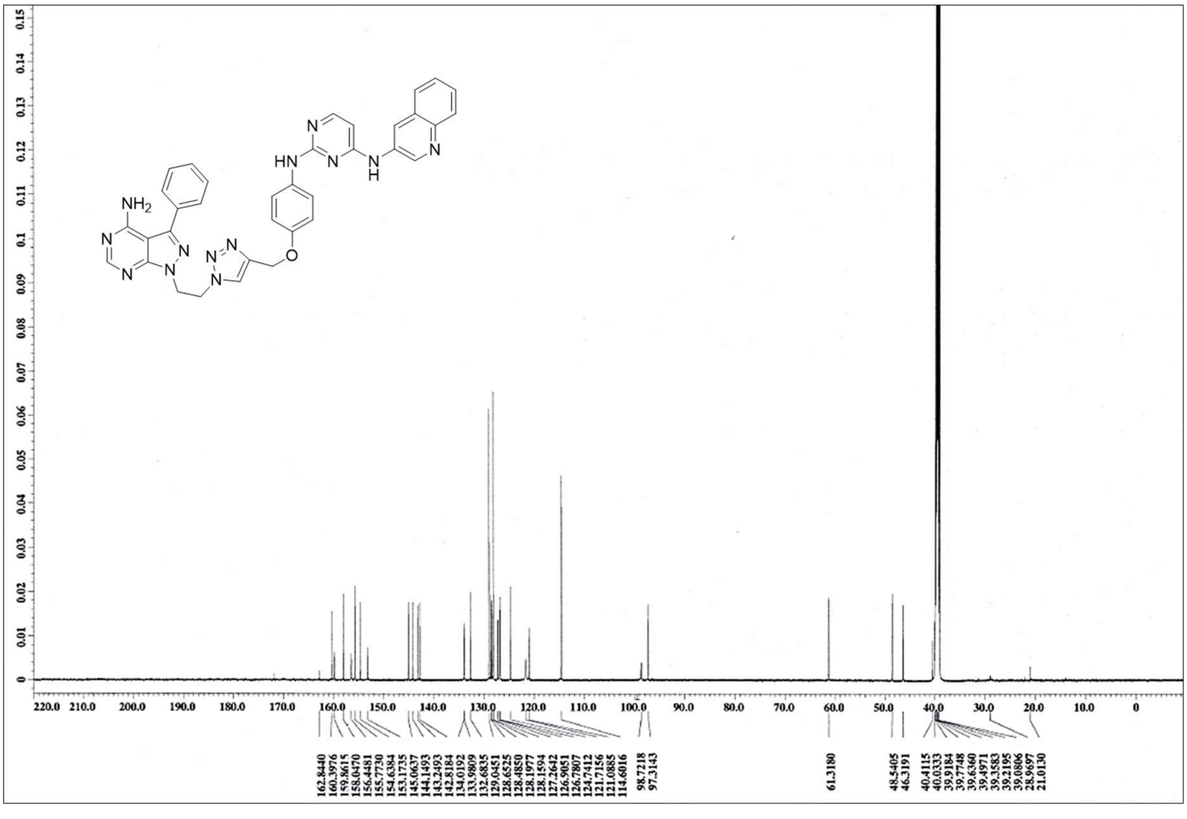

^{1}H NMR of compound **4c** (500 MHz, DMSO-d_6)

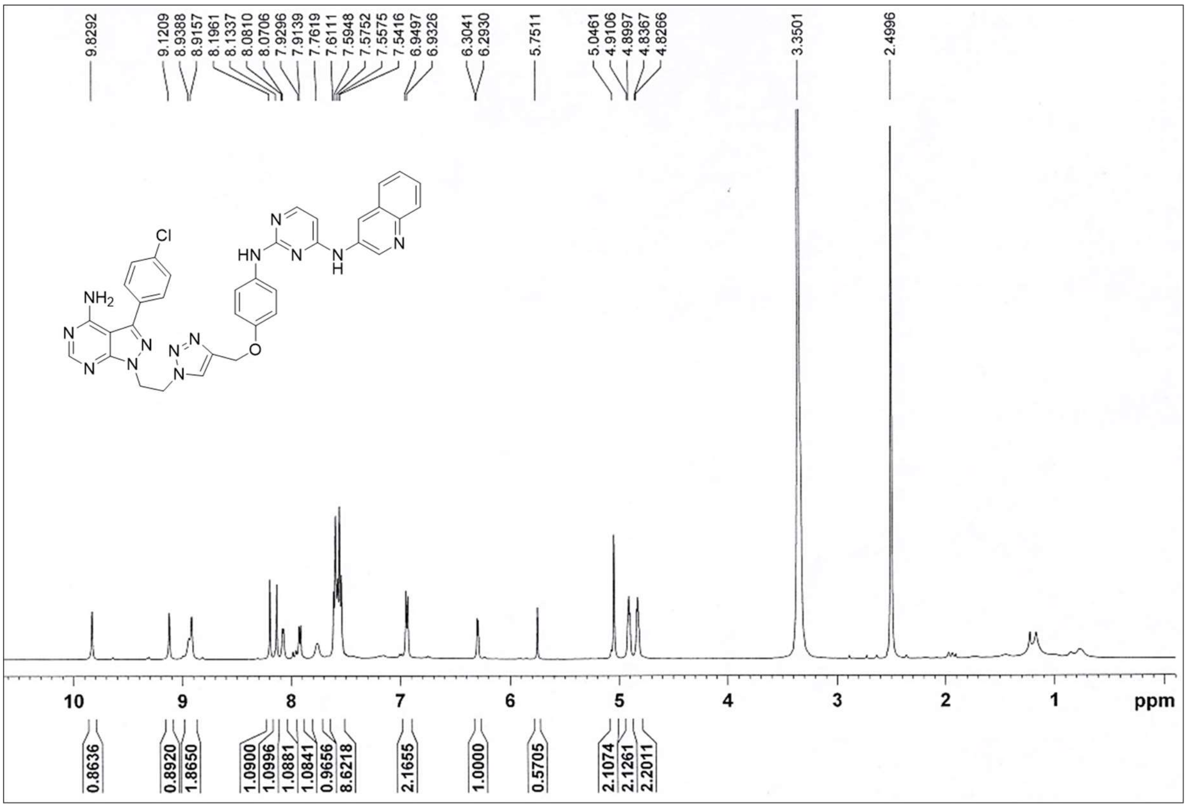

^{13}C NMR of compound **4c** (125 MHz, DMSO-d_6)

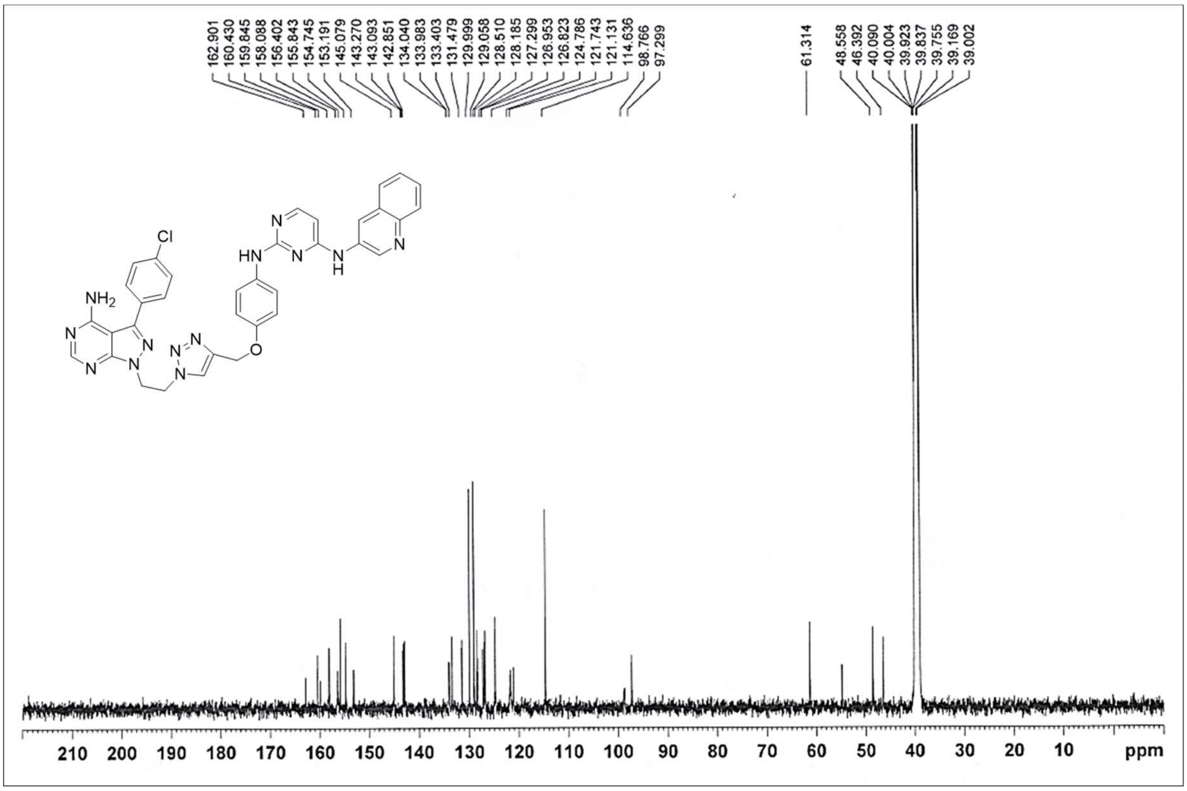

^{1}H NMR of compound **4d** (600 MHz, DMSO-d_6)

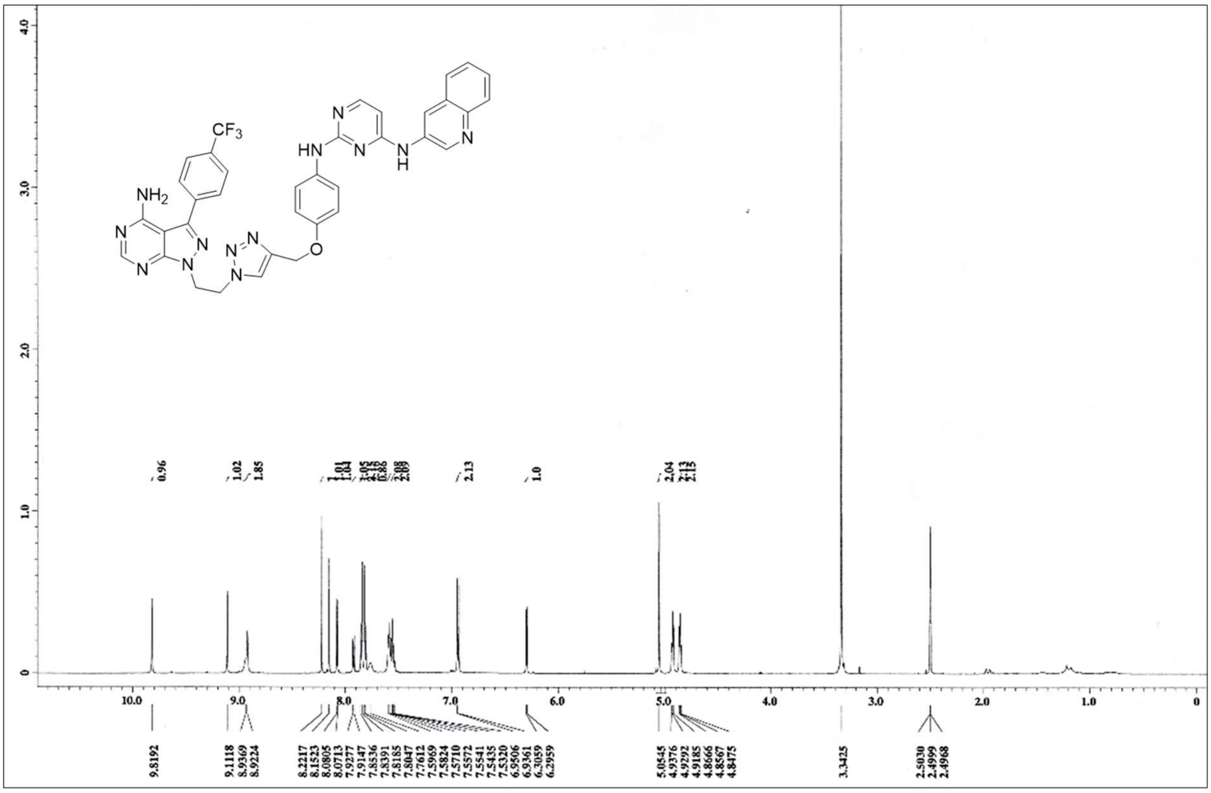

^{13}C NMR of compound **4d** (150 MHz, DMSO-d_6)

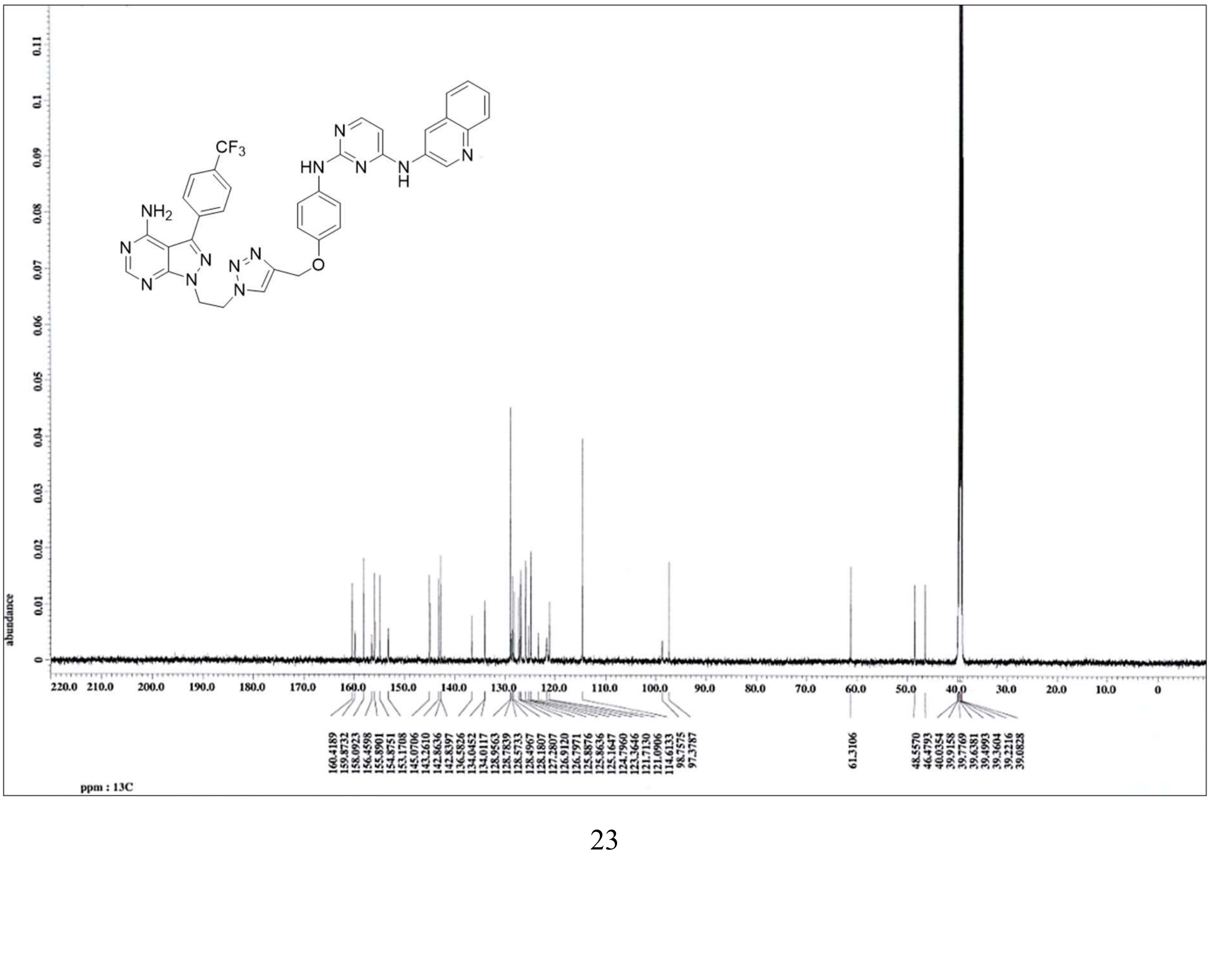

^{1}H NMR of compound **4e** (400 MHz, DMSO-d_6)

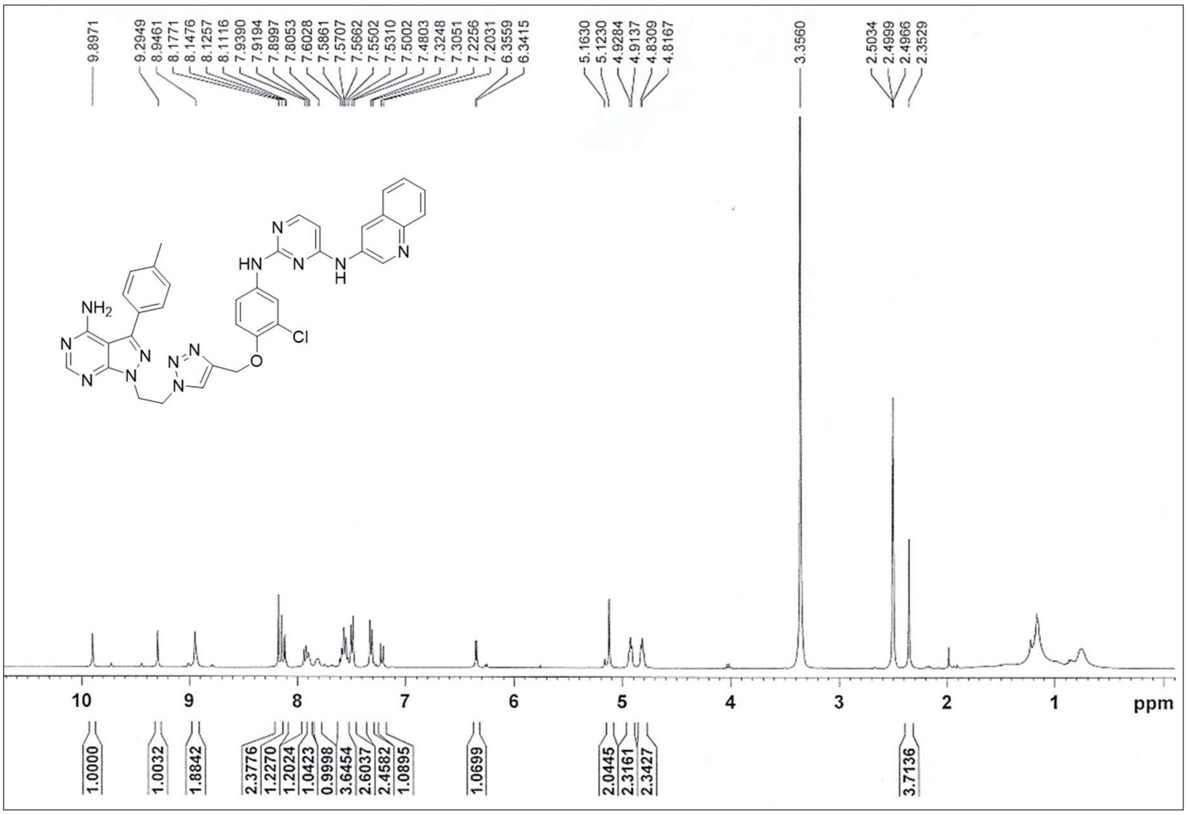

^{13}C NMR of compound **4e** (100 MHz, DMSO-d_6)

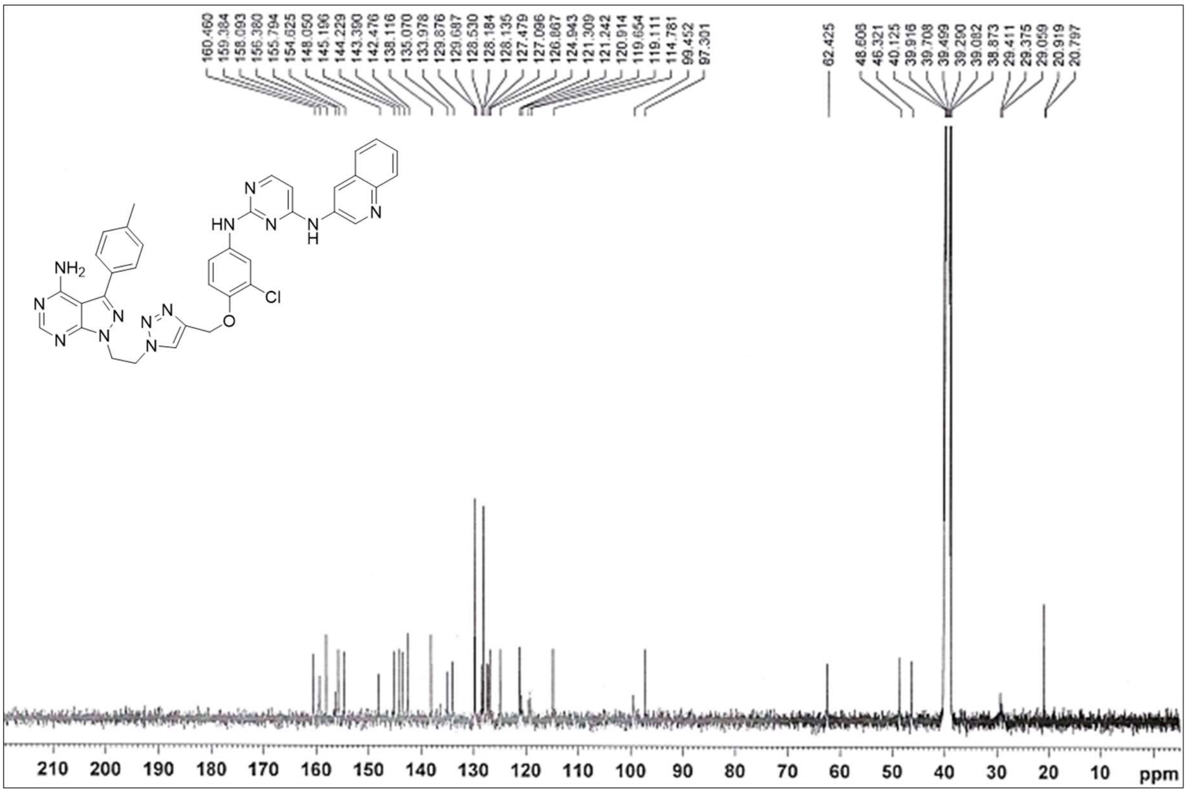

^{1}H NMR of compound **4f** (600 MHz, DMSO-d_6)

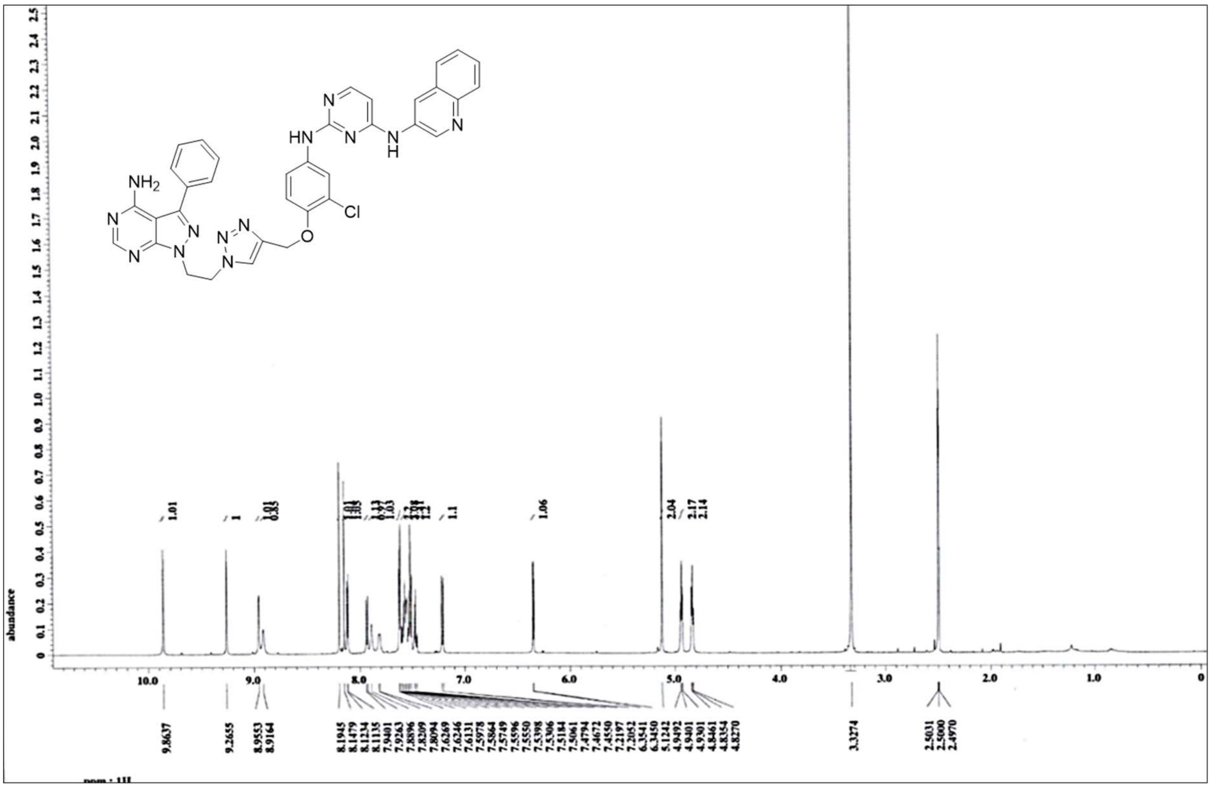

^{13}C NMR of compound **4f** (150 MHz, DMSO-d_6)

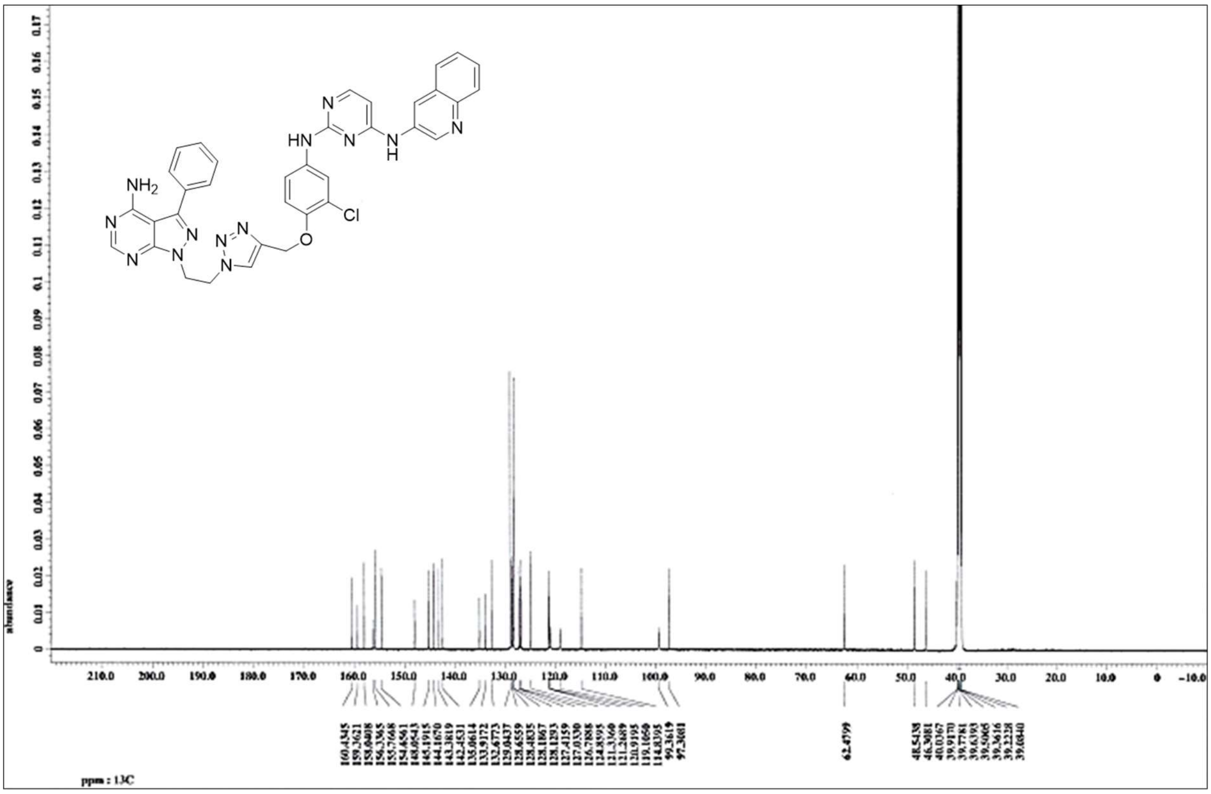

^{1}H NMR of compound **4g** (800 MHz, DMSO-d_6)

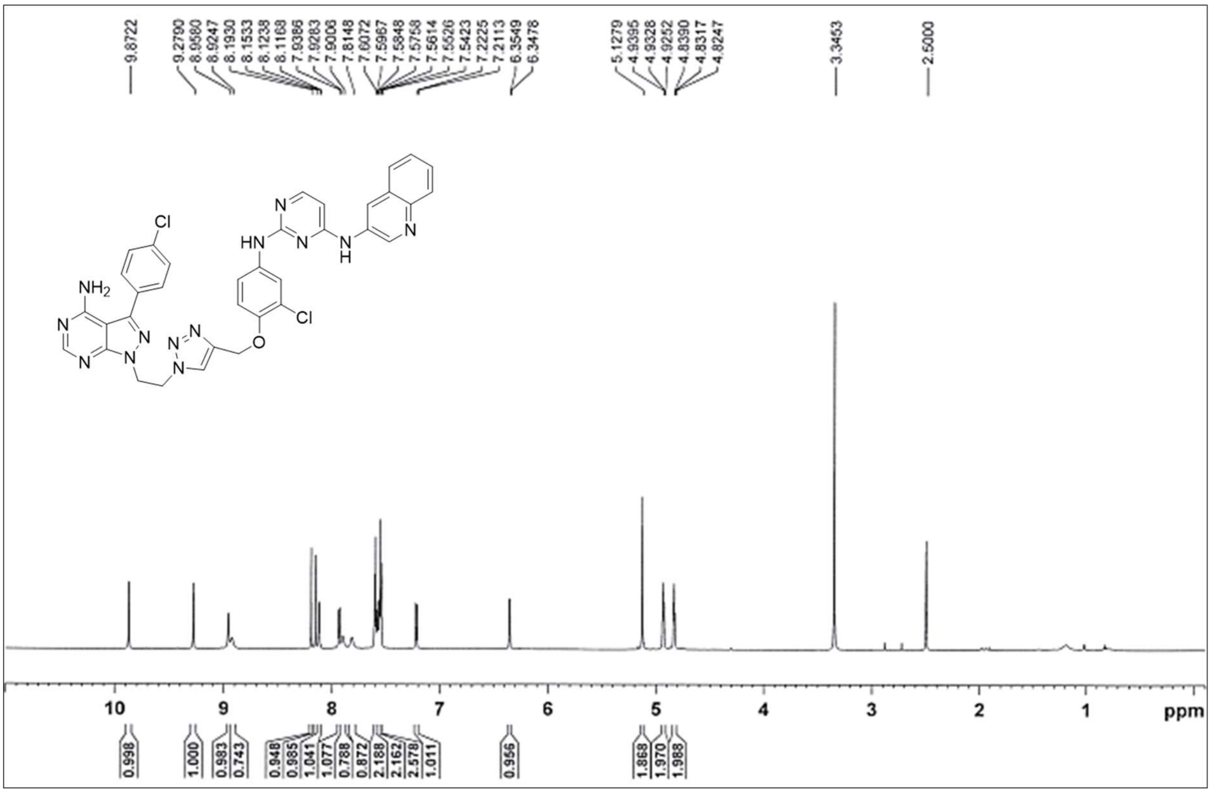

^{13}C NMR of compound **4g** (200 MHz, DMSO-d_6)

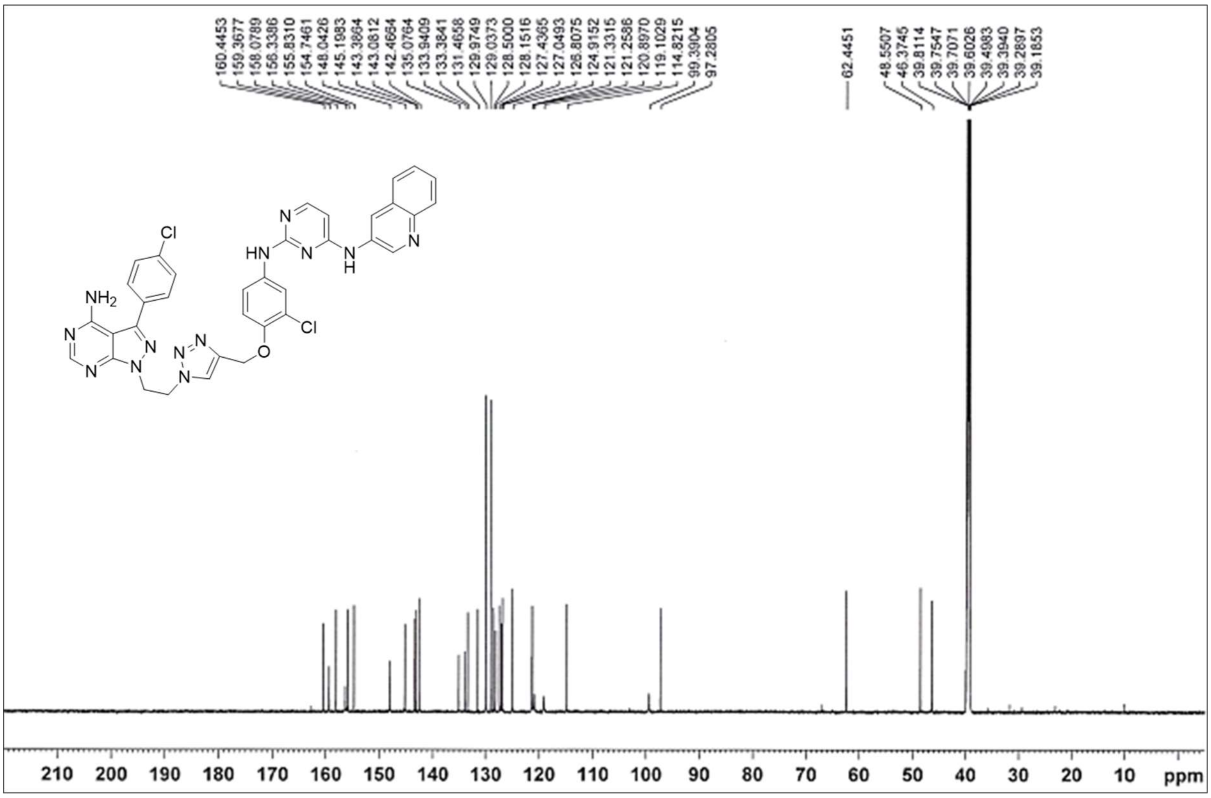

^1H NMR of compound **4h** (600 MHz, DMSO-d_6)

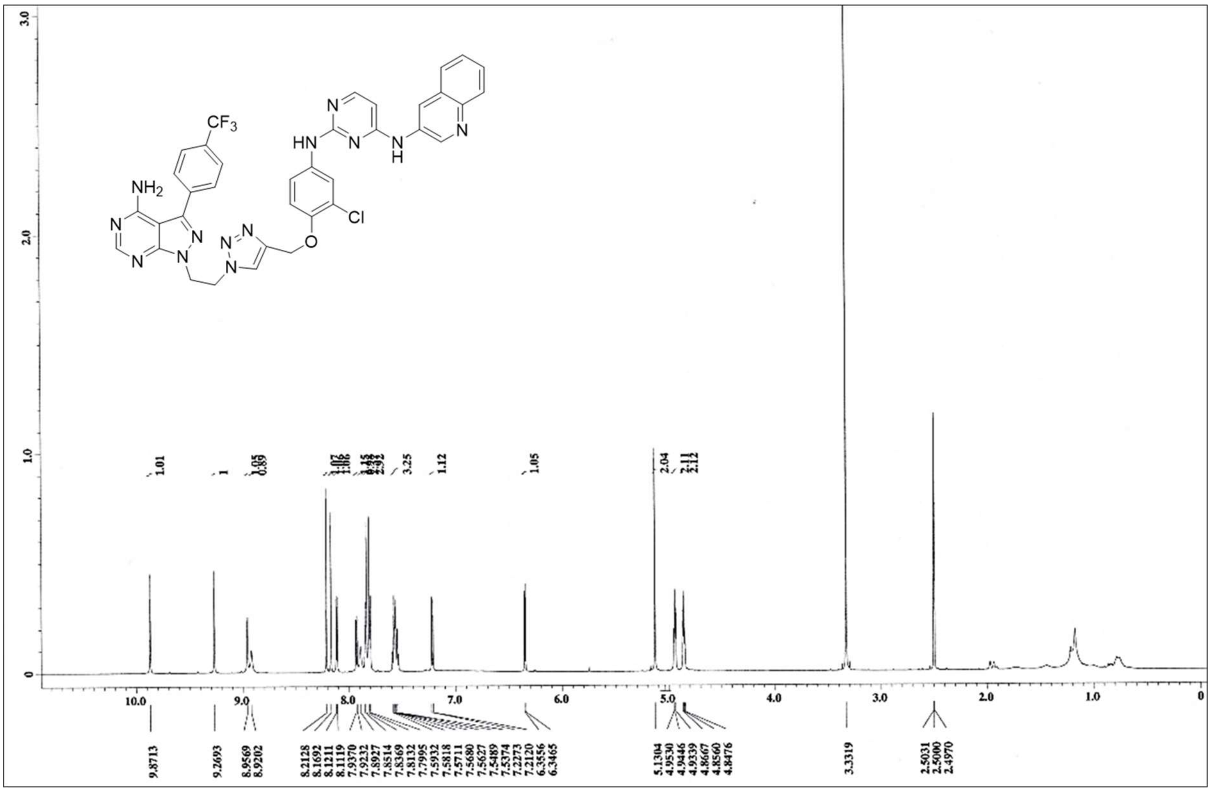

^{13}C NMR of compound **4h** (150 MHz, DMSO-d_6)

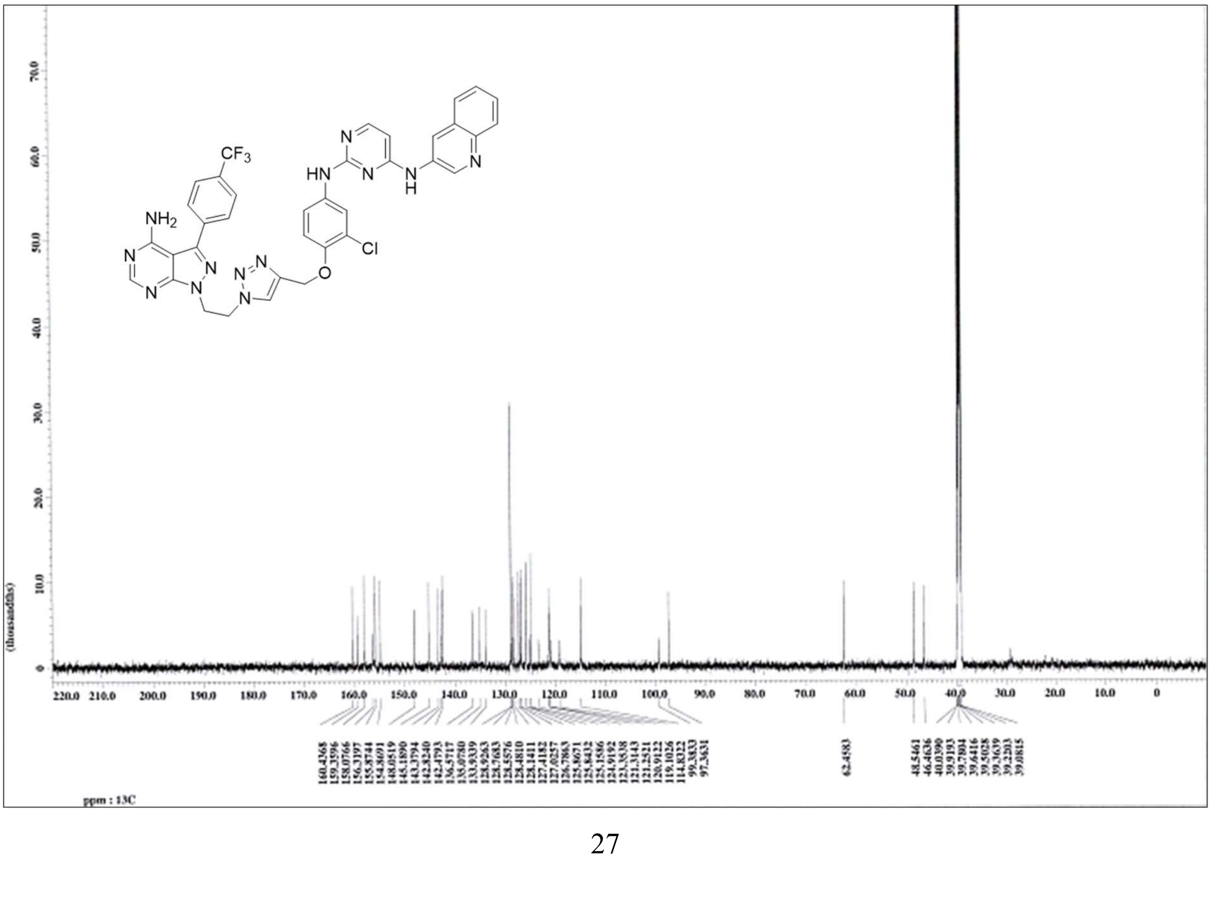

^{1}H NMR of compound **4i** (400 MHz, DMSO-d_6)

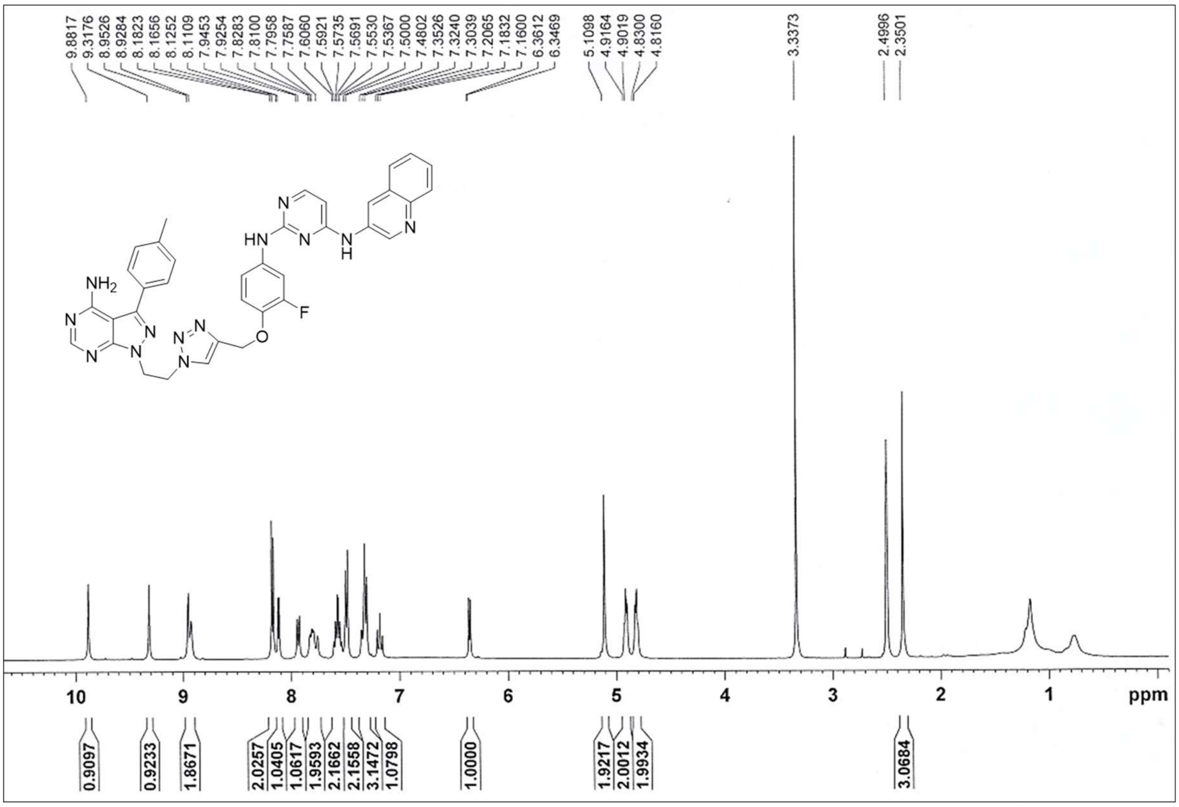

^{13}C NMR of compound **4i** (100 MHz, DMSO-d_6)

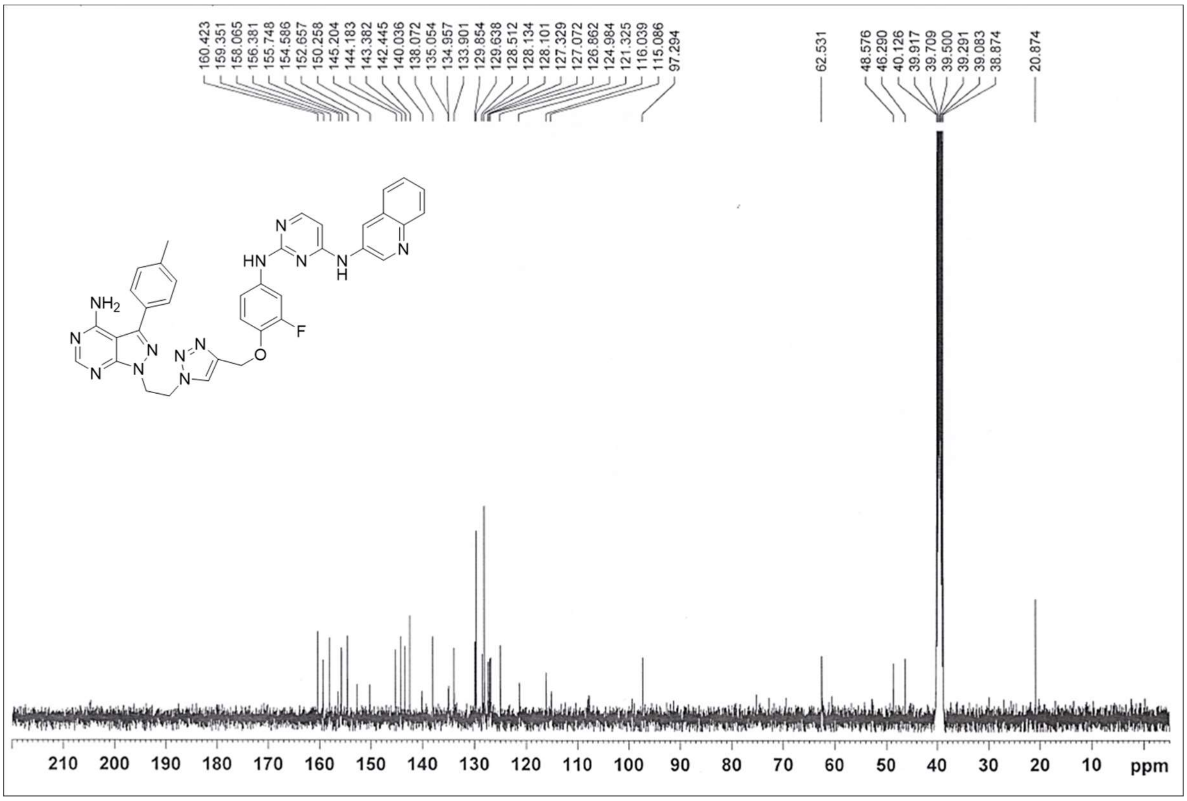

^{1}H NMR of compound **4j** (400 MHz, DMSO-d_6)

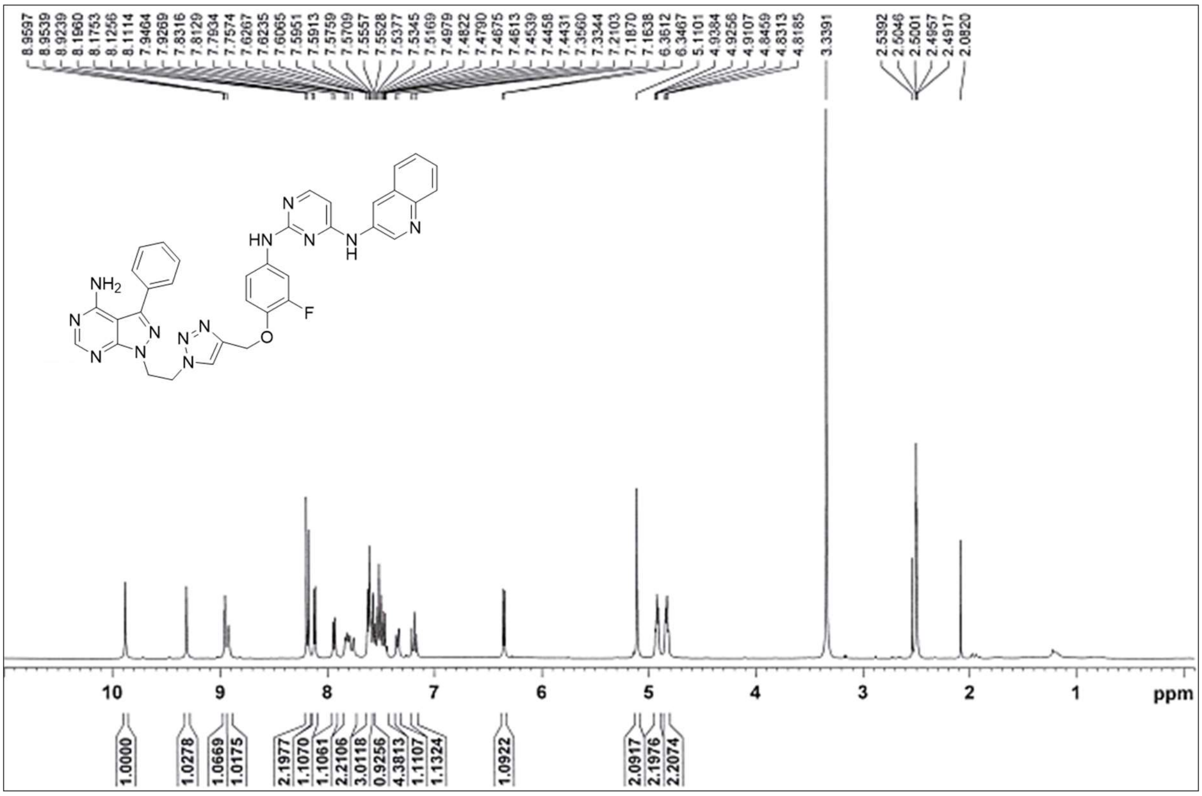

^{13}C NMR of compound **4j** (100 MHz, DMSO-d_6)

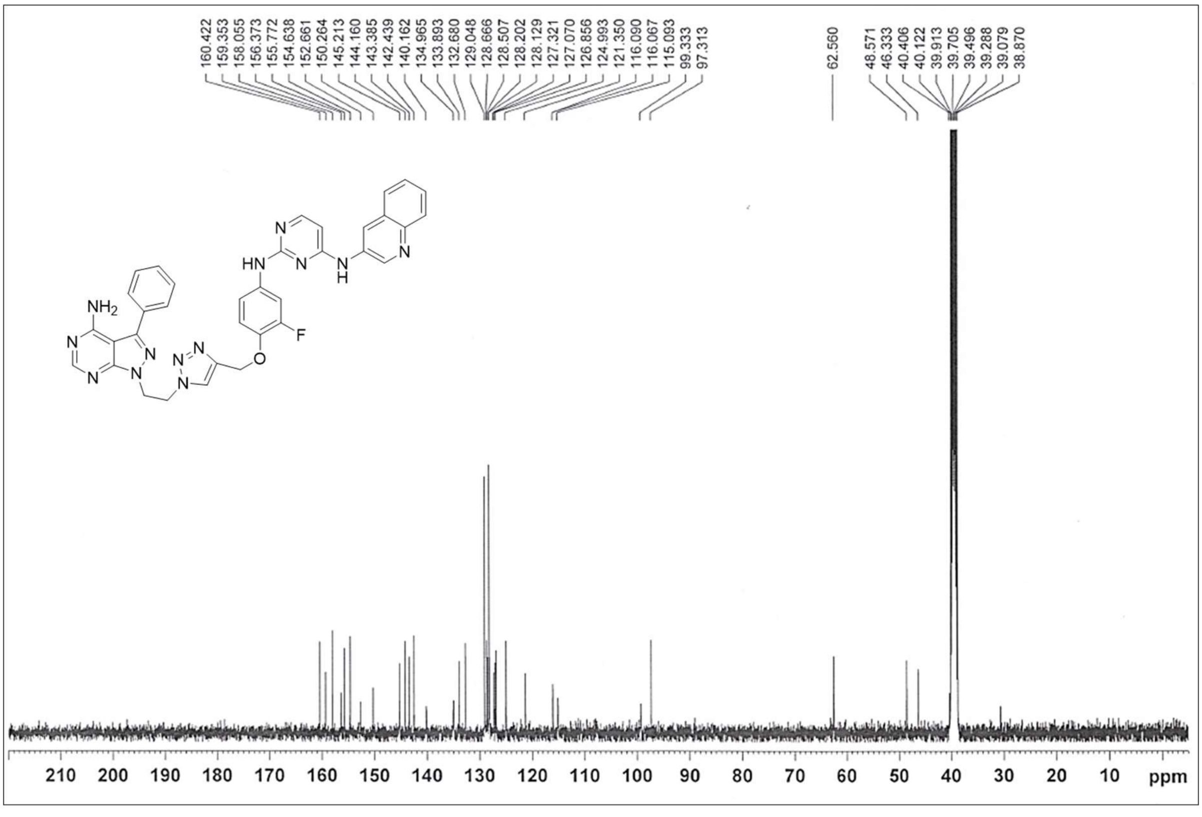

^{1}H NMR of compound **4k** (400 MHz, DMSO-d_6)

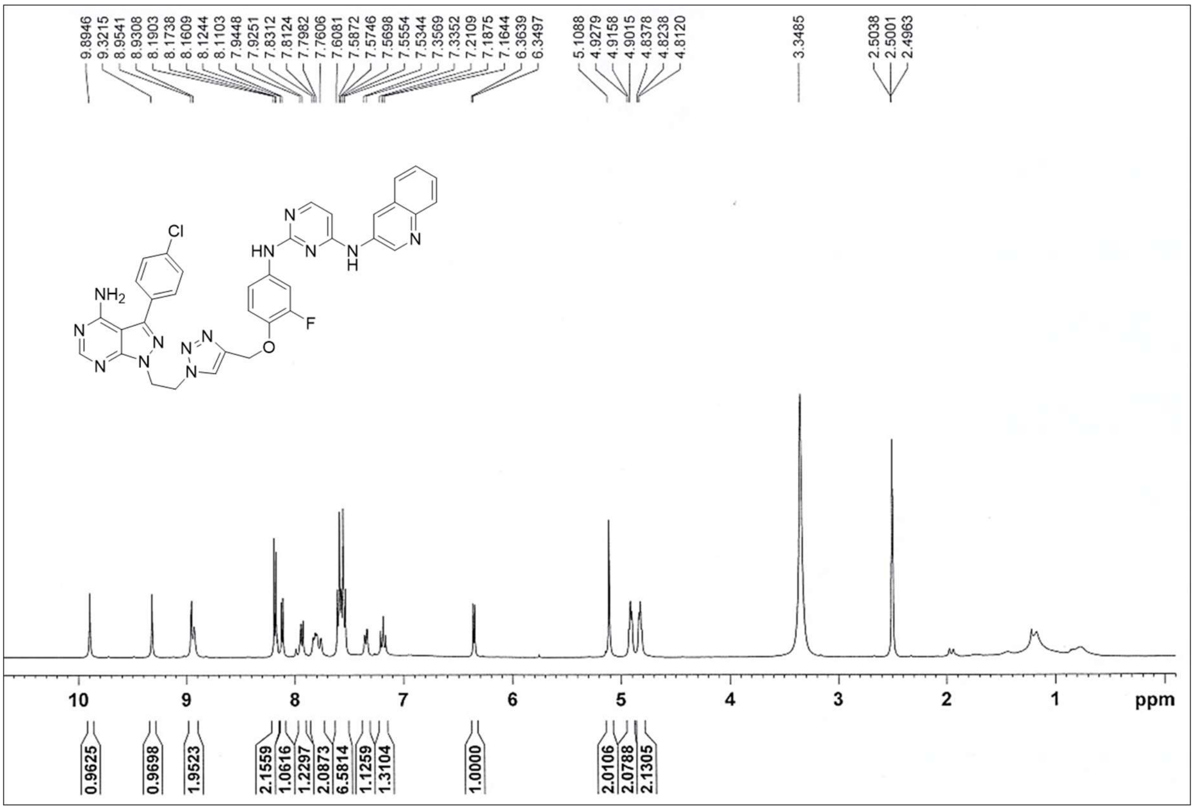

^{13}C NMR of compound **4k** (100 MHz, DMSO-d_6)

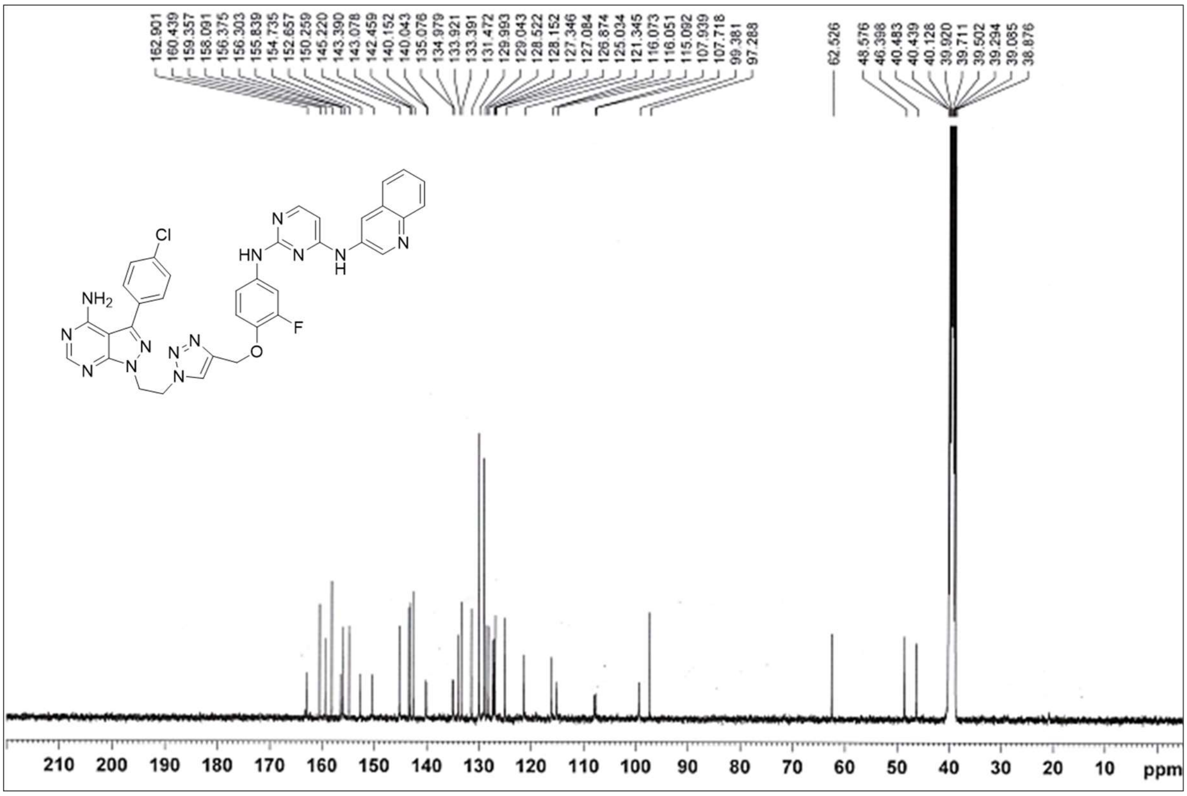

^{1}H NMR of compound **4l** (600 MHz, DMSO-d_6)

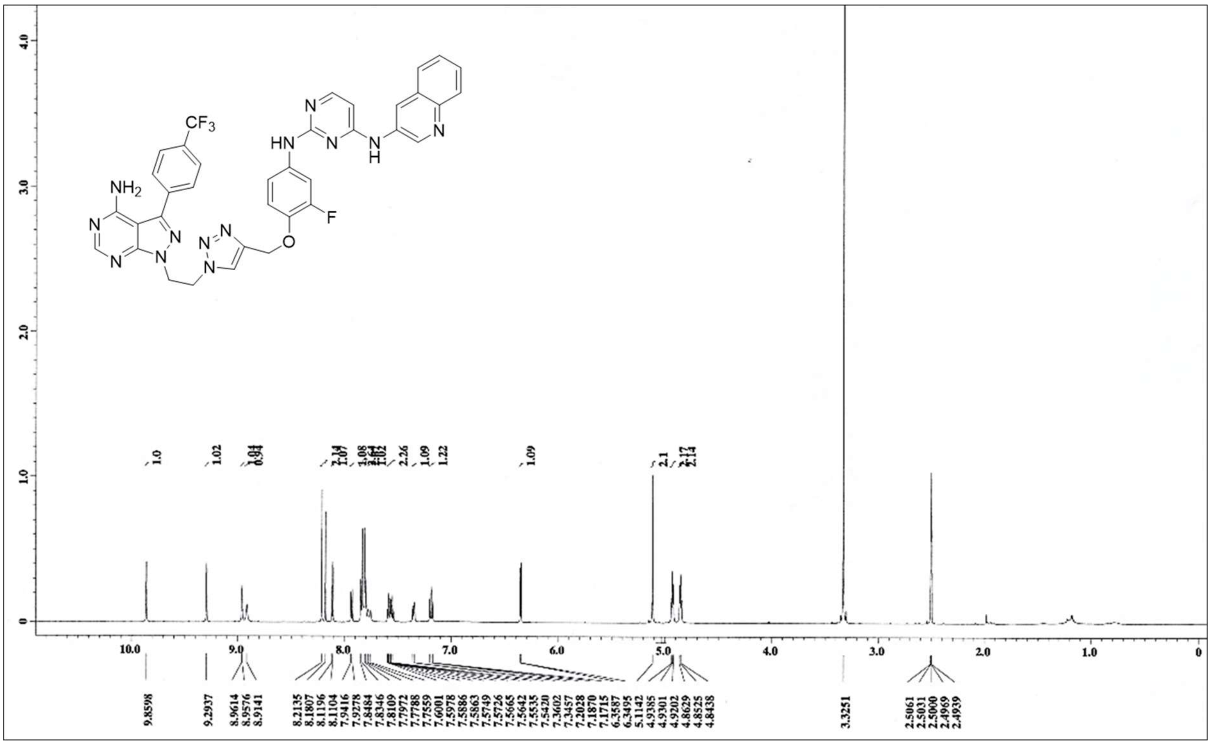

^{13}C NMR of compound **4l** (150 MHz, DMSO-d_6)

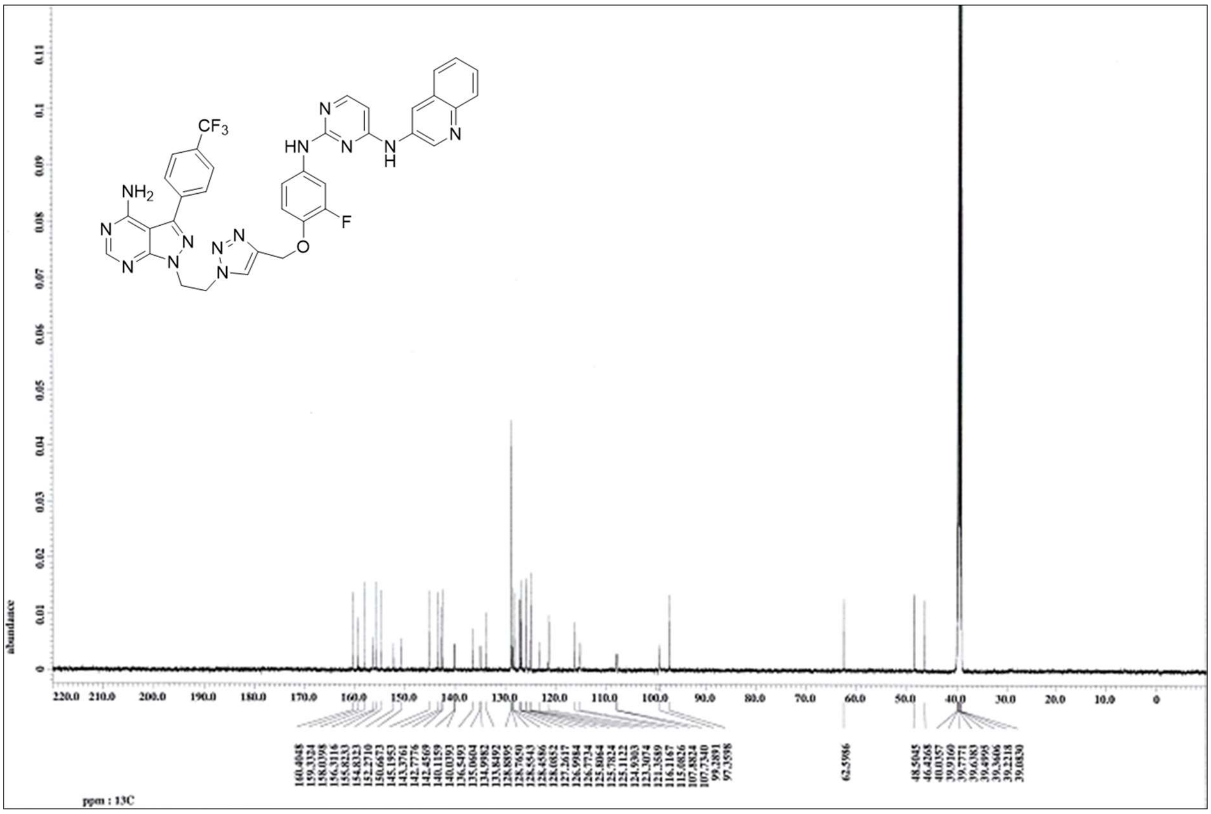

^{1}H NMR of compound **5a** (800 MHz, DMSO-d_6)

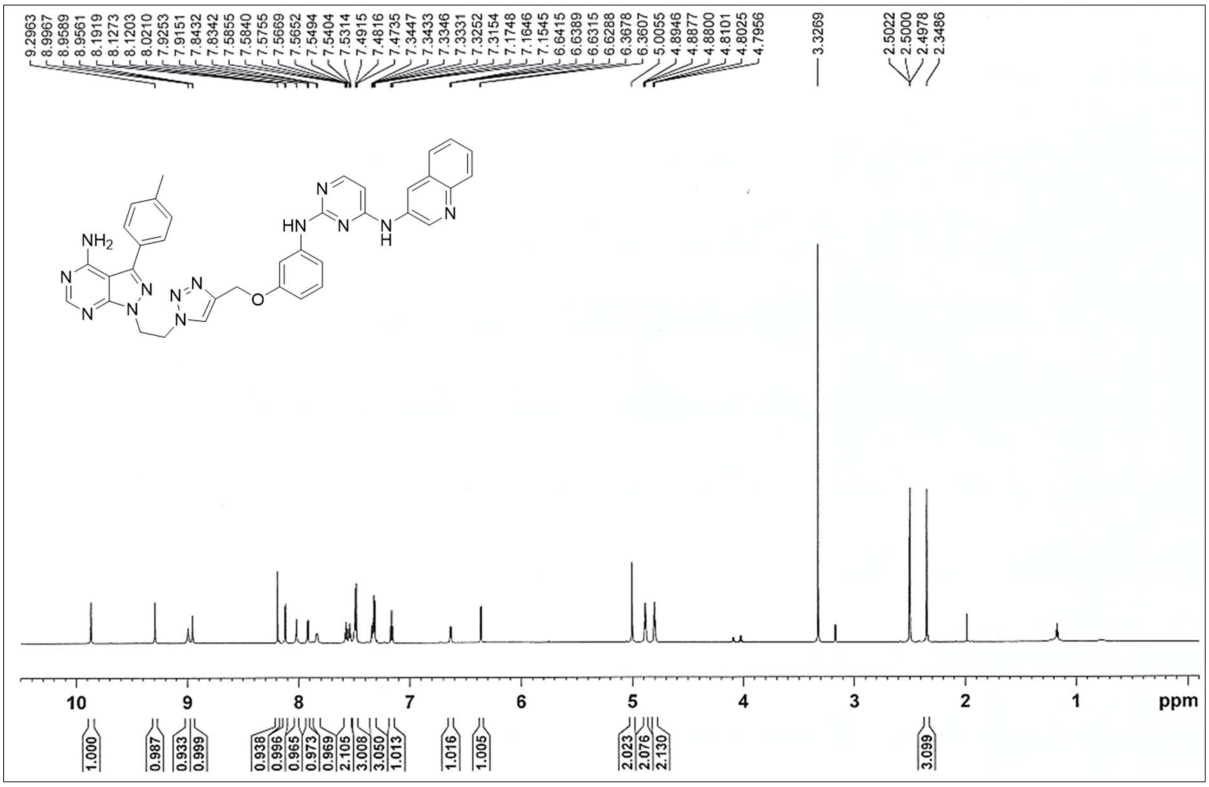

^{13}C NMR of compound **5a** (200 MHz, DMSO-d_6)

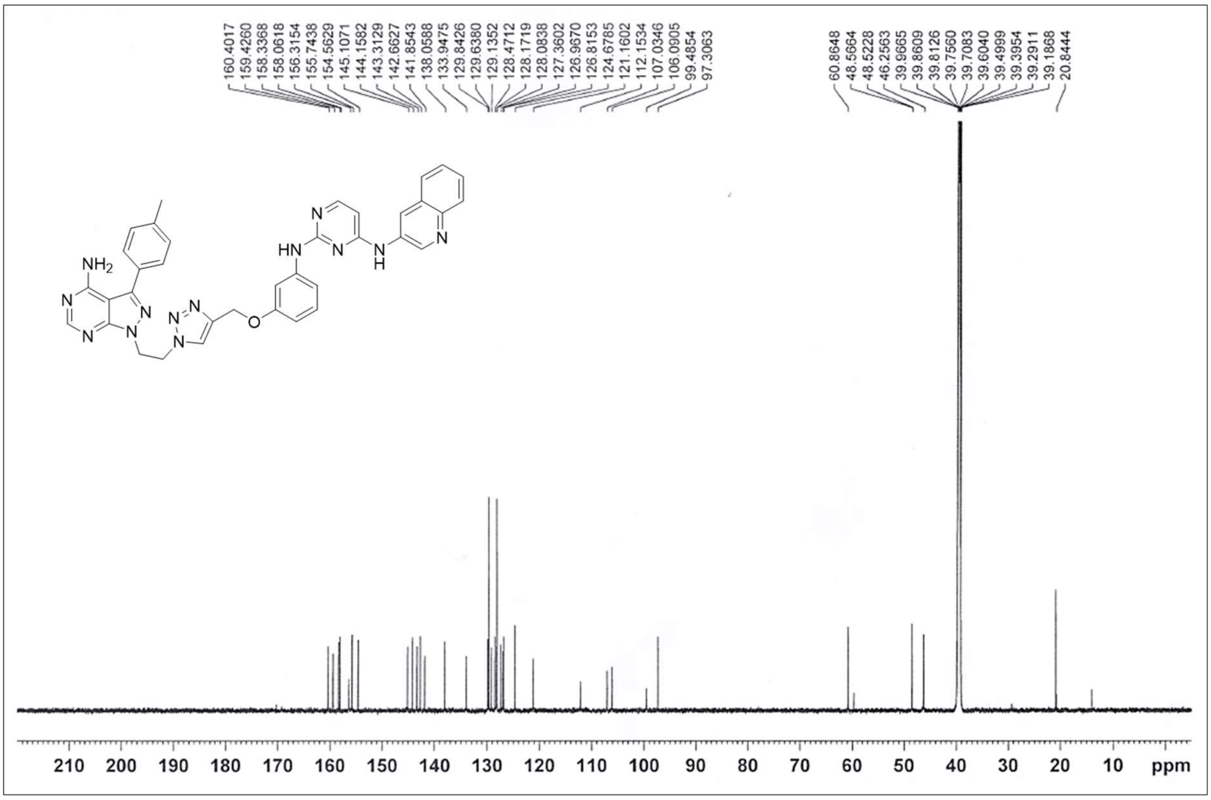

^{1}H NMR of compound **5b** (800 MHz, DMSO-d_6)

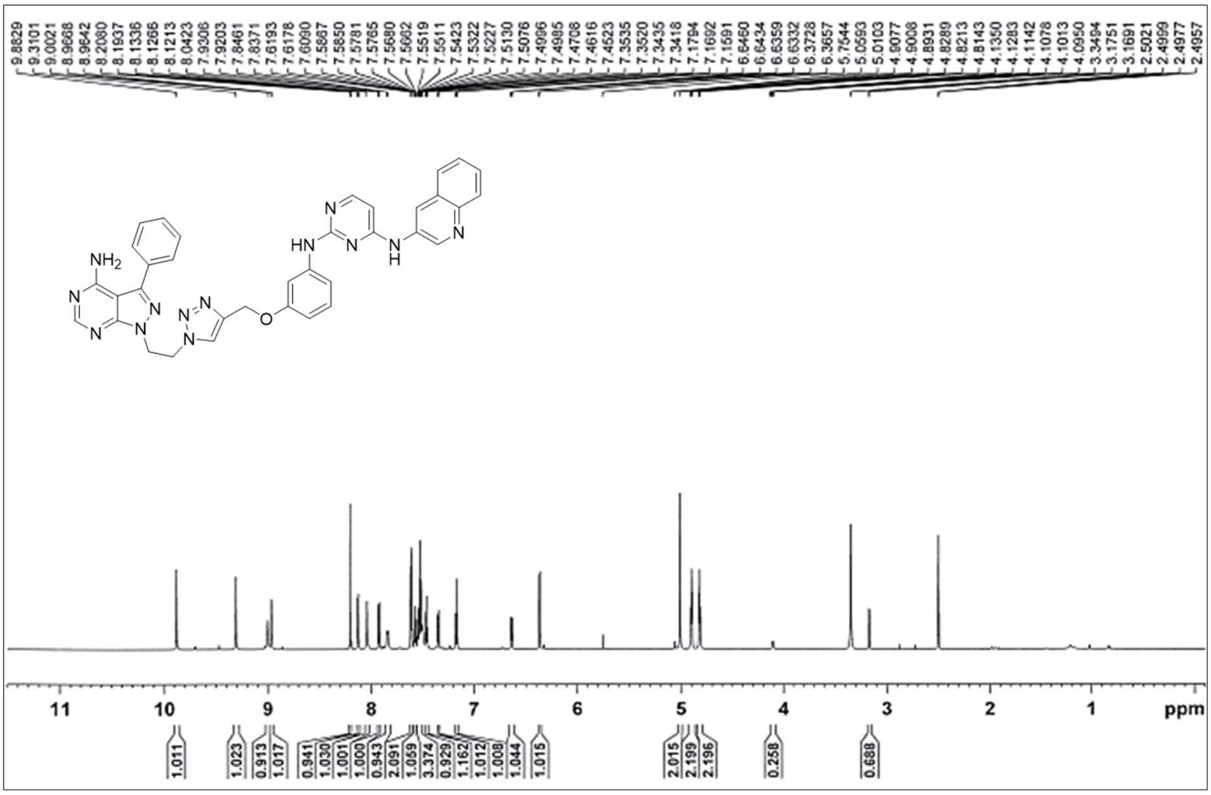

^{13}C NMR of compound **5b** (200 MHz, DMSO-d_6)

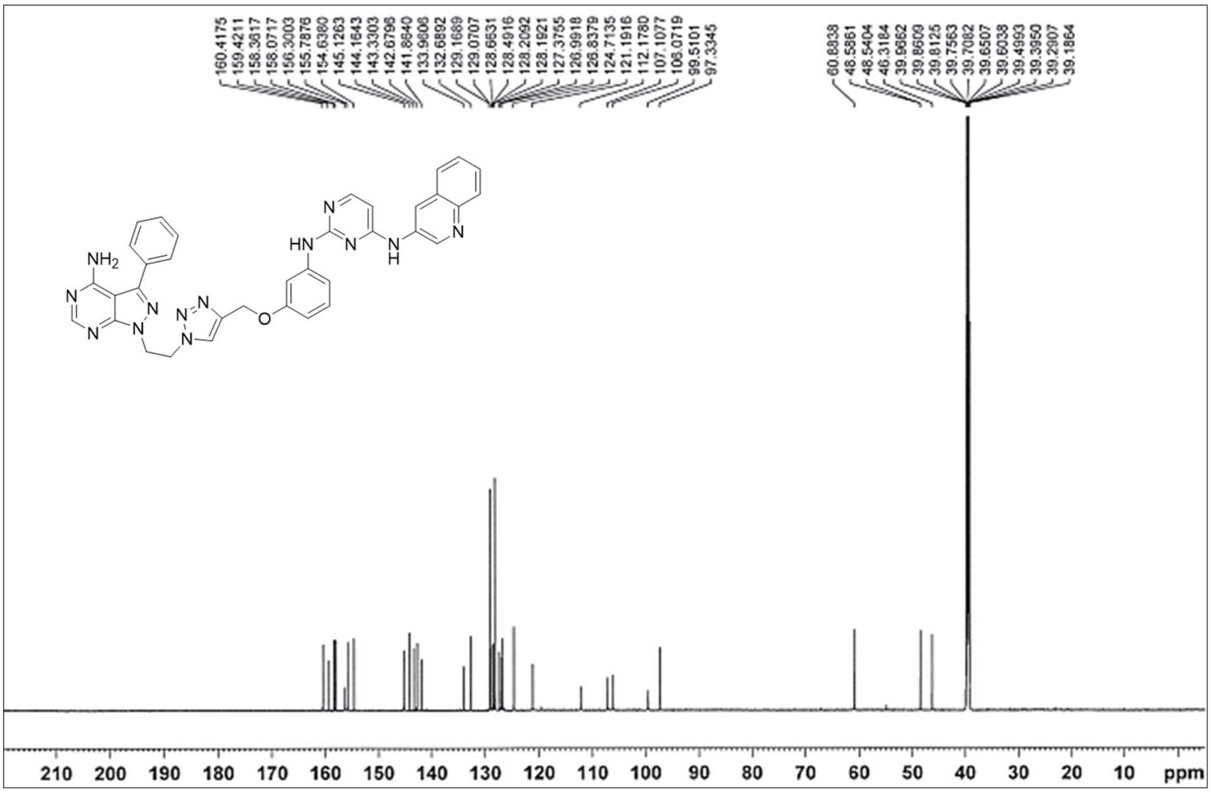

^{1}H NMR of compound **5c** (400 MHz, DMSO-d_6)

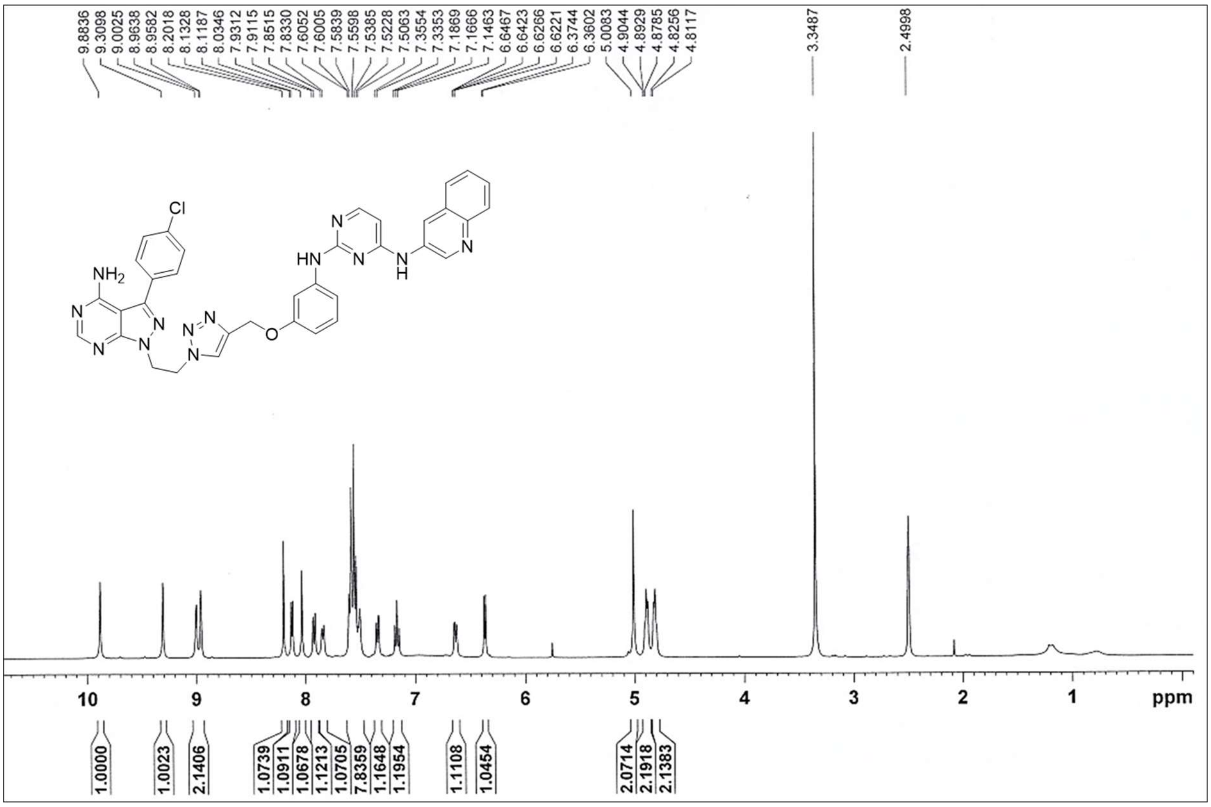

^{13}C NMR of compound **5c** (100 MHz, DMSO-d_6)

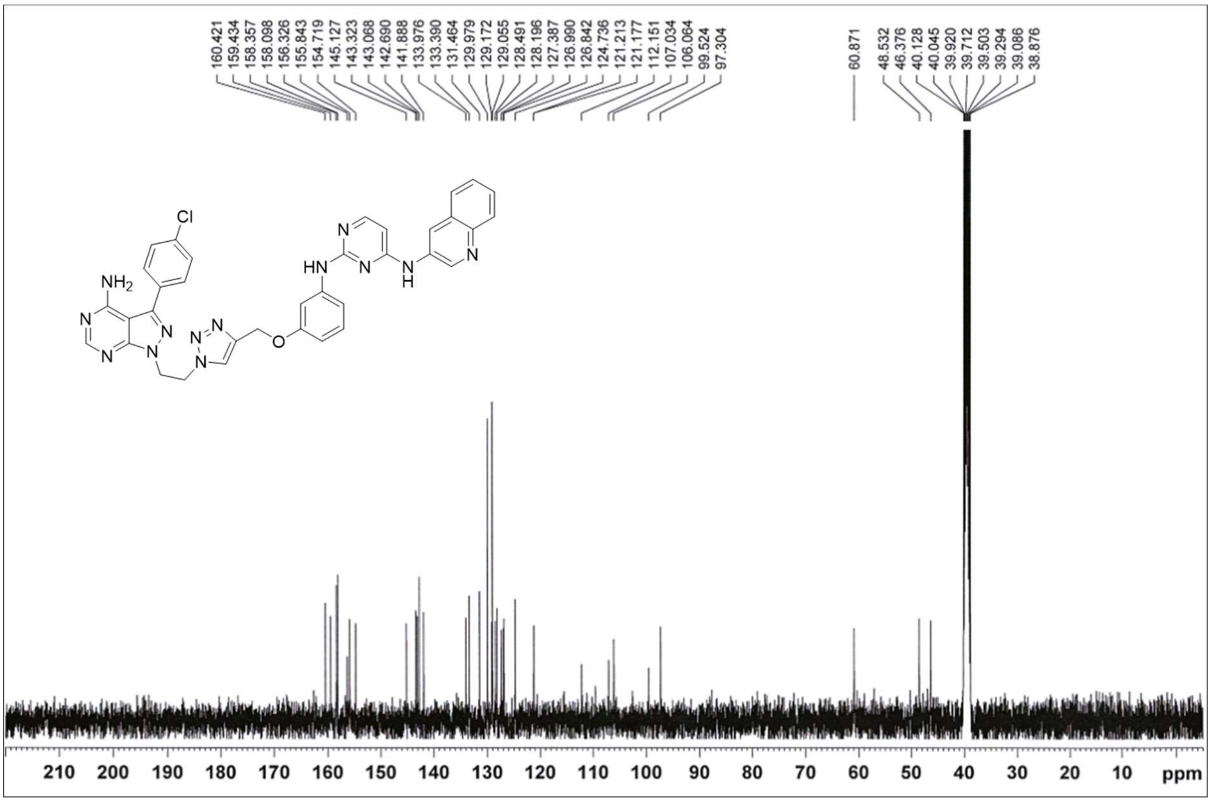

^{1}H NMR of compound **5d** (600 MHz, DMSO-d_6)

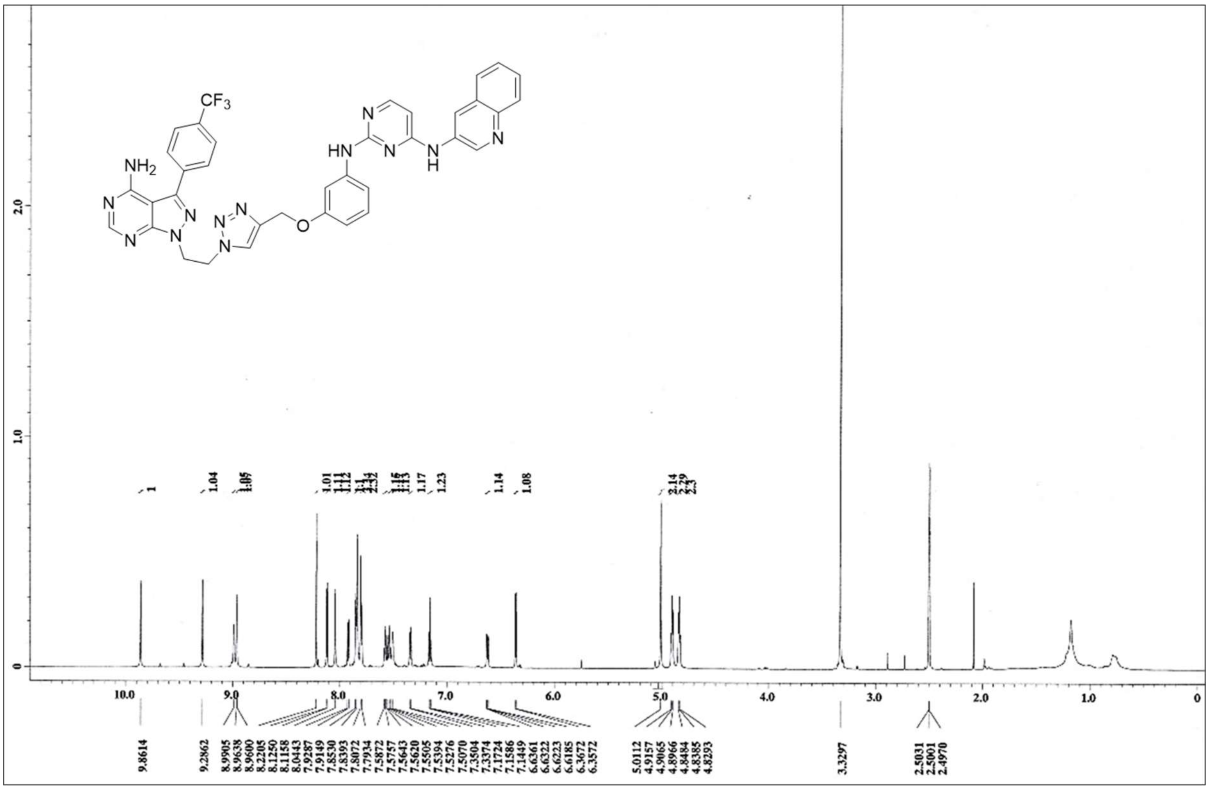

^{13}C NMR of compound **5d** (150 MHz, DMSO-d_6)

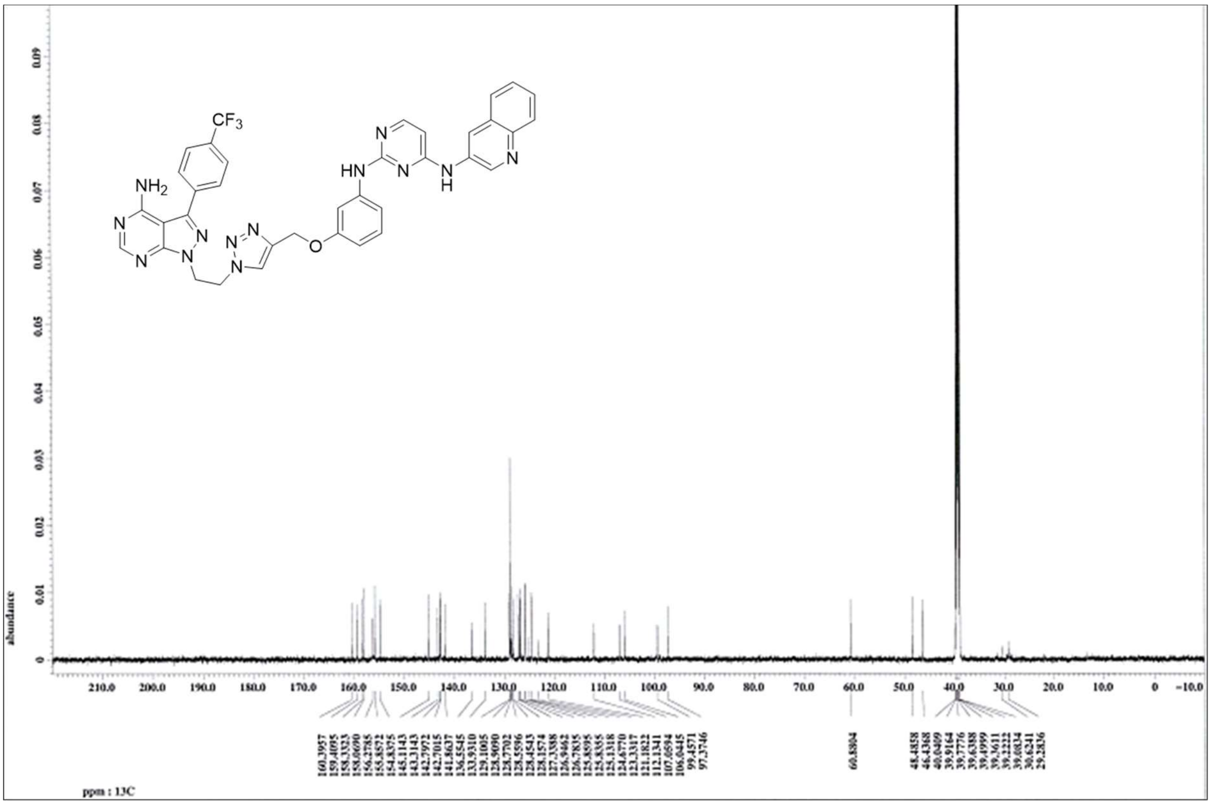

^{1}H NMR of compound **5e** (400 MHz, DMSO-d_6)

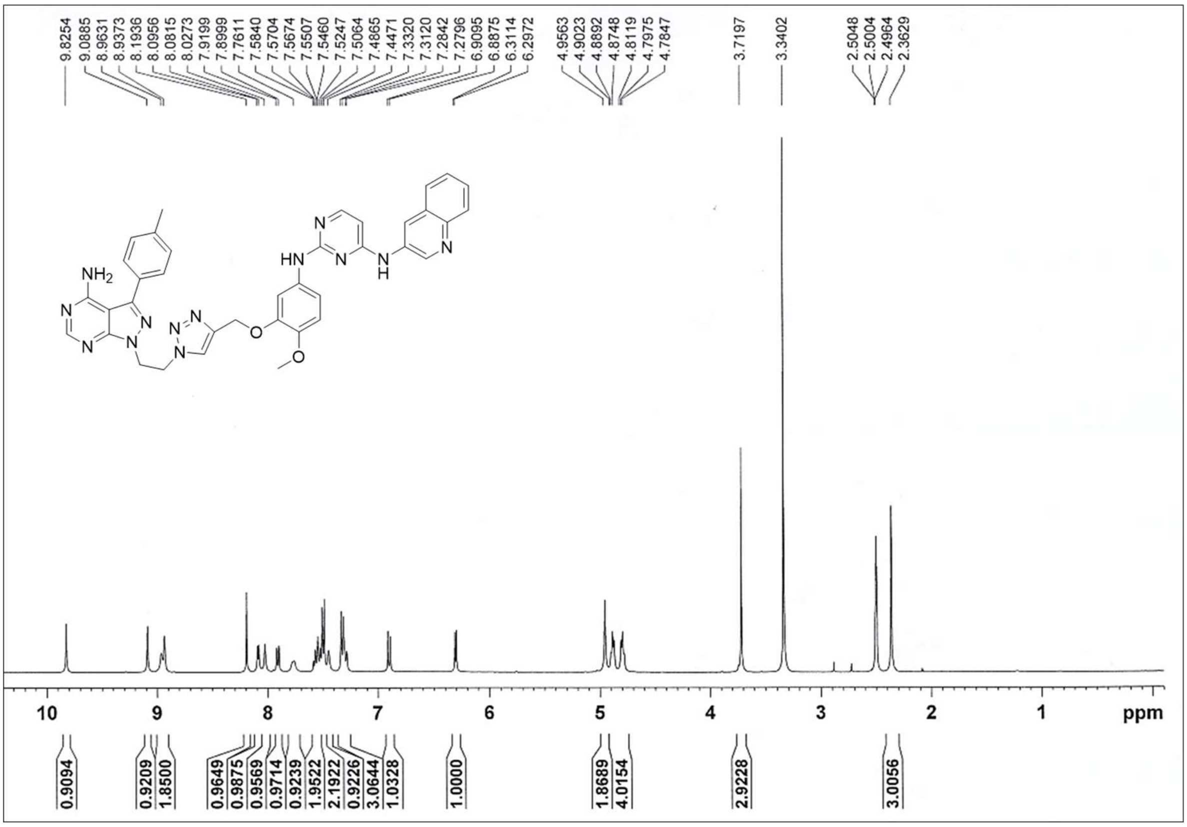

^{13}C NMR of compound **5e** (100 MHz, DMSO-d_6)

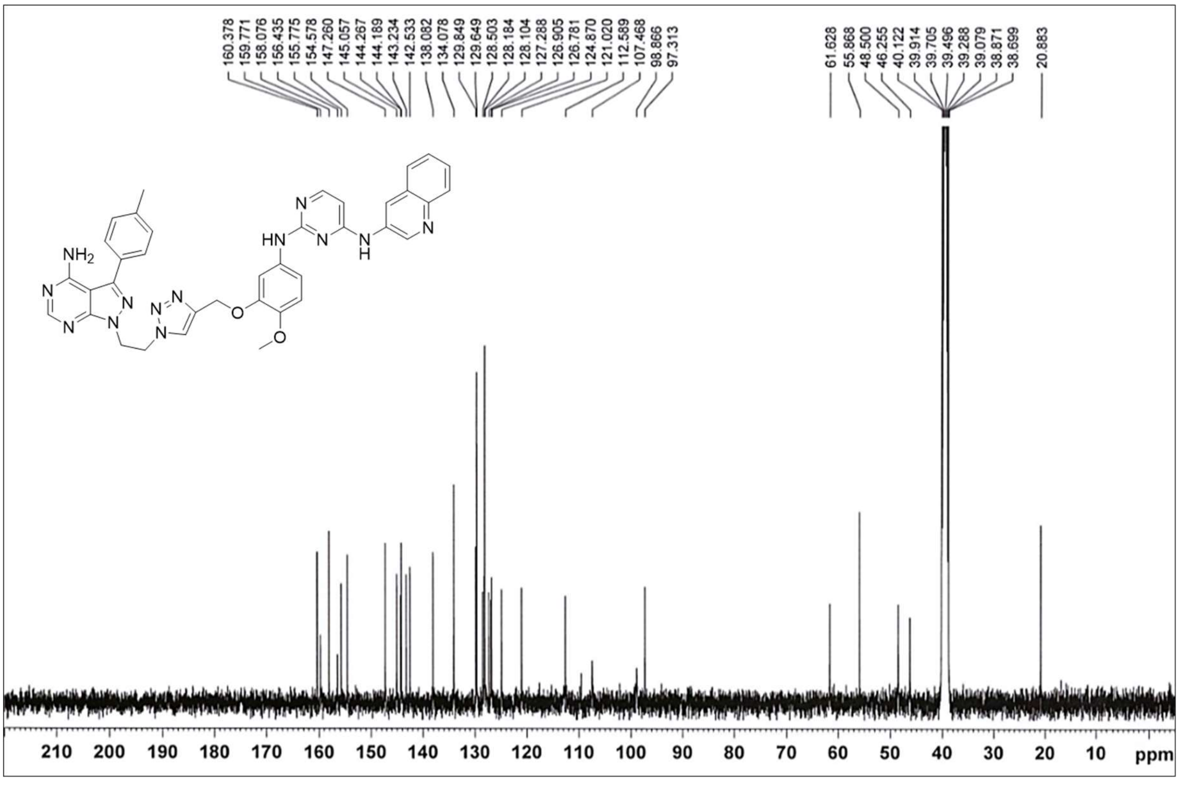

^{1}H NMR of compound **5f** (600 MHz, DMSO-d_6)

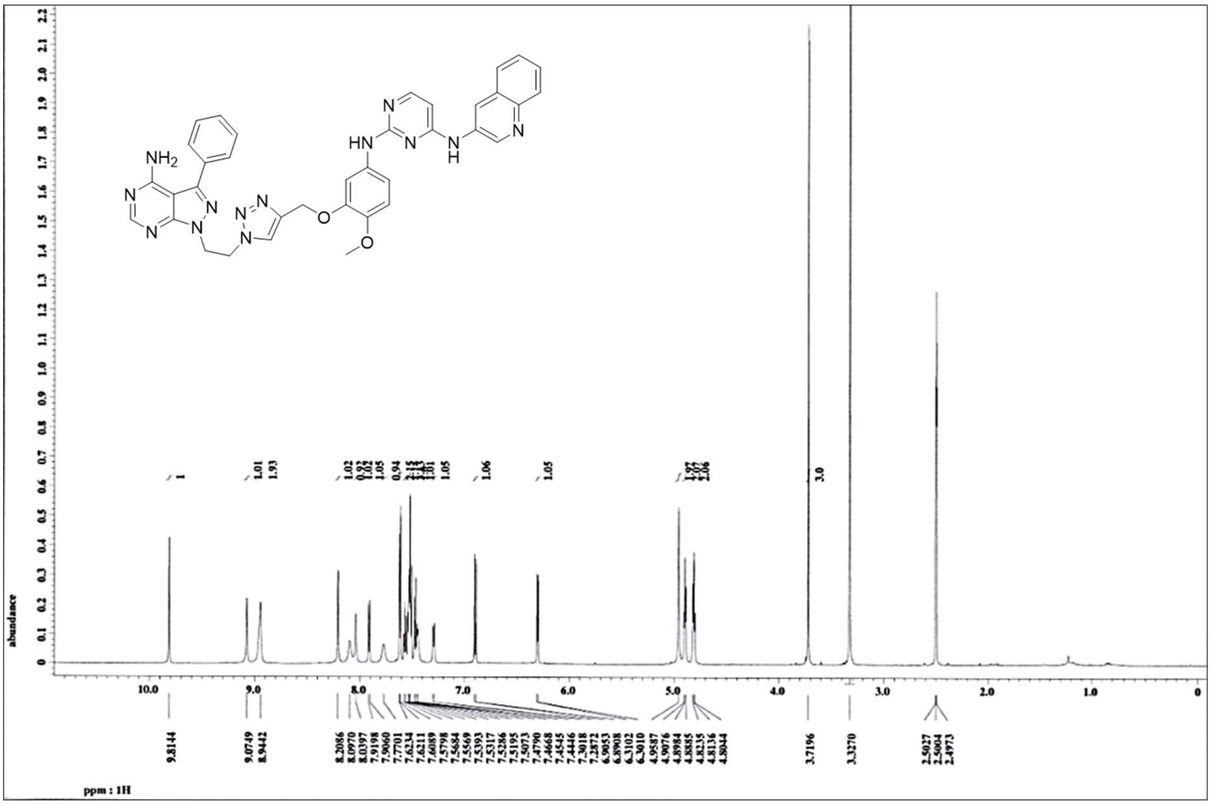

^{13}C NMR of compound **5f** (150 MHz, DMSO-d_6)

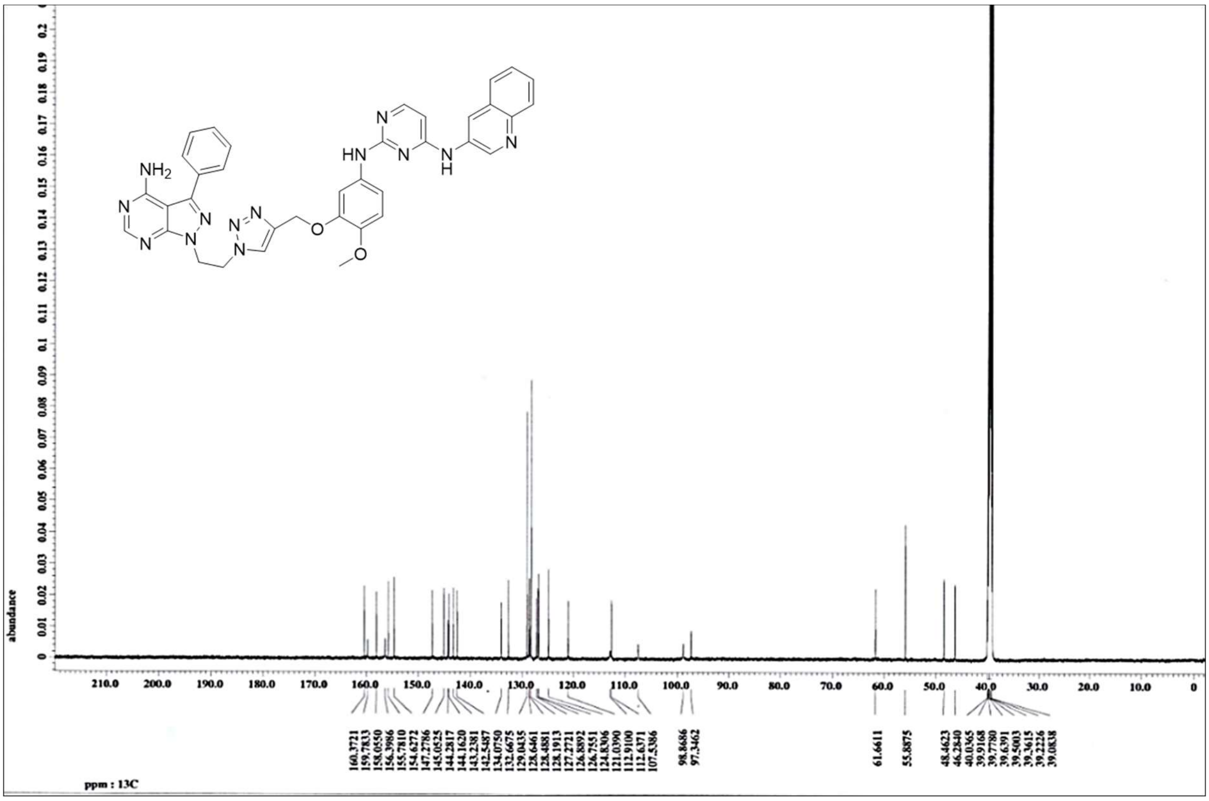

^{1}H NMR of compound **5g** (400 MHz, DMSO-d_6)

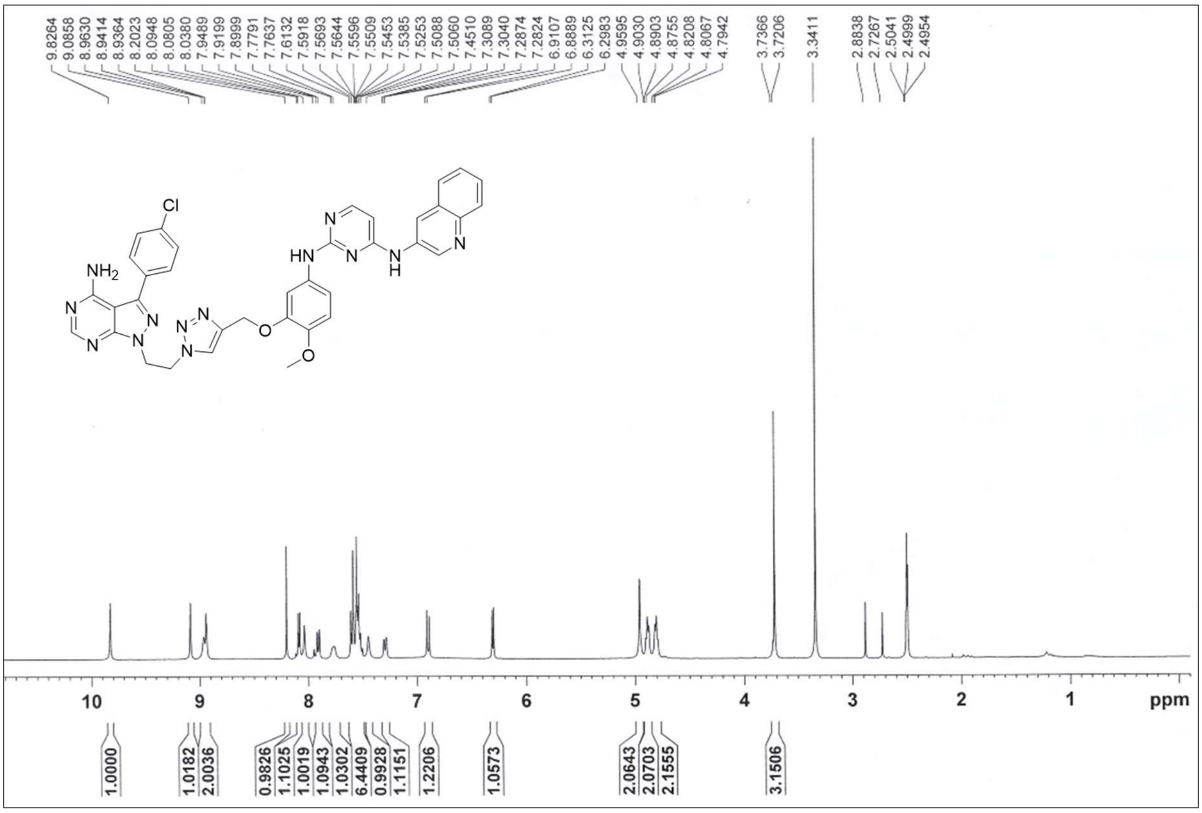

^{13}C NMR of compound **5g** (100 MHz, DMSO-d_6)

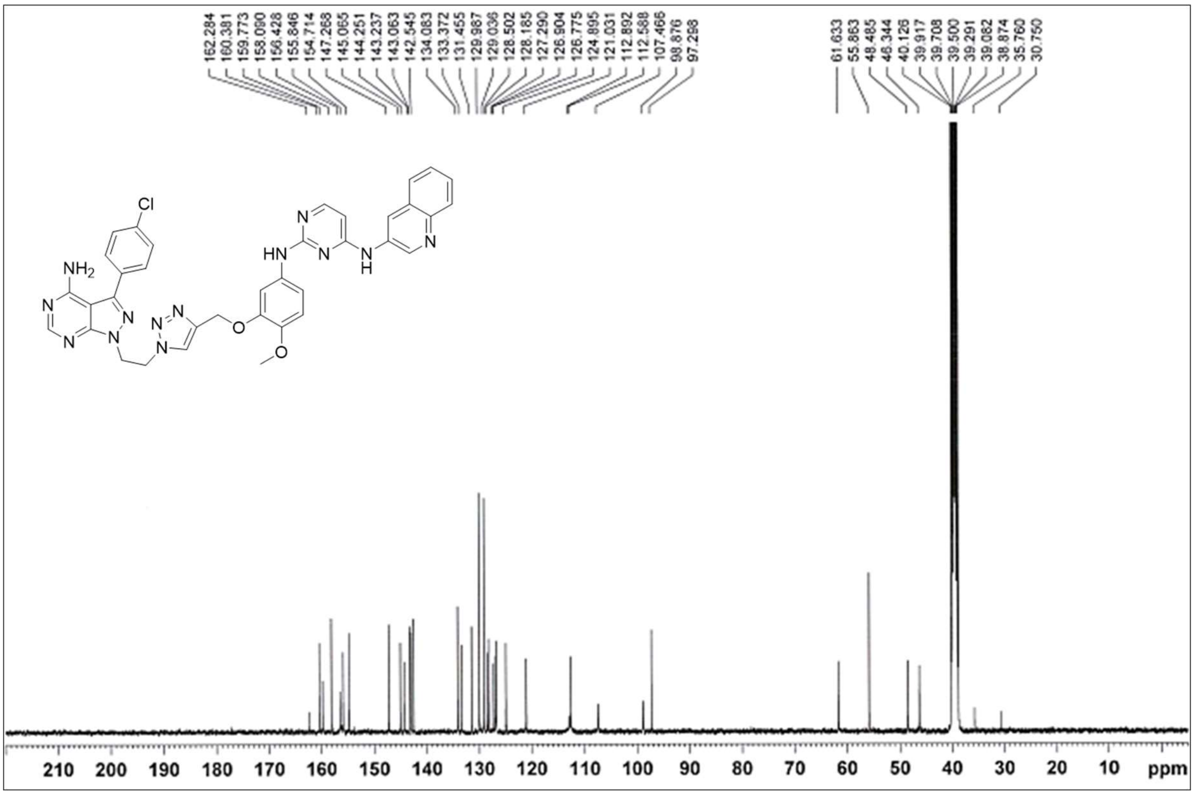

^{1}H NMR of compound **5h** (400 MHz, DMSO-d_6)

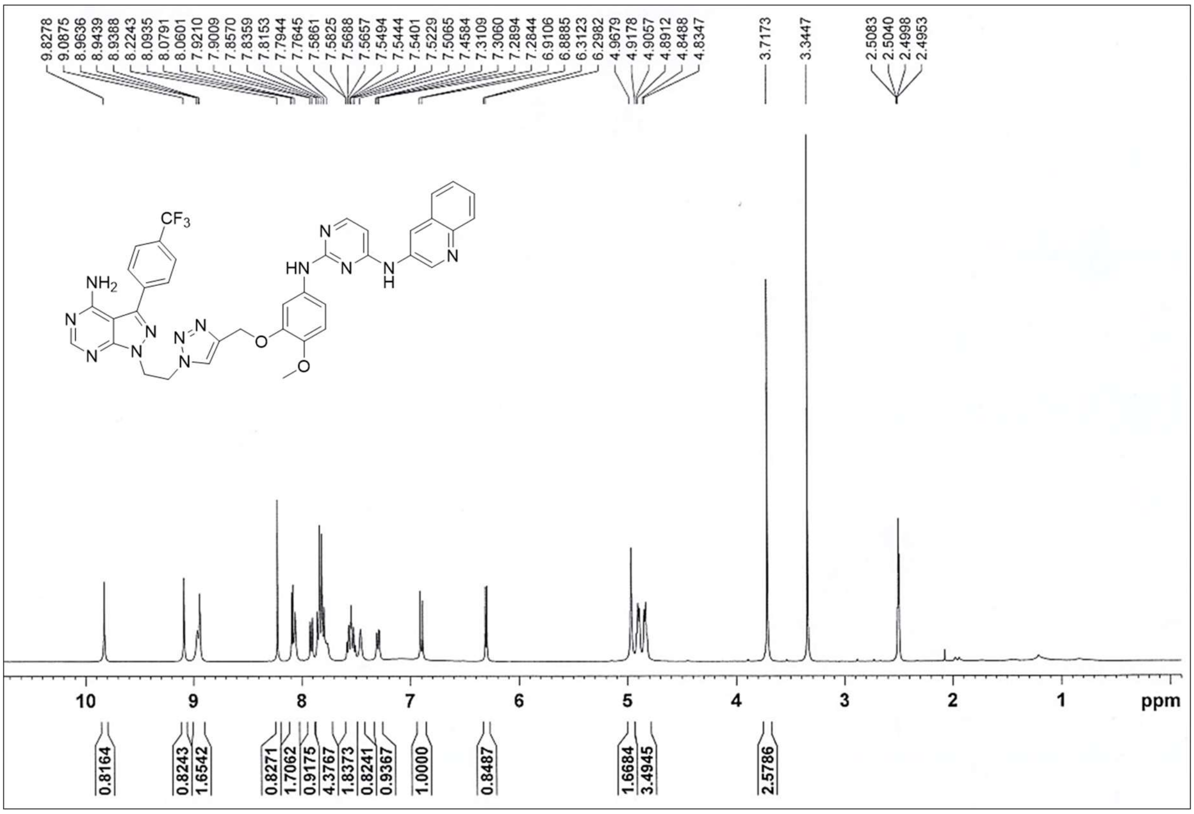

^{13}C NMR of compound **5h** (100 MHz, DMSO-d_6)

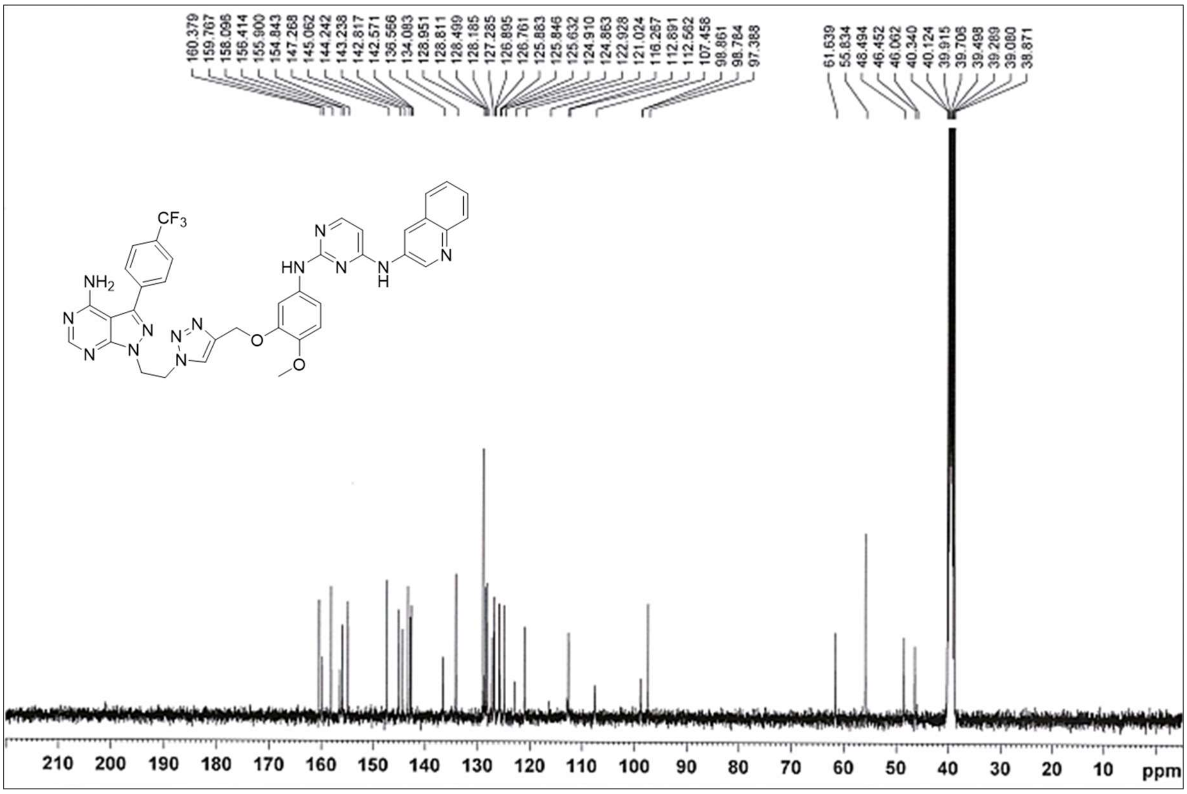

References

1. Lee, H.J., Pham, P.C., Hyun, S.Y., Baek, B., Kim, B., Kim, Y., Min, H.Y., Lee, J. and Lee, H.Y. (2018) Development of a 4-aminopyrazolo[3,4-d]pyrimidine-based dual IGF1R/Src inhibitor as a novel anticancer agent with minimal toxicity. *Molecular Cancer*, **17**.
